# Total-evidence phylogeny reveals recent crown group radiation and biogeographical history of hamsters

**DOI:** 10.1186/s12915-026-02581-z

**Published:** 2026-04-09

**Authors:** Moritz Dirnberger, Pablo Peláez-Campomanes, Tiago R. Simões, Raquel López-Antoñanzas

**Affiliations:** 1 ISEM, Univ Montpellier, CNRS, IRD, Montpellier, France; 2 Departamento de Paleobiología, Museo Nacional de Ciencias Naturales-CSIC, Madrid, Spain; 3 Department of Ecology and Evolutionary Biology, Princeton University, Briger Hall, Princeton, NJ 08544 USA

**Keywords:** Systematics, Phylogenetics, Bayesian inference, Total-evidence dating, Divergence dates, Paleobiogeography, Cricetinae

## Abstract

**Background:**

The systematics of extant hamsters (Cricetinae) have been increasingly clarified, due to advances in molecular phylogenetics. In contrast, their relationships with their fossil relatives have remained relatively unclear. Furthermore, studies on the biogeographical history and divergence times of the main groups of extant hamsters have so far been limited to molecular phylogenies and node dating approaches.

**Results:**

Here, we present the first ‘total-evidence’ analysis of hamsters that combines extinct and extant taxa, based on a comprehensive dataset covering 82 species (~ 75% of the total known diversity of 109 species). We performed a relaxed-clock Bayesian phylogenetic reconstruction and used the resulting tip-dated tree to estimate ancestral geographic ranges. Our results confirm ‘†*Kowalskia’* as a synonym of †*Neocricetodon*, support the previously suggested non-monophyly of †*Allocricetus*, †*Cricetulodon*, and ‘†*Cricetinus’* and reveal several fossil taxa as potential close relatives of the crown group. We recover a Pliocene origin of the crown hamsters, considerably younger than previous estimates of a late/middle Miocene origin. Our biogeographic reconstructions suggest a Central and Eastern European origin of the entire group, with crown hamsters emerging in the region around the Black Sea and the Eastern Mediterranean. Subsequent dispersal events into Western Europe and East Central Asia may be linked to the expansion of open vegetation.

**Conclusions:**

Based on a total-evidence phylogenetic reconstruction, we highlight necessary taxonomical revisions for several fossil cricetine taxa and explore the biogeographical evolution of the group. Importantly, our estimated divergence dates reveal a substantially younger group of crown hamsters than previously assumed.

**Supplementary Information:**

The online version contains supplementary material available at 10.1186/s12915-026-02581-z.

## Background

**Fig.1:**
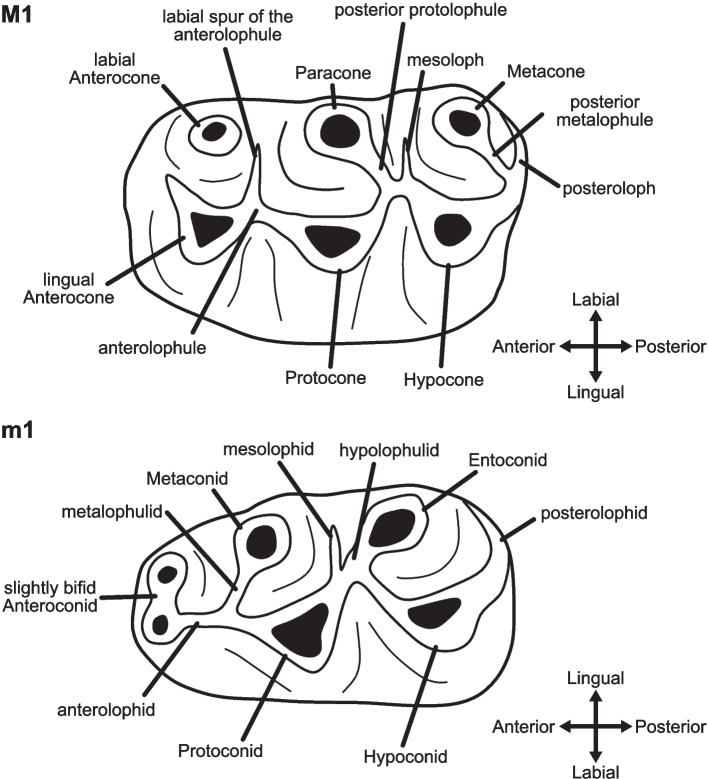
First upper (M1) and lower (m1) molar of cricetines showing the typical ‘cricetid plan’ and including the structures discussed in this study. Nomenclature and drawing following [11]

Hamsters (Cricetinae) are a subfamily of small- to medium-sized rodents within the family of ‘hamster-like’ Cricetidae [1]. They are distributed exclusively in the Palearctic, mostly in open steppe and grassland habitats or semideserts but also on agricultural lands [2]. In addition to their typical internal cheek pouches and generally short tails, they can be characterized by the morphology of their brachydont molars, with cusps organized in two longitudinal rows and usually bifurcated anterocones or anteroconids in the first molars [3] (Fig. 1). Although popular as pets and used in medicinal or behavioral studies for decades [4–6], the taxonomy and systematics of extant hamsters have been subject of debate, with novel phylogenetic hypotheses and conflicting classifications of taxa proposed in recent years [3, 7–10]. Currently, 19 extant species are accepted in either nine [9] or ten [3] genera.

Advances in extant hamster phylogenetics in the last decades have been enabled by increased availability of molecular data [12], whereas phylogenetic analyses of extinct cricetines, based on morphological data, have focused mainly on limited taxonomic sampling or specific time periods [11, 13, 14]. To date, there has been no attempt to combine fossil and extant species in one global phylogenetic analysis of hamsters, even though the number of fossil taxa exceeds the extant diversity by far. Furthermore, divergence time estimates of hamsters using molecular clocks have always been limited to node-calibrated approaches, which are based on fossil ages and/or ages taken from previous molecular estimations (e.g., [7–9, 15, 16], but see [11]).

To overcome potential downsides of node calibrations, such as errors in the assumed phylogenetic position of fossils (see, e.g., [17, 18]), fossil taxa can be analyzed with extant species by integrating their phylogenetic positions and stratigraphic ages through tip-dating [19, 20]. In this process, ages of fossils used as operational taxonomic units work as calibration points, removing the requirement of constraining calibrated nodes as monophyletic and following the placement of fossils determined by the joint inferential analysis rather than established a priori. While for extant taxa, molecular data are commonly used to reconstruct phylogenetic relationships, the incorporation of extinct taxa in the analysis requires the inclusion of morphological characters that are available for both living and fossil taxa. Thus, tip-dating is frequently applied in combined datasets where both morphological and molecular data are analyzed jointly, in a so-called total-evidence dating (TED) framework. Although there are usually far fewer morphological characters than molecular loci in such analyses, they can still have a significant impact on the resulting phylogenetic topology. For this reason, if working with morphological and molecular data, integrating both data partitions is often preferable to analyzing them separately [21, 22].

In this study, we performed the first total-evidence dating analysis for hamsters including extant and extinct cricetines, providing a comprehensive phylogenetic hypothesis of this group of rodents. Based on the resulting phylogeny, we present potential taxonomic revisions for several fossil taxa and identify the closest fossil relatives of extant hamsters, along with new estimates of the biogeographical origins and divergence dates.

## Results

### Phylogenetic reconstruction

The phylogenetic tree described here is the first to include a combination of extinct and extant cricetines and is based on the most comprehensive hamster taxon sample to date.

#### Cricetinae systematics

**Fig.2:**
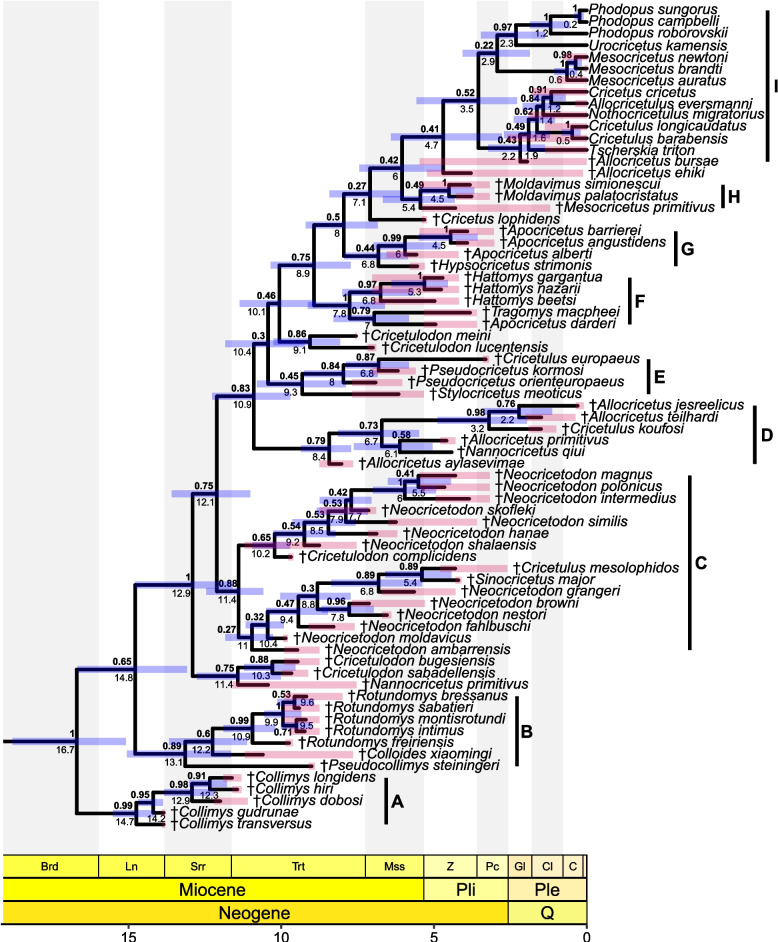
Maximum clade compatibility tree showing the ingroup of the tip-dated relaxed-clock WN Bayesian inference analysis combining molecular and morphological data (see Additional file 1: Fig. S1 for a depiction including the outgroup). Posterior probabilities (in bold) and median ages of clades are indicated at respective nodes, blue node bars indicate the 95% highest posterior density for divergence times, red tip bars indicate the stratigraphic range of the taxa. The scale axis is in Ma, the chronostratigraphic chart follows Cohen et al. [23]

In the maximum clade compatibility tree of the tip-dated, relaxed clock analysis, nine clades A–I, important for the following discussion, can be identified (Fig. 2). The earliest diverging clade A represents the genus †*Collimys*, followed by clade B, which includes three genera: †*Pseudocollimys*, and the sister taxa †*Colloides* and †*Rotundomys*. A clade of †*Nannocricetus primitivus*, †*Cricetulodon bugesiensis* and †*C. sabadellensis* precedes clade C, which comprises all species of †*Neocricetodon*, (including species of ‘†*Kowalskia*’), †*Cricetulodon complicidens*, †*Cricetulus mesolophidos* and †*Sinocricetus major*. Despite the high support for the clade itself (posterior probability (PP) = 0.88), most relationships within the clade remain uncertain. The remaining cricetines consist of several subsequently deriving lineages, the basal-most of which (clade D) includes all species of †*Allocricetus*, except for †*A. ehiki* and †*A. bursae* (the type species), as well as the taxa †*Nannocricetus qiui* and †*Cricetulus koufosi*. The poorly supported Clade E (PP = 0.45) is represented by †*Stylocricetus meoticus*, and a well-supported clade (PP = 0.84) of †*Pseudocricetus* including †*Cricetulus europaeus*. The evolutionary relationships between the clades D, E, and a small clade of †*Cricetulodon meini* plus †*Cricetulodon lucentensis* are poorly supported (PP = 0.3 and 0.46). Therefore, any of these clades are potential sister groups of the remaining cricetines. The next diverging clade F that combines all Western European insular species, is split up in two groups, †*Hattomys* on the one side and †*Tragomys macpheei* and †*Apocricetus darderi* on the other. Clade G shows a poorly supported sister relationship (PP = 0.44) between †*Hypsocricetus strimonis* and the well supported monophyletic group †*Apocricetus* (excluding †*A. darderi*). Similarly, clade H includes the well-supported genus †*Moldavimus*, as sister to †*Mesocricetus primitivus* with low support (PP = 0.49). †*Cricetus lophidens* is positioned between clades G and (H, †*Allocricetus ehiki,* I). Lastly, †*Allocricetus ehiki* is sister taxon to the crown group I, which comprises all extant cricetines and †*Allocricetus bursae*. The evolutionary relationships between clades G, H, †*Cricetus lophidens* †*Allocricetus ehiki*, and clade I are poorly supported (PP ≤ 0.42), so any of these clades could be potential sister taxa to the crown group. Within clade I, the monophyly of all genera, the sister relationship between *Phodopus* and *Urocricetus*, and the clade of *Nothocricetulus migratorius*, *Allocricetulus eversmanni* and *Cricetus cricetus* are strongly supported (PP ≥ 0.84). The positions of †*Allocricetus bursae*, *Tscherskia triton* and *Cricetulus*, diverging sequentially at the base of the latter clade, show relatively low support (PP = 0.43, 0.49, 0.62). The phylogenetic position of *Mesocricetus* is not reliably resolved (PP = 0.22).

#### Divergence times

All median divergence times following the white noise (WN) clock model are depicted in Fig. 2. The ingroup diverged at 16.69 Ma (95% highest posterior density range (HPD): 15.09–18.8 Ma). Most of the fossil genera originated in the late Miocene, whereas the earliest split in the extant group occurred around the transition from early to late Pliocene, with extant genera diverging in the Pleistocene. The estimates for the earliest splits in major fossil clades are as follows: clade A: 14.74 Ma (95% HPD: 14.16–15.51 Ma), clade B: 13.15 Ma (95% HPD: 11.64–15.05 Ma), clade C: 11.4 Ma (95% HPD: 10.59–12.46 Ma), clade D: 8.43 Ma (95% HPD: 7.71–9.35 Ma), clade E: 9.32 Ma (95% HPD: 7.89–10.8 Ma), clade F: 7.76 Ma (95% HPD: 6.61–9.13 Ma), clade G: 6.82 Ma (95% HPD: 5.86–7.94 Ma), clade H: 5.45 Ma (95% HPD: 4.33–6.68 Ma), clade I: 3.55 Ma (95% HPD: 2.28–5.58 Ma).

### Biogeographical history

**Fig.3:**
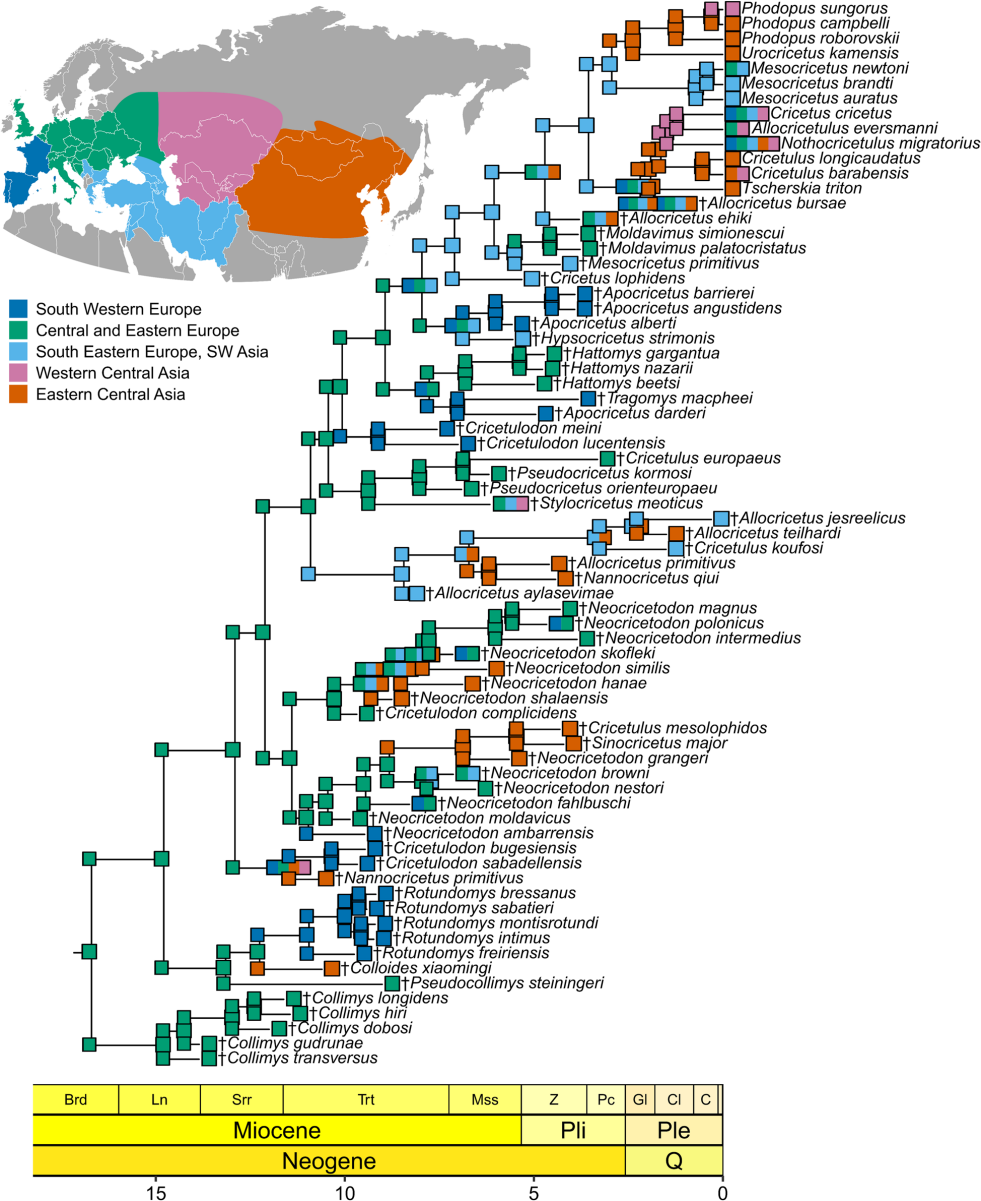
Reconstruction of the biogeographical history of Cricetinae based on the DEC + J model. Tip ranges and estimated most likely ancestral ranges are represented by colors, explained by the top left map showing the respective areas. The scale axis is in Ma, the chronostratigraphic chart follows Cohen et al. [23]

According to the dispersal-extinction-cladogenesis analysis including jump dispersals (DEC + J), the most likely inferred geographical origin of the total group is found in Central to Eastern Europe (Fig. 3), from where dispersal towards Western Europe and towards the East occurred multiple times. Entrance into Eastern Central Asia is mostly connected to a preceding range expansion to South Eastern Europe/South Western Asia. Regarding extant hamsters, the most probable area of the last common ancestor is South Eastern Europe to South Western Asia, from where two lineages dispersed towards Eastern Central Asia, where most of the extant genera subsequently diversified. The rate of anagenetic range expansion is higher than the rate of jump dispersal, and clearly higher than the rate of range contraction (Additional file 1 [24–41]).

## Discussion

### Systematics and phylogeny of Cricetinae

The final phylogeny (after removing rogue taxa, see Additional file 1) includes 71 cricetine species in 24 genera, representing an addition of 33 species in 17 genera in this study.

Compared with the results retrieved by Dirnberger et al. [11], the topology of the phylogeny remains largely consistent for the taxa they included: †*Collimys*, †*Rotundomys*, †*Neocricetodon*, †*Cricetulodon*, †*Pseudocricetus*, †*Apocricetus*, and †*Hattomys*. We confirm strong support (PP ≥ 0.84) for their six monophyletic genera (excluding †*Cricetulodon*), as the posterior probabilities of †*Collimys* (0.99 vs. 0.76) and †*Neocricetodon* (0.88 vs. 0.52) increased clearly. Differences, following from the expanded taxon set, are observed in the phylogenetic position of †*Cricetulodon bugesiensis* and †*C. sabadellensis* that diverge more basally (together with †*Nannocricetus primitivus*), and the internal relationships of †*Neocricetodon*.

**Fig.4:**
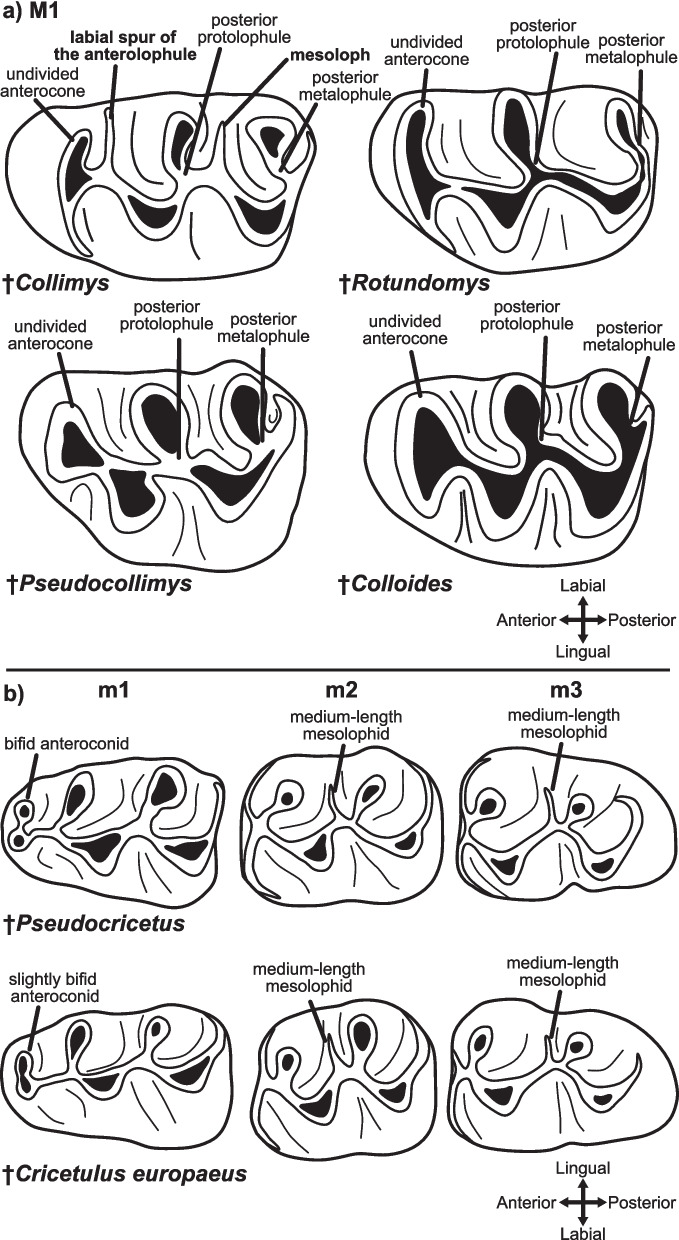
Selected dental characters important for cricetine systematics regarding **a** clades A and B, and **b** clade E. Drawings follow data in the following references: †*Collimys* [47–49]; †*Rotundomys* [38, 46, 50]; †*Pseudocollimys* [43]; †*Colloides* [42]; †*Pseudocricetus* [32, 51]; †*Cricetulus europaeus* [52]

Two monospecific genera, which were not included in the previous analysis [11], †*Pseudocollimys* and †*Colloides* (Fig. 2, clade B), have been described as morphologically similar to †*Collimys* (clade A) by the original authors [42, 43], owing to an undivided anterocone on the M1, only posterior protolophule and metalophule on M1 and M2 (Fig. 4a), as well as increased hypsodonty compared to other cricetines. These similarities led Tesakov et al. [44] to suggest that both taxa are synonymous with †*Collimys*. In our analysis, †*Pseudocollimys* and †*Colloides* clustered within the same clade as the monophyletic †*Rotundomys* (clade B). Interestingly, a relatively close relationship between †*Pseudocollimys* and †*Colloides* was already suspected by Qiu and Li [42], and similarities between †*Pseudocollimys* and †*Rotundomys* were mentioned by Daxner-Höck and Höck [45]. The main arguments for separating †*Pseudocollimys* and †*Colloides* from †*Collimys* refer to the reduced mesoloph(id)s and the absence of the labial spur of the anterolophule in the two former taxa (Fig. 4a). In †*Rotundomys*, these characteristics are also found, as are the above-mentioned single anterocone on the M1, and the only posterior protolophule and the metalophule on the M1 and M2 [38, 46] (Fig. 4a). These similarities explain the close relationship between the three genera in clade B.

While most nodes within †*Neocricetodon* (Fig. 2, clade C) remain poorly supported, the more strongly supported clades identified by Dirnberger et al. [11], such as (†*N. magnus,* †*N. polonicus*, †*N. intermedius*) and (†*N. browni*, †*N. nestori*) are also recovered here. In addition to †*Cricetulodon complicidens*, which was also previously identified as a potential member of †*Neocricetodon* [11], our analysis places †*Cricetulus mesolophidos* and †*Sinocricetus major* within this clade, as both taxa are included here for the first time. Our results thus support the earlier tentative reassignment of †*C. mesolophidos* to †*Neocricetodon*, which was based mainly on the presence of elongated mesolophids [53, 54]. In addition, †*C*. *mesolophidos* also has a labial spur of the anterolophule, typical for †*Neocricetodon* [11], albeit only of medium length [55]. In contrast to most species of †*Neocricetodon*, however, in the M2 of †*C*. *mesolophidos* a clear anterior metalophule, which is distinct from the mesoloph seems to be not always present [55]. The position of †*C*. *mesolophidos* in the tree follows consequently its elongated mesolophids. †*Sinocricetus major* likewise exhibits a medium to long mesolophid on the m1. This species was originally assigned to †*Sinocricetus* due to the presence of a mesolophid (or ‘pseudomesolophid’) on the m2 that connects to the metaconid [56]. However, in the type species †*S. zdanskyi* [42, 57–59] and in †*S. progressus* [42, 60], the mesolophid of the m2 seems either absent or weak. Since both †*S. zdanskyi* and †*S. progressus* were identified as rogue taxa and excluded from the analysis, the position of †*S. major* within †*Neocricetodon* should be considered with caution. For additional information about the potential positions of †*S. zdanskyi* in the tree, see Additional file 1.

Dirnberger et al. [11] also addressed the question of the seniority of †*Neocricetodon* over ‘†*Kowalskia*’, as demonstrated by Freudenthal et al. [61]. In line with their findings, our results recover two clades within the group, potentially supporting a distinction between the two genera, as †*Neocricetodon grangeri* and †*Neocricetodon polonicus* (the type species of †*Neocricetodon* and ‘†*Kowalskia*’, respectively) fall into different clades. However, one of these clades (including †*N. grangeri*) is not well supported (PP = 0.27), and the species composition differs from the results Dirnberger et al. [11], underlining that these clades are not reliable. The support for clade C, including all species of †*Neocricetodon*, increased (PP = 0.88 vs. 0.52), which reinforces this genus as a stable and distinct monophyletic lineage.

Recently, Feroz et al. [62] revisited the question of separating †*Neocricetodon* and ‘†*Kowalskia*’, arguing for two distinct genera. They stated that Dirnberger et al. [11] focused on “European species, such as *N. grangeri*, *N. complicidens*, and *N. progressus*” [62], p. 3) and that these species have already been rejected as ‘†*Kowalskia*’ by Qiu and Li [42]. The mentioned taxon ‘†*N. complicidens*’ (referred to by Qiu and Li [42]) was, however, not part of the analysis of Dirnberger et al. [11] (see p. 16 there for clarification). Although still not all †*Neocricetodon* species are included in our analysis, such as the recently described ‘†*Kowalskia weni*’ Feroz, Li, Fazal, Qiu & Ni, 2025, we incorporate now a broad range of taxa, including four species from China, namely †*N. hanae*, †*N. shalaensis*, and †*N. similis*, as well as †*N. grangeri*, which is in fact a Chinese species [55, 63] (potentially five, if †*Cricetulus mesolophidos* is also considered). We also want to point out that Wu and Flynn [55] emphasized the wide morphological variability within †*Neocricetodon,* encompassing ‘†*Kowalskia*’, and that Sinitsa and Delinschi [14] presented a phylogeny with both †*N. grangeri* and †*N. polonicus* undoubtedly belonging to the same clade, stating: “The congeneric status of *Neocricetodon grangeri* and *Kowalskia polonica* was clearly demonstrated by Freudenthal et al. [61] […]. Although this matter is not accepted by some specialists […], we follow most recent authors […] in recognizing the priority of the name *Neocricetodon*” (p. 773) (see also [47, 54, 64–66]). Thus, following Wu and Flynn [55] and Sinitsa and Delinschi [14], as well as the results presented here and in Dirnberger et al. [11], the seniority of †*Neocricetodon* over ‘†*Kowalskia*’ is supported.

Regarding the genus †*Allocricetus*, our results are partly consistent with those of Cuenca-Bescós [13], as they support its non-monophyly, i.e., the placement of †*A. bursae*, †*A. ehiki*, and †*A. jesreelicus* plus †*A. teilhardi* (Fig. 2, clade D) in separate clades. According to our results, †*A. aylasevimae* and †*A. primitivus*, which were described later, form a clade with †*A. jesreelicus* and †*A. teilhardi*, along with †*Nannocricetus qiui* and †*Cricetulus koufosi* (clade D). While Cuenca-Bescós [13] considered this clade to be closely related to *Nothocricetulus migratorius*, our results do not confirm such a relationship. Hír [67] noted a close morphological similarity between †*Allocricetus bursae* (clade I), the type species of †*Allocricetus*, †*A. ehiki*, and ‘*Cricetulus’ migratorius* but kept them as separate taxa. In contrast, Kowalski [68] considered †*Allocricetus bursae* to be a synonym of ‘*Cricetulus’ migratorius*. Several authors subsequently treated the genus †*Allocricetus* as a junior synonym of *Cricetulus* [69, 70]. Our analysis reveals a close affinity of †*A. ehiki* and †*A. bursae* to the extant species, but not specifically to *Nothocricetulus migratorius* (Fig. 2). Consequently, the proposed transfer of †*Allocricetus* species to *Cricetulus* (or *Nothocricetulus*) is not corroborated by our study. Since †*A. bursae* is the type species of †*Allocricetus*, but is not positioned with the remaining taxa, the establishment of a new genus name would be required for the group including most species of †*Allocricetus* (clade D).

The genus †*Nannocricetus* (with †*N. primitivus* and †*N. qiui*) was not resolved as monophyletic in our analysis. The phylogenetic position of the type species †*N. mongolicus* was uncertain, and as a result, it was identified as a rogue taxon and excluded from the final dataset. Several synapomorphies have been proposed for this genus. These include (1) a single or slightly divided anteroconid on the slender m1; (2) short and transverse metalophulids and hypolophulids on the lower molars; (3) a general absence of mesoloph(id)s; (4) a bilobed anterocone and the lack of a protolophule on the M1; and (5) specific shapes of sinuses and sinusids [42, 57, 71, 72]. Some of these characters are also commonly found in other cricetine genera or show considerable variability within †*Nannocricetus* itself. Moreover, when examining published figures of †*N. mongolicus* ([57], pl. 5 & 6, figs. 62–69 & 79–81), we could not clearly identify consistent differences in the direction and length of meta- and hypolophulids compared to other cricetines. Nonetheless, some of these potentially diagnostic characters, such as the width of the m1, are not represented in our current character matrix. Including these traits in a future version of the matrix may help in recovering †*Nannocricetus* as a monophyletic group. For additional information about the potential position of †*N. mongolicus* in the tree, see Additional file 1.

The clade of genus †*Pseudocricetus* (Fig. 2, clade E), represented here by †*P. kormosi* and †*P. orienteuropaeus* also includes †*Cricetulus europaeus*. Xie et al. [54] provisionally reassigned †*C. europaeus* to *Tscherskia* but noted that this species shows a lower tendency to develop mesolophids on the m1 and m2 compared to *Tscherskia triton*. Specifically, a short- to medium-length mesolophid is present on most m2s, whereas the m3 consistently exhibits a mesolophid of variable length [52, 54]. This dental morphology closely resembles that of †*Pseudocricetus* [32, 61, 63] (Fig. 4b). In particular, the presence of a short mesolophid on the m2 [11] and the frequent presence of a mesolophid on the m3 [32, 73, 74]. Other characteristics and proposed synapomorphies of †*Pseudocricetus* [11, 32, 61], such as the presence of an anterior protolophule on the upper molars and a short but distinct posteroloph on the M3, are also observed in †*C*. *europaeus*. An argument against the reallocation of †*C*. *europaeus* to †*Pseudocricetus*, is the presence of a single or only slightly divided anteroconid on the m1 of †*C*. *europaeus* [52, 74, 75], compared to the bifid anteroconid of †*Pseudocricetus* [32] (Fig. 4b). This character can be variable within genera and even within species [54]. Therefore, it would be necessary to assess the intraspecific variability of this character in †*C*. *europaeus* to confirm or refute this argument. This is, however, not possible, due to the limited available material of †*C. europaeus*. Given the high support for the placement of †*C. europaeus* within †*Pseudocricetus* in the phylogeny (clade E) and the above-mentioned morphological similarities, we tentatively propose transferring this taxon to †*Pseudocricetus*.

The genus *Cricetulus* (fossils in Fig. 2, clades C, D, E) deserves special attention, due to frequent changes in its scope. Regarding the extant taxa, this genus formerly comprised several smaller forms with similar dental morphology [76, 77], which could later be split up based on advances in molecular phylogenetics, resulting in *Urocricetus* and *Nothocricetulus* alongside *Cricetulus* [7, 12]. While the status and content of these three genera are now widely accepted [3], there are still frequently used fossil genera whose status remains controversial, as some authors consider them synonymous with *Cricetulus* (e.g., [70, 76]). One of these genera is ‘†*Cricetinus*’ Zdansky, 1928, which was synonymized with *Cricetulus* by various authors and databases [76–78]. The three fossil species included in our phylogeny, †*Cricetulus europaeus*, †*C. koufosi* and †*C. mesolophidos* were originally described as part of ‘†*Cricetinus*’ [55, 74, 79]. Recently, the type species, ‘†*Cricetinus varians*’, was identified as a chrono-subspecies of *Tscherskia*, rendering ‘†*Cricetinus*’ a synonym of *Tscherskia* [53, 54]. Of the six remaining species originally placed in ‘†*Cricetinus*’, four, †*Cricetulus europaeus*, †*C. gritzai*, †*C. janossyi*, and †*C. koufosi*, were provisionally reassigned to *Tscherskia*, whereas the other two, †*C. beremendensis* and †*C. mesolophidos*, were reallocated to †*Allocricetus* and †*Neocricetodon*, respectively [54]. Our analysis supports the transfer of †*C. mesolophidos* to †*Neocricetodon* (clade C). †*C. beremendensis*, is identified as a rogue taxon but shows a provisional position close to †*Allocricetus ehiki* (Additional file 1: Fig. S2), which is congruent with its tentative assignment to †*Allocricetus* [54]. Regarding the above-mentioned species reassigned to *Tscherskia* by Xie et al. [54], †*C. janossyi* was excluded from our analysis due to the limited material available (no M1, see [80]), as was †*C. gritzai*, which was identified as a rogue species. †*C. koufosi* falls within the clade that includes most species of †*Allocricetus* (clade D). Its provisional transfer to *Tscherskia* was mainly justified by the absence of a mesolophid on the m1 [54], a character that is, however, also absent in †*Allocricetus*. *Tscherskia* differs especially in the more frequent and longer mesolophids in the m2 and m3 from †*C. koufosi*, while most species of †*Allocricetus* (in clade D) show conditions similar to it. Finally, the position of †*C. europaeus* within †*Pseudocricetus* (clade E), together with their morphological similarities described above, hint toward a transfer of †*C. europaeus to* †*Pseudocricetus*.

The phylogenetic position of the monospecific genus †*Stylocricetus* as sister to †*Pseudocricetus* (Fig. 2, clade E) is only weakly supported (PP = 0.45). Based mainly on features of the skull and mandible, especially referring to the position and shape of the incisive foramen, Topachevsky and Skorik [32] distinguished †*Stylocricetus* from †*Pseudocricetus* and other cricetines and proposed a closer affinity to *Phodopus* and ‘†*Odessamys*’ (= †*Moldavimus*) which is, however, not supported by our results. An affinity between †*Stylocricetus* and the here not included ‘†*Kowalskia complicidens*’ was suggested by Topachevsky and Skorik [32], based on the strong ectoloph (or ‘pseudoectoloph’), the crest connecting the paracone and the metacone on the upper molars, which is present in both taxa. This character was the main reason for excluding ‘†*K*. *complicidens*’ from †*Neocricetodon* (or ‘†*Kowalskia*’) in subsequent studies [14, 42, 63] and can therefore not be used to argue for an affinity of †*Stylocricetus* to †*Neocricetodon*. As this strong ectoloph is absent in †*Pseudocricetus* [32, 51, 73], its presence in †*Stylocricetus* justifies, however, the separation of †*Stylocricetus* and †*Pseudocricetus*.

In contrast to the work of Dirnberger et al. [11], †*Apocricetus darderi* is not identified as a rogue taxon but is recovered here as the sister taxon to †*Tragomys* macpheei (Fig. 2, clade F). Both species are found in Mallorca [81, 82]. Together with †*Hattomys*, they form a clade (clade F), which thus includes all Western Mediterranean insular endemic cricetines. We agree with the hypothesis of Torres-Roig et al. [81] in considering that †*A. darderi* is either a sister taxon or a direct ancestor of †*T. macpheei*. However, we cannot confirm their assignment of †*A. darderi* to †*Apocricetus*. Torres-Roig et al. [81] decided to keep both insular species in separate genera due to clear morphological differences, which are exemplified by the pronounced selenodonty and hypsodonty in †*T. macpheei* compared to †*A. darderi*. Following their reasoning and considering the results obtained here, †*A. darderi* requires a new genus name.

†*Hypsocricetus* is distinguished from other late Neogene to extant cricetines by its relatively high tooth crowns [83]. The close affinity between †*Hypsocricetus* and †*Apocricetus* in our phylogeny is only very weakly supported (Fig. 2, clade G). In addition to the lower tooth crown of †*Apocricetus*, the presence of a bifid anteroconid on the m1 in †*Hypsocricetus* further separates the two genera. In †*Apocricetus s.s.*, the anteroconid is multi-lobed and more crest-like [61]. This character had also supported the exclusion of †*A. plinii* from †*Apocricetus s.s.* in the analysis of Dirnberger et al. [11], as the latter also has a bifid anteroconid.

Following the original description of ‘†*Cricetulus simionescui*’ by Schaub [84] on the basis of three hemi-mandibles, this taxon was subsequently transferred to a new subgenus, called †*Moldavimus* Samson & Rădulescu, 1973, due to a slightly shorter m1 than in other *Cricetulus* species and an undivided anteroconid [85]. Topachevsky and Skorik [32] described new material of the species from Ukraine and transferred it to a new genus, called ‘†*Odessamys*’ Topachevsky & Skorik, 1992, together with a second species ‘†*Odessamys palatocristatus*’. Recently, Crespo et al. [86] re-examined parts of the original material of ‘†*Cricetulus simionescui*’ and transferred it to †*Allocricetus*. Unfortunately, they did not take into account the previous transfer to †*Moldavimus* or ‘†*Odessamys*’, as they only compared the material to †*Allocricetus* and †*Neocricetodon*. Our analysis supports a close phylogenetic relationship between ‘†*Cricetulus simionescui*’ and ‘†*Odessamys palatocristatus*’ (Fig. 2, clade H), and we therefore concur with the definition of a genus, as proposed by Topachevsky and Skorik [32]. Due to seniority of †*Moldavimus* Samson & Rădulescu, 1973 over ‘†*Odessamys*’ Topachevsky & Skorik, 1992, we refer to the species as †*Moldavimus simionescui* and †*Moldavimus palatocristatus*.

There are two fossil members of extant genera included in our analysis, †*Mesocricetus primitivus* and †*Cricetus lophidens*. In our results, none of them are recovered within their respective genera. However, their positions in the tree are not well supported. Both taxa were originally described from the Mio-Pliocene transition of Greece [87]. †*Mesocricetus primitivus* was described as “less specialized” than extant *Mesocricetus* taxa, which explains its basal position in our analysis, suggesting that it probably does not belong to *Mesocricetus*. †*Cricetus lophidens* was later considered an “enigmatic” taxon, and its classification as *Cricetus* was questioned by Kälin [88] and de Bruijn et al. [89], with the latter referring to it as “*Cricetus*” in quotation marks. This interpretation is further supported by the selenodont morphology of †*C. lophidens*, which contrasts sharply with *Cricetus cricetus* [2]. Additionally, four taxa from the Pleistocene are recognized: †*C. major* (Woldřich, 1880), †*C. nanus* [59], †*C. praeglacialis* [59] and †*C. runtonensis* (Newton, 1909). The status of these taxa has been extensively discussed over the last century (see summaries in [90, 91]). The main issues concern whether they represent distinct species of *Cricetus* or subspecies of *C. cricetus* [69, 90, 92] and the possible synonymy of the two largest forms, †*C. runtonensis* and †*C. major* [93–95]. A revision of these taxa is beyond the scope of this study, and they are therefore not included in our analysis.

All above-described tentative taxonomic adjustments are summarized in Table 1.

**Table 1:** Summary of the potential taxonomic changes for extinct cricetines resulting from the phylogenetic results of this study

**Clade/taxon as originally identified**	**Resulting phylogenetic reconstruction (**Fig. 2**)**	**Proposed taxonomical changes**
†*Kowalskia*	Monophyletic clade with †*Neocricetodon* (PP = 0.88)	Seniority of †*Neocricetodon* over ‘†*Kowalskia*’ (see also [11, 14, 61])
†*Cricetulodon complicidens*	Nested within †*Neocricetodon*	Transfer to †*Neocricetodon:* †*Neocricetodon complicidens* (see also [11])
†*Cricetinus mesolophidos*	Nested within †*Neocricetodon*	Transfer to †*Neocricetodon:* †*Neocricetodon mesolophidos* (see also [53, 54])
†*Cricetinus europaeus*	Nested within †*Pseudocricetus*	Transfer to †*Pseudocricetus:* †*Pseudocricetus europaeus*
†*Cricetinus koufosi* + †*Allocricetus* (clade D)	Monophyletic clade D (PP = 0.79), distant from †*Allocricetus bursae*	Exclusion from †*Allocricetus *→ potential new genus (see also [13])
†*Cricetulodon meini* + †*Cricetulodon lucentensis*	Monophyletic clade (PP = 0.86), distant from †*Cricetulodon sabadellensis*	Exclusion from †*Cricetulodon *→ potential new genus
†*Apocricetus darderi*	Sister taxon of †*Tragomys *(PP = 0.79)	Exclusion from †*Apocricetus*, morphological distinct from †*Tragomys* → potential new genus
†*Cricetus lophidens*	Distant from *Cricetus cricetus*, position weakly supported	Exclusion from *Cricetus *(see also [88, 89])
†*Mesocricetus primitivus*	Distant from *Mesocricetus*, position weakly supported	Exclusion from *Mesocricetus*
†*Sinocricetus major*	Nested within †*Neocricetodon*	Type species of †*Sinocricetus* not included → future revision
†*Nannocricetus primitivus* & †*Nannocricetus qiui*	Non-monophyletic, sister to †*Cricetulodon* & within clade D	Type species of †*Nannocricetus* not included → future revision

#### Relationships with and within extant Cricetinae

All extant hamsters form a monophyletic clade, including a single extinct taxon, †*Allocricetus bursae* (Fig. 2, clade I). Within this clade, we recognize the same three groups (tribes according to [3]), as previous nuclear, mitochondrial and cytogenetic analyses: Urocricetini: *Phodopus* and *Urocricetus*; Mesocricetini: *Mesocricetus*; and Cricetini: *Cricetus*, *Allocricetulus*, *Nothocricetulus*, *Cricetulus*, and *Tscherskia* [7, 12, 96, 97]. The remaining genus, *Cansumys*, which is not included in this analysis due to the scarcity of available morphological and molecular data, was recently shown to also belong to the Cricetini [9]. The internal relationships within these clades follow most of the mentioned studies, as well. A difference is found, however, in the relationships between the clades, as *Mesocricetus* is recovered as sister to the Urocricetini and not to the Cricetini. The support for this relationship is, however, negligible (PP = 0.22). This uncertainty of the position of *Mesocricetus* is unsurprising, considering that most of the gene sequences are taken from two studies, in which the position of *Mesocricetus* also varied, depending on the analyzed gene [7, 12]. Additionally, there was lower support for the position of *Mesocricetus* in the concatenated tree than for other nodes (see [7], fig. S1; [12], fig. 2, node 2). The same problems arose, however, with sequences and/or genes not included here (see [7], fig. 1; [16], figs. S4, S6), as well as in an analysis of genome-wide single nucleotide polymorphisms [9]. It appears that the position of *Mesocricetus* needs to be verified in future studies.

The most frequently proposed ancestor of extant hamsters is †*Neocricetodon* [54, 98, 99], although Fejfar et al. [100] considered †*Neocricetodon* to represent a separate evolutionary lineage, which is in agreement with our results. There has been general agreement on a close relationship between †*Allocricetus* (considering †*A. bursae* and †*A. ehiki*) and the extant Cricetini, a relationship that is supported by our results. In contrast, our analysis does not confirm a close phylogenetic relationship between other extant and fossil genera, such as the relationship between *Cricetulus* and †*Nannocricetus* proposed by Qiu and Li [42] or between *Cricetulus* (which at the time also included *Nothocricetulus* and *Urocricetus*) and †*Moldavimus* suggested by Topachevsky and Skorik [32]. The latter hypothesis is reflected in our results through the affinity of †*Moldavimus* with the entire group of extant taxa. However, the phylogenetic relationships of the closest fossil relatives to extant cricetines are recovered with low support. Thus, besides †*A. ehiki* and †*Moldavimus,* also †*Cricetus lophidens*, †*Mesocricetus primitivus*, and clade G could be potential sister groups of the extant hamsters.

### Divergence dates

The estimated divergence times of the fossil genera obtained in our previous partial study [11] remain mostly unchanged but show here slightly higher precision. Regarding the crown group, our estimates are more precise and, in some cases, clearly younger than previous results based on extant molecular phylogenies with node calibrations, as shown in Table 2 [7–9, 12, 101, 102]. The largest difference can be seen in the age of crown hamsters (see also Steppan et al. [101]: 13.5–14.1 Ma; Romanenko et al. [102]: 11.2–12 Ma; Jiang et al. [8]: 12.73 Ma). To explore the reasons for these differences, the calibration points of these studies are examined. According to Romanenko et al. [102], their only calibration point, regarding the root of Cricetidae, was taken from Lebedev et al. [103], who focused on Arvicolinae and the genetic variation of *Ellobius*. The two calibration points used by Neumann et al. [12], i.e., the splits between *Mus* and *Rattus* (12 Ma) and between *Gerbillus* and *Tatera* (8 Ma), are outside of the Cricetinae. The former calibration point has been controversial [104, 105], and its usage has been shown to possibly lead to overestimated results [101]. Subsequent studies that estimated divergence ages of specific groups within Cricetinae were based on the possibly too old ages of Neumann et al. [12], which again influenced their results [106, 107].

**Table 2:** Divergence ages (in Ma) within crown cricetines, estimated in this and other studies

	**This study**^**a**^	**Neumann** **et al. **[12]^**b**^	**Lebedev et al. **[7]^**c**^	**Pan et al. **[9]^**d**^
Crown group	3.55 (2.28–5.58)	8.5–12.2	12.25 (10.24–14.54)	12.25 (10.24–14.54)
Urocricetini	2.32 (1.37–3.47)	-	10.37 (8.37–12.52)	11.23 (8.83–13.77)
*Phodopus*	1.19 (0.54–1.87)	4.9–6.9	5.69 (4.38–7.04)	7.45 (4.03–10.79)
*P. sungorus*/*P. campbelli*	0.24 (0.1–0.41)	0.8–1.0	-	-
*Mesocricetus*	0.65 (0.32–1.06)	2.5–2.7	1.81 (1.04–2.61)	-
*M. newtoni*/*M. brandti*	0.37 (0.16–0.64)	1.7–1.8	-	-
Cricetini	1.9 (1.21–2.7)	5.8–7.4	6.36 (4.83–8.01)	8.45 (5.64–11.39)^e^
*Cricetulus*/*Nothocricetulus *+ *Allocricetulus* + *Cricetus*	1.63 (1.04–2.39)	-	5.61 (4.43–6.99)	7.78 (4.93–10.7)
*Nothocricetulus*/*Allocricetulus + Cricetus*	1.43 (0.9–2.1)	-	4.46 (3.41–5.88)	6.56 (3.61–9.52)
*Cricetus*/*Allocricetulus*	1.17 (0.69–1.75)	-	2.65 (1.60–4.00)	5.46 (2.67–8.35)
*Cricetulus*	0.48 (0.21–0.82)	2.9–3.1	1.06 (0.50–1.64)	4.02 (2.43–6.51)

The age ranges of Neumann et al. [12] follow their implementations of different clocks and calibration points. For the other studies, the median (this study) or mean [7, 9] ages and 95% HPD ranges are given

^a^excludes *A. curtatus*, *Cansumys canus*, *C. sokolovi*, *M. raddei*, *T. collina*, *U. lama*

^b^excludes *A. curtatus*, *Ca. canus*, *C. sokolovi*, *T. collina*, *U. kamensis*, *U. lama*

^c^excludes *Ca. canus*, *M. newtoni*, *M. raddei*, *P. campbelli*, *T. collina*

^d^excludes *A. curtatus*, *M. brandti*, *M. newtoni*, *M. raddei*, *P. sungorus*

^e^for the comparison, the age of Cricetini excluding *Ca. canus* is taken

All other above-mentioned studies built their analyses directly or indirectly upon the results of Steppan et al. [101]. Lebedev et al. [7] took the age inferred by Steppan et al. [101] for Cricetidae (18.7–19.6 Ma, 95% CI 16.6–21.1 Ma) as basis of their root age prior. This was followed by Jiang et al. [8], although their root corresponds to the split of Arvicolinae and Cricetinae (outgroup *Lagurus lagurus*), which is considerably younger (see [16]). Pan et al. [9] utilized three calibration points, based on the inferred ages and calibration points of Lebedev et al. [7]. Consequently, all the applied calibration strategies depend on each other, which can be problematic (see, e.g., [108]). In this case, the age of Cricetidae estimated again in later studies, turned out to be noticeably younger (14.6 Ma in [16]). The general problem in dating the origin of crown cricetines lies in the difficulty of finding reliable calibration points, as the phylogenetic relationships between fossil and extant taxa are often unclear (see, e.g., †*Cricetus lophidens*).

Lebedev et al. [7] employed three calibration points within Cricetinae considering the age of *Mesocricetus*, the split of *Cricetus* and *Allocricetulus*, and the age of *Cricetulus*. The first one constrained the origin of *Mesocricetus* to be older than 4.9 Ma, based on †*Mesocricetus primitivus* from Silata [109]. However, in this study, †*M. primitivus* is shown to likely not belong to *Mesocricetus* (Fig. 2). The next oldest fossil attributed to the genus is *Mesocricetus auratus* from Dursunlu [106, 110], which is considerably younger, dated to the latest early Pleistocene (ca. 0.99–0.78 Ma) [111]. Their second calibration point applied a minimum age of 2.7 Ma to the split of *Cricetus* and *Allocricetulus*, supported by three fossils [7]. Firstly, they mention †*Cricetus runtonensis* from Rebielice-Królewskie, Poland [98], as correlated biostratigraphically to the Mammal Neogene zone 16 (MN16). This material was revised by Pradel [112], who distinguished between older and younger fossils (RK-1A and B). †*Cricetus runtonensis* belongs to the younger material and was suggested to be Upper Biharian in age (ca. 0.9–0.5 Ma) (see also [113]). The older material (MN16) was described as *Cricetus* sp. 3. However, this classification is doubtful, given the age of the publication, as several Neogene ‘*Cricetus’* taxa were later transferred to different fossil genera, such as †*Pseudocricetus* and †*Apocricetus* [32, 61]. The same issue applies to the second *Cricetus* fossil cited by Lebedev et al. [7] from Selim-Dzhevar. According to the NOW database [78], this record is originally based on a catalog by Borissiak and Belyaeva (1948), which is unavailable to us. Due to the age of the reference, the occurrence requires revision in light of the subsequent transfer of Neogene ‘*Cricetus’* taxa. The third fossil cited by Lebedev et al. [7] is *Allocricetulus* from Kiikbay (MN16). In the referenced publication, it was named “*Allocricetus eversmani*” [114]. Although we cannot rule out the possibility that *“Allocricetus”* and “*eversmani*” are simply typos and that the occurrence refers, in fact, to *Allocricetulus eversmanni*, the large gap to the otherwise oldest known *Allocricetulus* fossils raises additional doubts about this classification. These next-oldest known *Allocricetulus* fossils are from the middle Pleistocene of Italy, Romania and Russia (ca. 0.424–0.125 Ma) [68, 115–117]. The third calibration point of Lebedev et al. [7], constrained the divergence of *Cricetulus* to be older than 2.7 Ma based on *Cricetulus* cf. *barabensis* (Transbaikalia, MN16) [118]. In the latest description of the Baikalian fauna, this Pliocene fossil was reassigned to *Cricetulus* sp. [119]. This attribution is problematic, given that *Cricetulus *sensu lato is polyphyletic (including *Urocricetus* and *Nothocricetulus*). The oldest fossil attributed to *C. barabensis*, known to us, is magnetostratigraphically dated to the early Pleistocene (ca. 2.595–2.14 Ma) [55, 120]. In summary, all three calibration points proposed by Lebedev et al. [7], the latter two of which were also used by Pan et al. [9], seem to be too old. Although the discrepancy is smaller in the third one, their use may have led to an overestimation of divergence dates.

As our estimated divergence ages are substantially younger, they do not produce long ghost lineages, as in previous studies (see, e.g., for *Phodopus* in [12]). However, in some cases, the oldest fossils, attributed to extant species, predate the respective divergence ages, as illustrated by the stratigraphic ranges depicted in Fig. 2 (see also Additional file 2 [121–371] for the oldest fossil occurrences of each taxon). While the difference is small in most cases, it is considerable in the case of *Cricetulus*, for which our estimated divergence date is evidently too young, as there are several fossils from the early Pleistocene of China and Russia, attributed to *C. barabensis* [55, 195, 316, 372–374] and to *C. longicaudatus* [99, 316]. The reason of the underestimation in this case and in general may partly be that not all extant species (e.g., *Cricetulus sokolovi*) or fossil species (e.g., taxa potentially assignable to *Cricetus*, see [69]) could be included in the analysis. Accounting for the whole known stratigraphic ranges (see [375]) of both fossil and extant species might likewise result in older estimates. Another possible explanation for older divergence ages within crown hamsters is related to the relatively low support of their monophyly (Fig. 2, clade I, PP = 0.52). If additional, older taxa are included in the crown cricetine clade, the inferred divergence ages will also turn out to be older. In summary, most previous divergence estimates are not fully independent and therefore tend to confirm each other. While many of these estimates appear too old compared with the fossil record, our own estimates seem to be too young in the case of *Cricetulus*.

### Biogeographical history of cricetines

A recent study on carnivores demonstrated the importance of including fossil taxa when inferring the geographical origin of extant clades [376], an approach that we applied here to hamsters for the first time. While the most probable area of origin of all cricetines is Central to Eastern Europe (Fig. 3), the uncertainty is high in the oldest estimates (Additional file 1: Fig. S3). Nonetheless, the estimate fits with the oldest members of the group, previous suggestions and reconstructions, as well as proposed ancestors of the Cricetinae [1, 88, 98, 158, 238].

**Fig.5:**
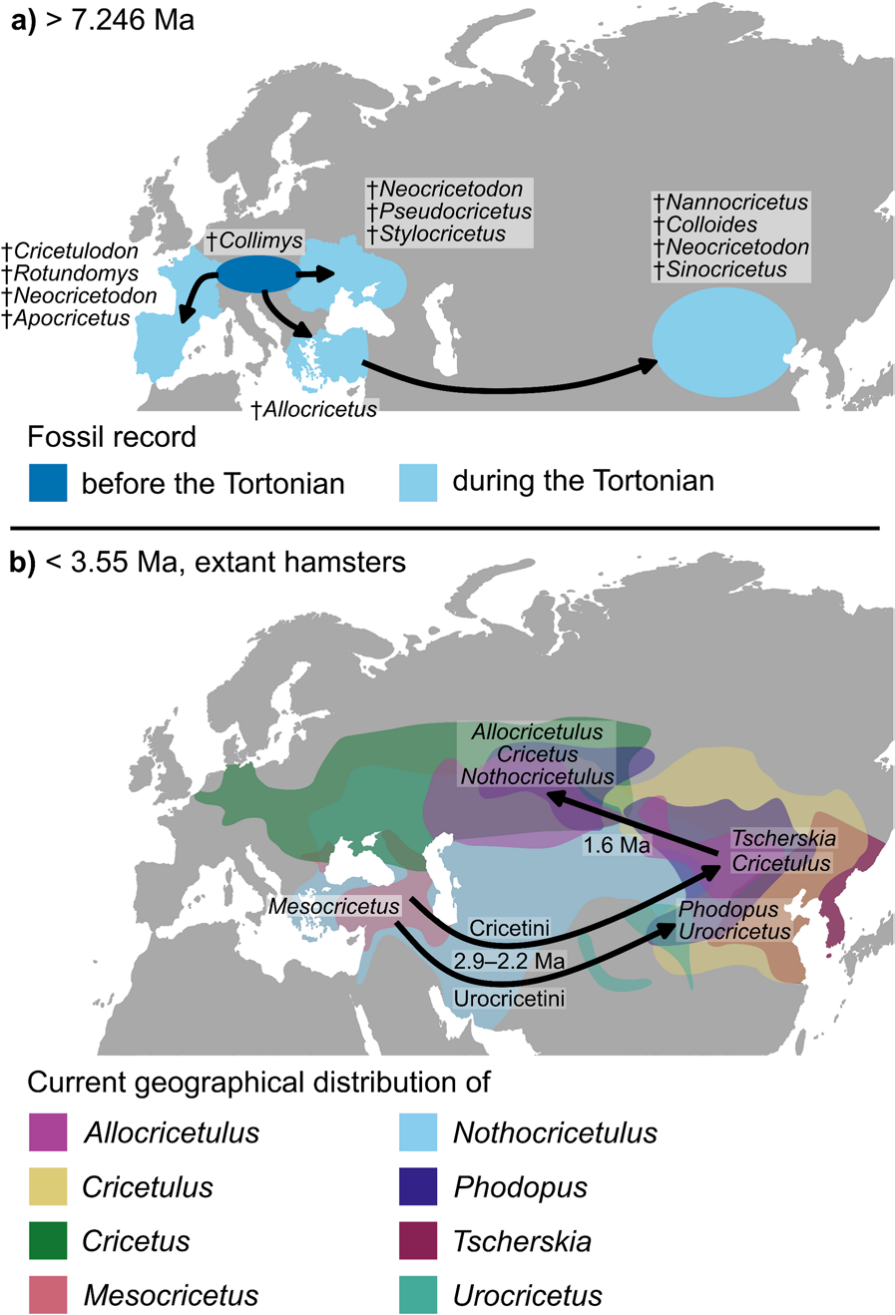
Main dispersals **a** of fossil cricetines before and during the Tortonian, and **b** of extant cricetines. Arrows in **a** represent one or multiple dispersal waves during the Tortonian, colored areas and selected genera represent the fossil record. Arrows in **b** represent main dispersal events, colored areas show today’s geographical distribution of extant genera with their inferred geographical origin indicated

From this Central to Eastern Europe area, the cricetines dispersed into South-Western Europe several times. The first arrivals were †*Cricetulodon* and †*Rotundomys* around 12.2 Ma. Apart from †Alloc*ricetus ehiki* and extant hamsters, the latest species to invade South-Western Europe was †*Neocricetodon polonicus* around 5.5 Ma. All other dispersals into this area (†*Apocricetus*, †*Cricetulodon meini* and †*C. lucentensis*, †*Neocricetodon*, †*Tragomys*) likely occurred in several waves during the Tortonian (11.63–7.246 Ma) (Fig. 5a). Globally, the Tortonian, is characterized by increasingly open environments [377]. The precise timing of habitat opening in Central and Western Europe has however long been debated (see [378]). According to climate reconstructions, the spread of open vegetation occurred later in time, especially in Central Europe [379]. The Iberian Peninsula, however, already had a more open and drier landscape during the Tortonian [380], which coincides with the diversification of several hamster taxa in the region. Given that today’s hamsters are mostly found in open environments, such as steppes, grasslands or semi-deserts [2, 3], a connection between the expansion of such habitats and hamster diversification and dispersal seems plausible and has been suggested before [12].

While the first expansions into Eastern Central Asia of †*Colloides* and †*Nannocricetus primitivus* (ca. 12.2–11.4 Ma) remain elusive, due to the high uncertainty in reconstructed ancestral geographic ranges (Additional file 1: Fig. S3), the following two waves of dispersal eastwards (during 10.2 to 6.7 Ma), involving several †*Neocricetodon* and †*Allocricetus* taxa (Fig. 2, clades C and D), appear to be connected to prior dispersals into South-Western Europe and South-Western Asia (Fig. 5a). At the same time (ca. 10–7 Ma), palaeobotanical evidence indicates increasingly open vegetation with spreading grasslands in Greece and Anatolia [381]. Additionally, an expanding open savannah connecting Europe and Eastern Asia from 9 to 6 Ma has been proposed [382], potentially facilitating eastward dispersal of cricetines (but see [383]). Several groups of large mammals are known to have expanded towards the East during the Tortonian as well [384–386]. Some of these taxa, including e.g., Carnivora or Proboscidea, distributed additionally into Africa [387], in contrast to the hamsters, that remained limited to Europe and Asia.

Around 8.9 to 8 Ma, the lineage leading to the extant hamsters moved toward the area of Eastern Europe and South-Western Asia, which is also inferred as the most likely area for the origination of extant hamsters. Given the limited fossil and extant diversity of hamsters documented from Iraq to Pakistan, we focus primarily on the region around the Eastern Mediterranean and the Caucasus, close to the distribution of *Mesocricetus* today. Two main limitations affect this estimation: firstly, the uncertainty of the reconstructed ancestral ranges themselves, especially at the node leading to extant hamsters (Additional file 1: Fig. S3), and secondly, the phylogenetic uncertainty. Therefore, the nodes leading to all potential sister groups of extant hamsters, i.e., clades G, H, †*Cricetus lophidens* and †Alloc*ricetus ehiki*, should also be considered. Here, either an origin in South-Eastern Europe to South-Western Asia is reconstructed, or a broader range including this area. Apart from †Apoc*ricetus*, which is restricted to South-Western Europe, the region around the Eastern Mediterranean and the Caucasus also fits the localities of the respective fossil taxa, that are closest to potential ancestors of crown hamsters. †*Hypsocricetus*, †*Cricetus lophidens*, and †*Mesocricetus primitivus* were all described from Greece [83, 87]. †*Moldavimus* is distributed in the area of Central and Eastern Europe but close to the Eastern Mediterranean and Caucasus, at the Black Sea in Southern Moldova, Southern Ukraine, and Eastern Romania [32, 244, 280, 388]. Lastly, †A. *ehiki* is more widely distributed but also around the Black Sea and the Eastern Mediterranean [180, 276]. Except for †A. *ehiki*, all these other taxa diverged in the late Miocene, when steppe habitats were present specifically along the Black and Caspian Seas [389].

Consequently, the Mesocricetini remained in this region, after the first split within the crown hamsters around 3.55 Ma, while the two lineages, leading to the Urocricetini and Cricetini, subsequently invaded East Central Asia (Fig. 5b). The lineage leading to the Urocricetini expanded around 2.93 Ma, whereas the lineage leading to the Cricetini dispersed between 2.93 Ma and 2.18 Ma. This interval was characterized by increased aridification in Central Asia [389], consistent with the current distribution of *Phodopus* in deserts and semi-deserts [369, 390]. According to our analysis, all taxa within the Urocricetini, i.e., *Phodopus* and *Urocricetus*, subsequently diverged in East Central Asia, confirming previous suggestions of an eastern origin [7]. Following the latter lineage, all genera within the Cricetini diverged between ca.1.9 and 1.17 Ma, during a strong cooling event that ended after 1.2 Ma, when the so-called Mid-Pleistocene transition began [391]. During that time, glacial cycles shifted from 41 to 100 kyr periods, which resulted in intensified glaciations and additional remarkable climatic changes [392].

Our analysis places the origin of *Tscherskia* in Eastern Asia, although a European origin of *Tscherskia* was recently proposed based on the transfer of European ‘†*Cricetinus*’ species to *Tscherskia* [54], a re-assignment not supported by our analysis. Around 1.63 Ma, *Cricetulus* split from the remaining Cricetini, remaining in Eastern Asia, while the other lineage dispersed into Russia and Kazakhstan (Fig. 5b), where it subsequently diverged into *Nothocricetulus, Allocricetulus*, and *Cricetus* (1.43–1.17 Ma). This coincided with an intensified opening of landscapes during the early Pleistocene in this region [393]. Afterwards, *Nothocricetulus, Allocricetulus*, and *Cricetus* spread independently into Europe and, in the case of *Nothocricetulus*, also into Eastern Asia, at a time when “prototypes of periglacial steppes” were found generally in northern Eurasia during cold phases [389]. The abundance of fossil species (or subspecies) attributed to *Cricetus* in Europe further suggests subsequent diversification of this genus there (see [90]).

## Conclusions

Due to the partly highly similar dental morphology of different fossil hamster taxa, delimitation of species and genera can be difficult. Our phylogenetic hypothesis allows us to propose several taxonomic changes, most importantly regarding the fossil species that were originally described as †*Cricetinus*. According to our results, †*Cricetulus mesolophidos* belongs to †*Neocricetodon*, and †*C. europaeus* to †*Pseudocricetus*, while †*C*. *koufosi* forms a clade with several species described as †*Allocricetus*. In line with previous studies, we confirm †*Allocricetus* as non-monophyletic and call for erecting a new genus for the above-mentioned clade that includes †*C*. *koufosi*. Several additional fossil species do not belong to their originally described genus according to our analysis, namely, †*Apocricetus darderi*, †*Mesocricetus primitivus* and †*Cricetus lophidens*. Furthermore, we confirm that †*Cricetulodon* is non-monophyletic. The type species of the third non-monophyletic genus in our analysis, †*Nannocricetus*, is considered a rogue taxon in our analysis. After accounting for taxonomic changes, we confirm eleven (of 14) genera that are represented by more than one species in our dataset as monophyletic. For the first time, a total-evidence dating analysis is performed on a hamster dataset that includes extant and extinct taxa. We provide estimations of divergence dates, using tip-dating, independently of previous studies and without relying on a priori assumptions about the placement of fossil taxa in the tree. In this way, we infer an origin of crown hamsters in the Pliocene rather than in the late/middle Miocene. Probabilistic modeling indicates that the ancestor of the subfamily was located in Central to Eastern Europe, with subsequent dispersal events into Western Europe and Eastern Central Asia. The crown hamsters are inferred to have originated in the region surrounding the Black Sea and the Eastern Mediterranean.

## Methods

### Assembling the combined morphological and molecular data matrix

#### Taxonomic sampling

The morphological dataset used here expands upon Dirnberger et al. ([11], suppl. mat. S2). The latter focused on the earliest, diverse European Neogene Cricetinae, including 41 species in seven genera, plus the outgroup †*Eucricetodon wangae* Li, Meng & Wang, 2016, as coded in López-Antoñanzas and Peláez-Campomanes [39]. A relatively distant outgroup was chosen due to remaining uncertainty regarding the relationships between cricetines and closely related fossil cricetids, such as †*Democricetodon* and microtoid cricetids [1, 238]. To assess the effect of outgroup choice, we repeated the total-evidence dating (TED) analysis using †*Democricetodon franconicus* as outgroup and additionally performed the analysis without an outgroup. The resulting topologies were virtually identical, differing only in weakly supported relationships, and divergence time estimates showed only minor differences (< 0.2–0.5 Ma) with largely overlapping 95% HPD intervals. As the analysis without an outgroup showed convergence difficulties, we retained †*E. wangae* as outgroup. Additional analyses are provided in Additional file 1 (see also Additional file 1: Figs. S5 and S6). Compared to the preceding analysis [11], we considerably expanded the taxon sampling by including: (1) non-European species, (2) species of monospecific genera and (3) species ranging from the Pleistocene to the present. The full diversity of both extinct and extant Cricetinae comprises around 109 species across 28 genera (Additional file 2). For 82 species across 24 genera (75% of the total diversity), adequate material and sufficiently detailed descriptions were available to us, allowing their inclusion in this analysis, thus doubling the previous taxonomic coverage. Among these 82 species, 69 are extinct (77% of all extinct species), and 13 are extant (68%). For details of all included taxa, including stratigraphic ranges, examined material, or references used for morphological data, as well as for general taxonomic information of the group, see Additional file 2.

#### Morphological characters

The morphological matrix, edited in Mesquite v.3.81 [394], includes discrete morphological and morphometric characters of all six molars, following López-Antoñanzas and Peláez-Campomanes [39], and Dirnberger et al. [11], as well as their dental terminology. For the most important characters discussed here, see Fig. 1. The morphological data for the newly incorporated taxa were obtained from published descriptions and images, a micro-CT scan provided by the Natural History Museum in London, and direct examination of specimens housed in the following collections and institutes: Bavarian State Collection for Palaeontology and Geology, Munich; Bavarian State Collection for Zoology, Munich; Faculty of Sciences, University Claude Bernard, Lyon; Institute of Evolutionary Science of Montpellier, University of Montpellier; National Museum of Natural History, Paris (for details, see Additional file 2). The character composition of the matrix was slightly revised, compared to Dirnberger et al. [11]. We removed invariable characters, that had remained from the original analysis [39], and added two characters: the presence or absence of a direct connection between the posterior spur of the lingual anteroconid and the metalophulid in the m1 (character 86) and the presence or absence of a small extra ridge between the middle of the mesolophid and the metaconid in the m3 (character 88). Additionally, we changed and recoded the character states of the labial posteroloph in the M1 (character 87, in Dirnberger et al. [11]: character 26). Furthermore, a contingent coding scheme was used, as it avoids biases introduced by multistate coding and, under Bayesian inference, the negative impact introduced by inapplicable characters [395]. In case of intraspecific variability, the condition observed in the type material was prioritized. When variability was present among specimens from the type locality, the character state present in the majority of specimens was coded. If no clear majority could be identified, the character was treated as polymorphic. The complete morphological matrix with character descriptions is provided as Additional file 3.

#### Molecular data

For all 13 extant species, molecular data were obtained from GenBank [396]. Following previous molecular phylogenetic analyses (e.g., [7, 12]), the data include sequences of six genes: Cytb, VWF, IRBP, GHR, RAG1, and BRCA1. These genes show high taxonomic coverage for hamsters and have been shown to resemble phylogenetic relationships from nuclear genome-wide and mitogenomic analyses [9, 97]. For details of the sources and GenBank accession numbers, see Additional file 4 [396–399]. The sequences were aligned in AliView v.1.28 [400] with MUSCLE [401], manually trimmed, and concatenated in SeaView v.5.1 [402], resulting in a total of 5,828 base pairs. The alignment is available in Additional file 5. The molecular data were then combined with the morphological ones in Mesquite, resulting in a total-evidence matrix of 5916 characters.

#### Removal of rogue taxa

Before obtaining the final phylogenies, a set of eleven so-called rogue (‘wildcard’) taxa [403] was identified to be removed from the taxon set. These taxa are unstable in their position in the tree set, and their removal therefore increases the overall posterior probabilities of the clades of the consensus tree [30, 404]. For information on the removed taxa and their identification, see Additional file 1 (see also Additional file 1: Figs. S7 and S8). The downstream analyses were then run with the reduced taxon set of 71 cricetine taxa plus the outgroup, †*Eucricetodon wangae*.

### Tip-dated relaxed-clock analysis

Relaxed-clock Bayesian inference analyses were performed using the developer version of MrBayes (future MrBayes 3.2.8 release) compiled from source code available at (https://github.com/NBISweden/MrBayes) [24, 405]. Analyses were conducted in the DELLA cluster at Princeton University.

#### Character evolution model

For the evolution of morphological characters, the Mkv model [26] was used, which also corrects the ascertainment bias of including only variables characters. In our dataset, autapomorphies are present to avoid a sampling bias that could affect dating [406]. Rate variation across sites was considered by allowing for four discrete rate categories that approximate a gamma distribution [27], with shape (alpha) sampled from an exponential distribution with mean = 1.0.

The best fitting partitioning scheme and substitution models for the molecular data were found with the Partitionfinder2 algorithm [407] implemented in IQTREE [408] under the ‘greedy’ algorithm, selected with the corrected Bayesian information criterion (BIC). The resulting partitions and the respective substitution models (HKY + G and GTR + G [27, 409, 410]) can be found in the MrBayes input files in Additional file 6: Files S1.

#### Clock models

To relax the assumption of a constant clock rate, several relaxed clock models have been proposed, which allow relative rates to change between lineages. In the development version of MrBayes, four different ways to model relaxed clock rates are implemented [405]. One of them (TK02) assumes an autocorrelation of rates between branches [411], whereas the other three allow the relative rate of each branch to be drawn independently, either from independent log-normal distributions (ILN), from independent gamma (IGR) distributions [412] or from gamma distributions with variance proportional to the branch length [413]. The latter model is called the white noise model (WN) in the latest MrBayes version. However, it has been named ‘IGR model’ in previous versions of MrBayes (< v.3.2.7a) [405]. The choice of clock model has been shown to impact estimated divergence times and evolutionary rates for morphological and total evidence datasets, thus emphasizing the importance of finding the best fitting model to the respective dataset [414, 415].

Here, we calculated the best fit clock model to the data using stepping-stone sampling with 30 steps and a burn-in of 5 steps for 200 million generations [416]. As stepping-stone analyses struggled to reach convergence for the present dataset, we calculated Bayes factors using marginal likelihoods from both stepping-stone sampling and harmonic means (Additional file 6: Files S1). Although the latter is less accurate than the former [416], the runs reached stationarity and convergence. The results from both approaches show that the TK02 model had the worst fit. Among the other three models, the IGR and WN models are better supported than the ILN model. As the WN model had substantially better convergence diagnostics than IGR, we report here the results of the WN clock in the main text (but see Additional file 1: Fig. S4 for the resulting phylogeny from the IGR clock).

For the prior for the base clock rate, we used a log-normal distribution, with the mean calculated by dividing the median tree length of a preceding non-clock analysis by the median root age prior (6.12/37.1 = 0.165, in natural log scale: −1.802) (following [28]). For a broad standard deviation, the exponent of the mean (*e*^0.165^ = 1.179) was chosen (following e.g., [29]). The morphological and molecular clocks are unlinked (but linked among molecular partitions), which has been shown to be generally better fitting than a single linked clock [414, 417–419].

#### Tree model and calibrations

The fossilized birth–death (FBD) tree model was used, which allows the inclusion of fossil taxa as tips while modeling the process of fossil recovery and incomplete species sampling over time [420–422]. We kept the default, flat prior distributions for the relative extinction rate (turnover) and relative fossilization rate (Beta(1.0,1.0)) but used an exponential distribution with mean = 1.0 for the net diversification rate. Following our sampling of extant species, which was aimed at maximizing the included diversity, we used the ‘diversity’ sampling strategy since accounting for different sampling strategies can have an impact on the estimated diversification rates [423]. The extant sampling probability (rho) is fixed to 13/19 = 0.68.

As prior for the root age, an offset exponential distribution was set with a minimum age of 33 Ma (the minimal age of the oldest included fossil species, †*Eucricetodon wangae*) and a mean age of 41.2 Ma [39]). All other calibration points were based on fossil tip-dating [19, 424], constraining the ages of the fossil tips to uniform distributions that follow the stratigraphic ranges of the fossil species and uncertainties in the dates of localities (for more information about the specific ranges, see Additional file 2). Assigning range calibrations instead of points avoids possible errors in the estimated divergence times [425]. Lastly, the ingroup was constrained to be monophyletic to help with correct rooting and reaching convergence.

#### MCMC and output processing

The analyses were run with four independent Metropolis-Coupled Markov chain Monte Carlo runs (MCMCMC), with four chains each, sampling every 1,000 generations for a total of 120 million generations, and a burn-in of 25%. Convergence of runs was checked by the potential scale-reduction factor (PSRF ≈ 1) [426], the average standard deviation of split frequencies (ASDSF < 0.01) [427], the estimated sample size (ESS > 200), and visually in Tracer v.1.7.2 [428]. The resulting maximum clade compatibility consensus trees (‘allcompat’) were visualized in R with the treeio v.1.30.0, ggtree v.3.14.0 and deeptime v.2.1.0 packages [429–432]. The input and output files of the stepping-stone sampling and the WN and IGR analyses are available in Additional file 6: Files S1 and in the online Supplementary data [433].

### Estimation of ancestral biogeography

To reconstruct ancestral geographical ranges, each taxon was assigned to one or more of five large geographical areas (Fig. 3), based on their fossil occurrences and/or extant biogeography (following [3, 434]). The ancestral ranges were inferred, using the maximum-likelihood framework of the R package BioGeoBEARS v.1.1.3 (BioGeography with Bayesian (and likelihood) Evolutionary Analysis with R Scripts) [40, 41, 435], which allows also for testing which model of geographical range evolution fits the data best. All compared models allow for anagenetic range expansion and contraction but differ in the way cladogenesis events are modeled. BayArea [436] (here, likelihood version: BAYAREALIKE) assumes inheritance of the geographical range identically to both daughter lineages, whereas the dispersal-extinction-cladogenesis (DEC) model [437] and the dispersal‐vicariance analysis [438] (here, likelihood version: DIVALIKE) take slightly varying events of sympatry and vicariance into account (summarized in [439]). Additionally, founder-event speciation (jump dispersals) can be included in all three models (model + J), resulting in six different models to be tested. For all models, ranges of non-adjacent geographical areas and dispersal between such areas were excluded.

The best fitting model is the DEC + J model, based on the corrected Akaike information criterion (Akaike weight: 0.9, see Additional file 1). The resulting most likely ancestral ranges are shown in the main text (Fig. 3). As the implementation of jump dispersals is under discussion [440, 441], the results of the remaining models are shown in Additional file 1: Figures S9–S18, including the most likely estimates, as well as pie charts to show uncertainties. The R code used for the analysis, and the input and output files are available in Additional file 7.

## Supplementary Information

Additional file 1: Text document (.pdf) with descriptions of additional analyses and their results regarding rogue taxa, outgroups, and biogeographic range evolution models, including Figures S1–18. Fig. S1 – Total-evidence WN phylogeny including the outgroup †*Eucricetodon wangae*. Fig. S2. – Preliminary morphological IGR phylogeny including rogue taxa. Fig. S3 – Biogeographical history reconstructed under the DEC + J model with ancestral ranges shown as pie-charts. Fig. S4 – Total-evidence IGR phylogeny after exclusion of the rogue taxa. Fig. S5 – Total-evidence IGR phylogeny without an outgroup. Fig. S6 – Total-evidence IGR phylogeny with †*Democricetodon franconicus* Fahlbusch, 1966 as outgroup. Fig. S7 – RoguePlot showing the potential positions of †*Nannocricetus mongolicus* in the tree. Fig. S8 – RoguePlot showing the potential positions of †*Sinocricetus zdanksyi* in the tree. Figs. S9–S18 – Biogeographical histories reconstructed under the DEC, DIVALIKE and BAYAREALIKE models with and without jump dispersals, with ancestral ranges shown as most likely estimates and pie-charts.

Additional file 2: Text document (.pdf) with additional information about the studied taxa, including geographical distrubution, systematic information, stratigraphic range, oberserved material, and anatomical references.

Additional file 3: Nexus file (.nex) containing the morphological matrix used for the phylogenetic analyses, including descriptions of the characters and states.

Additional file 4: Text document (.pdf) containing the GenBank accession numbers of sequences used for the phylogenetic analyses, including explanations and references for chosen sequences.

Additional file 5: Nexus file (.nex) containing the concatenated alignments of the six gene sequences of the 13 extant cricetine species included in this study.

Additional file 6: Files S1–2. Files S1 – Input and output files of the IGR and WN total-evidence Bayesian inference analyses and the stepping-stone sampling, including all necessary information to reproduce the analyses, as well as resulting Bayes factors using marginal likelihoods from stepping-stone sampling and harmonic means. Files S2 – Input and output files of the additional phylogenetic and RoguePlot analyses described in Additional file 1, including all necessary information to reproduce the analyses.

Additional file 7: Output files of the BioGeoBEARS analyses, as well as the R script and the input files, that are necessary to reproduce the analyses.

## Data Availability

All data generated or analyzed during this study are included in this published article and its Additional files and in the Supplementary data freely available at Harvard’s Dataverse online repository, 10.7910/DVN/CDV3HJ [433].

## Abbreviations

ASDSF: Average standard deviation of split frequencies
BAYAREALIKE: Likelihood version of BayArea
BIC: Bayesian information criterion
BioGeoBEARS: Biogeography with Bayesian (and likelihood) evolutionary analysis with R scripts
DEC: Dispersal-extinction-cladogenesis
DIVALIKE: Likelihood version of the dispersal‐vicariance analysis
ESS: Estimated sample size
FBD: Fossilized birth–death tree model
+ G: Including the discrete-gamma model for among site rate variation
GTR: Generalized time reversible substitution model
HKY: Hasegawa-Kishino-Yano substitution model
IGR: Independent gamma rate uncorrelated clock model
ILN: Independent lognormal uncorrelated clock model
+ J: Including jump dispersals
m1, m2, m3: First, second, and third lower molars
M1, M2, M3: First, second, and third upper molars
MCMCMC: Metropolis-Coupled Markov chain Monte Carlo
Mkv: Markov model conditioned on variable characters
MN: Mammal Neogene zone
PSRF: Potential scale-reduction factor
TK02: Continuous autocorrelated clock model
WN: White noise uncorrelated clock model
