# Supplementary material for: Total-evidence phylogeny reveals recent crown group radiation and biogeographical history of hamsters
Source: BMC Biol. 2026 Apr 9;24:117. doi: 10.1186/s12915-026-02581-z (PMC13173810; doi:10.1186/s12915-026-02581-z)
Supplement: Supplementary file 1 — Additional file 1: Text document (.pdf) with descriptions of additional analyses and their results regarding rogue taxa, outgroups, and biogeographic range evolution models, including Figures S1–18. Fig. S1 – Total-evidence WN phylogeny including the outgroup †Eucricetodon wangae. Fig. S2. – Preliminary morphological IGR phylogeny including rogue taxa. Fig. S3 – Biogeographical history reconstructed under the DEC + J model with ancestral ranges shown as pie-charts. Fig. S4 – Total-evidence IGR phylogeny after exclusion of the rogue taxa. Fig. S5 – Total-evidence IGR phylogeny without an outgroup. Fig. S6 – Total-evidence IGR phylogeny with †Democricetodon franconicus Fahlbusch, 1966 as outgroup. Fig. S7 – RoguePlot showing the potential positions of †Nannocricetus mongolicus in the tree. Fig. S8 – RoguePlot showing the potential positions of †Sinocricetus zdanksyi in the tree. Figs. S9–S18 – Biogeographical histories reconstructed under the DEC, DIVALIKE and BAYAREALIKE models with and without jump dispersals, with ancestral ranges shown as most likely estimates and pie-charts. [file 12915_2026_2581_MOESM1_ESM.pdf]

Additional file 1 for

Total-evidence phylogeny reveals recent crown group radiation and biogeographical history of hamsters

Moritz Dirnberger[1], Pablo Peláez-Campomanes[2], Tiago R. Simões[3], Raquel López-Antoñanzas[1, 2]

[1]Institut des Sciences de l'Évolution de Montpellier, Université de Montpellier, CNRS, IRD, 34095 Montpellier, France

[2]Departamento de Paleobiología, Museo Nacional de Ciencias Naturales-CSIC, Madrid, Spain

[3]Department of Ecology and Evolutionary Biology, Princeton University, Briger Hall, Princeton-NJ, 08544, USA.

Additional analyses and results regarding rogue taxa, outgroups, and biogeographic range evolution models

The input and output files of all following analyses can be found in Additional files 6 and 7.

Additional phylogenetic trees and depictions

Figure S1 shows the phylogeny of Cricetinae resulting from the total-evidence WN Bayesian inference analysis (see Fig. 2), including the outgroup *Eucricetodon wangae*. Figure S2 shows the phylogeny resulting from the preliminary IGR analysis based on only the morphological data of the complete taxon set. Figure S3 shows the reconstructed biogeographical history based on the DEC+J model with ancestral ranges shown as pie-charts. Figure S4 shows the total-evidence phylogeny under the IGR clock after removing the rogue taxa. Figures S5 and S6 show the total-evidence IGR analyses without an outgroup and with *Democricetodon franconicus* Fahlbusch, 1966 as outgroup. Figures S7 and S8 show the RogePlots regarding *Nannocricetus mongolicus* and *Sinocricetus zdanksyi*. Figures

S9–S18 show the reconstructed biogeographical histories under the different models available in BioGeoBEARS with ancestral ranges shown as most likely estimates and pie-charts.

Rogue taxa identification and positions

To identify taxa that are unstable in the reconstructed phylogenies, a preliminary phylogenetic analysis was run on the morphological data (Additional file 3). The analysis was done in MrBayes 3.2.7a [24] using the Cyber-Infrastructure for Phylogenetic Research (CIPRES) Science Gateway v.3.3 [25]. We used the Mkv model [26] with among character rate heterogeneity under a gamma distribution [27]. Following the previous analysis of older Neogene taxa [11], an uncorrelated relaxed-clock model was chosen, the IGR (independent gamma rate) model. For the prior of the base clock rate, a log-normal distribution was chosen, with the mean calculated by dividing the median tree length of a preceding non-clock analysis by the median of the root age prior (6.02/37.1 = 0.162, in natural log scale: -1.82) (following e.g. [28]). For a broad standard deviation, the exponent of the mean ($e^{0.162}$ = 1.176) was chosen (following [29]). The remaining settings for the tree model and constraints are the same as described in the manuscript. The analyses were run with four independent Metropolis-Coupled Markov chain Monte Carlo runs (MCMCMC) with six chains, sampling every 1,000 of 50,000,000 generations, and a burn-in of 30%.

The resulting posterior tree sample was used to identify the rogue taxa with the R package Rogue v.2.1.6 [30, 31] (for the used commands, see [11], suppl. mat. S4). The analysis resulted in eleven identified rogue taxa, all of them extinct: *Allocricetus anterolophidens*, *Apocricetus plinii*, *Cricetulodon hartenbergeri*, *Cricetulus gritzai*, *Cricetulus beremendensis*, *Nannocricetus mongolicus*, *Neocricetodon occidentalis*, *Neocricetodon progressus*, *Pseudocricetus polgardiensis*, *Sinocricetus progressus*, *Sinocricetus zdanskyi*. The position of these taxa in the tree is shown in Figure S2.

Most of the species are scored comprehensively, with non-applicable and unknown character states (- and ?) not higher than for the remaining taxa (6.2 % in total). Therefore, the identifications as rogue taxa probably follow from a combination of character states that does not allow a clear positioning in the tree. Exceptions are hereby *Allocricetus anterolophidens* and *Cricetulus gritzai*. For both of which the percentage of not-scored characters is higher (11.4 and 18.2 %) due to the limited material (only a single M3 for both, see [32]), which probably contributed to their status as rogue taxa in this analysis.

As *Sinocricetus zdanskyi* and *Nannocricetus mongolicus* are both the type species of their respective genera, we ran an additional analysis in the CIPRES Science Gateway v.3.3 [25] excluding all above-mentioned rogue taxa, except for *S. zdanskyi* and *N. mongolicus*. The analysis is based on the IGR analysis excluding all rogue taxa (Fig. S4), with the IGR model chosen based on the Bayes factors comparing the different clock models mentioned in the main text. The only difference refers to the prior of the base clock rate, determined by a preceding non-clock analysis: lognorm(-1.50756,1.24789).

To assess the instability of the two taxa, we used the create.rogue.plot command of the R package RoguePlots v.1.0-1. [33] based on a subsample of 10,000 trees from the posterior tree sample. The different positions of *Nannocricetus mongolicus* and *Sinocricetus zdanksyi* are indicated in Figures S7 and S8 respectively. The highest probability (0.26) of attachment of *N. mongolicus* is found as sister to *Nannocricetus qiui* within clade D. Apart from clade D there are low probabilities (1%–10%) of branching within clade E and in or near clade I. *Sinocricetus zdanksyi* is positioned beween *Cricetulodon meini* + *C. lucentenisis* and clades F–I with the highest probability (0.25), whereas also other positions, within and towards *Cricetulodon meini* + *C. lucentenisis* and clade D have probabilities > 1%. In general, the position of both taxa, *S. zdanskyi* and *N. mongolicus*, is relatively unstable, as expected based on their identification as rogue taxa.

Testing of different outgroup taxa

To test for the effect of different outgroups, we repeated the above-mentioned IGR analysis without all rogue taxa and without an outgroup, which is suggested to follow better the model assumptions of the FBD [34, 35]. The prior of the root age takes the age of the ingroup determined by the main analysis and by Dirnberger et al. [11] as a soft maximum and the age of the oldest ingroup fossil taxon *Collimys transversus* (see [36]) as minimum age. Additionally, we tested *Democricetodon franconicus* Fahlbusch, 1966 as an outgroup taxon, which is supposed to be closer to the ingroup [1]. Morphological data, as well as the age of the taxon, and the divergence date used for the prior of the root age, are taken from the original publication and previous phylogenetic analyses [1, 37–39].

Both analyses showed problems reaching convergence, similar to the analysis under the IGR clock model with *Eucricetodon wangae* as outgroup, especially the reconstruction without an outgroup (ESS slightly below 100 for two parameters). In comparison to the IGR tree with *E. wangae* (Fig. S4), the obtained topologies are congruent, except for the position of *Allocricetus bursae* in the tree without an outgroup (Fig. S5) and the relationship of the clade including *Collimys* and the clade including *Rotundomys* in the tree with *Democricetodon franconicus* (Fig. S6). The divergence dates of the tree without an outgroup are widely overlapping in their 95% highest posterior density (HPD) ranges and only slightly younger medians with changes of < 0.5 Ma. Exceptions are here a few of the oldest splits, in which differences are larger (up to around 1 Ma). The divergence dates in the tree including *D. franconicus* are nearly identical or slightly younger compared to the tree with *E. wangae* with differences in the median age of < 0.2 Ma and largely overlapping 95% HPD ranges.

Eucricetodon wangae is relatively distant from the ingroup phylogenetically and chronologically (see e.g., [1]) but this ensures that the taxon is outside of the ingroup. An analysis including *Democricetodon* and the oldest members of Cricetinae included in this study (e.g., *Collimys*) has not been performed to date, and *Democricetodon* could therefore

be potentially included in our ingroup. Given this issue and the problems of the analysis without an outgroup in reaching convergence, *Eucricetodon wangae* is chosen as the outgroup of the main analysis.

Model comparison of different biogeography evolution models

Resulting from the BioGeoBEARS analysis [40, 41] described in the main text based on the WN tree excluding all rogue taxa and the outgroup taxon *Eucricetodon wangae*, the different models can be compared using the corrected Akaike information criterion (see Tab. S1). The results of the less fitting models and the estimated ancestral states depicted as pie-charts of the DEC+J model can be seen in Figures S3, S9–S18. For the DEC+J model, the rate of anagenetic range expansion is d = 0.1032, the rate of range contraction is e = 0.0616, and the rate of jump dispersal is j = 0.048. The rates for the remaining models can be seen in the respective Figures S9–S18.

Table S1. Log-likelihood (LnL), corrected Akaike information criterion (AICc, best in bold) and Akaike weights (AICc_wt) of each of the six tested model for ancestral biogeographical range estimations.

Models	LnL	AICc	AICc_wt
DEC	-173.3	350.8	0.055
DEC+J	-169.4	**345.3**	0.9
DIVALIKE	-186.6	377.4	9.50E-08
DIVALIKE+J	-173.8	354	0.012
BAYAREALIKE	-191.2	386.5	9.90E-10
BAYAREALIKE+J	-172.6	351.6	0.037

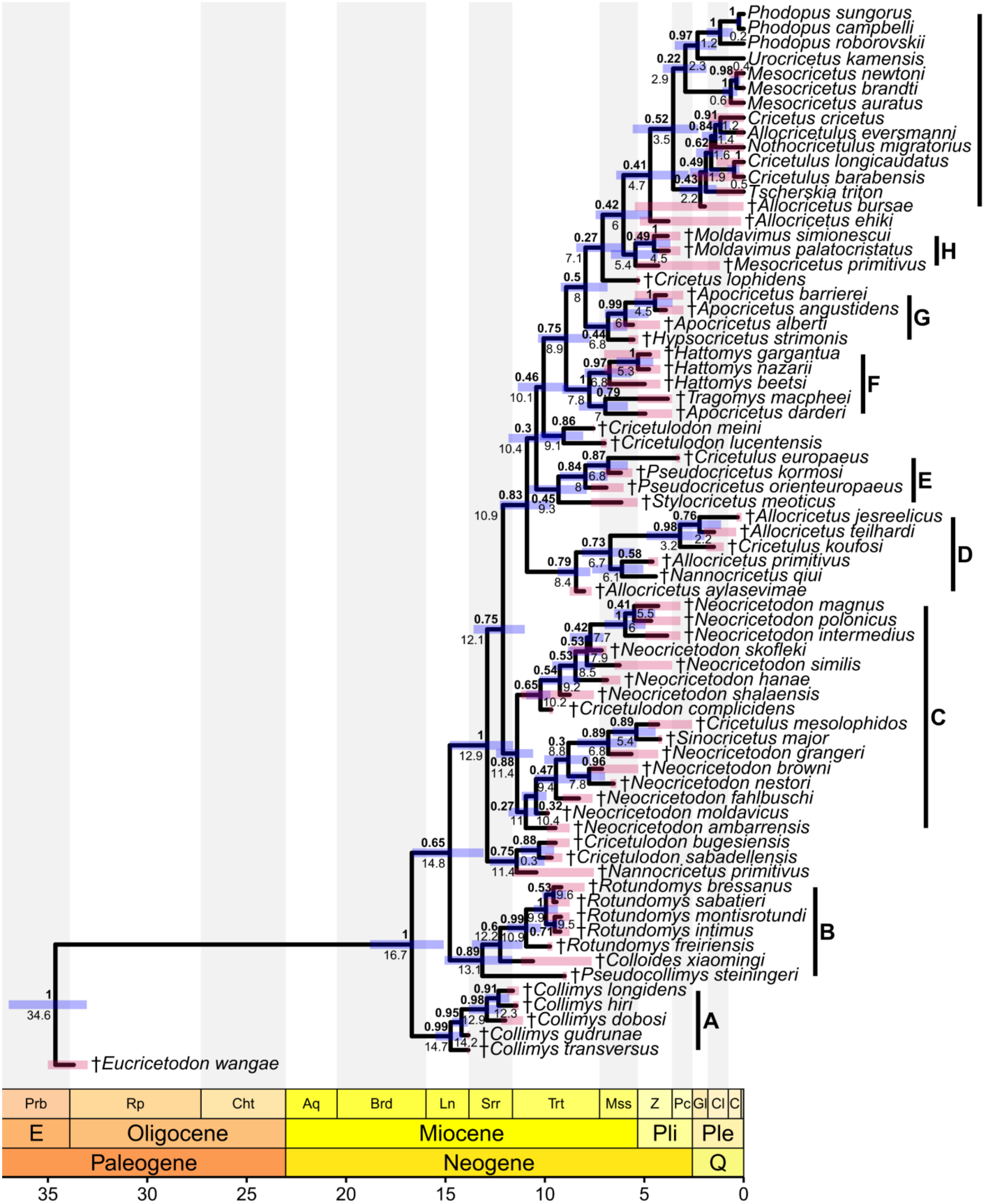

Figure S1. Tip-calibrated relaxed-clock WN Bayesian inference analysis based on the reduced taxon set and the combined data (Fig. 2), including the outgroup. Posterior probabilities (in bold) and median ages of clades are indicated at respective nodes, blue node bars indicate the 95% highest posterior density for divergence times, red tip bars indicate the stratigraphic range of the taxa. The scale axis is in Ma, the chronostratigraphic chart follows Cohen et al. [23].

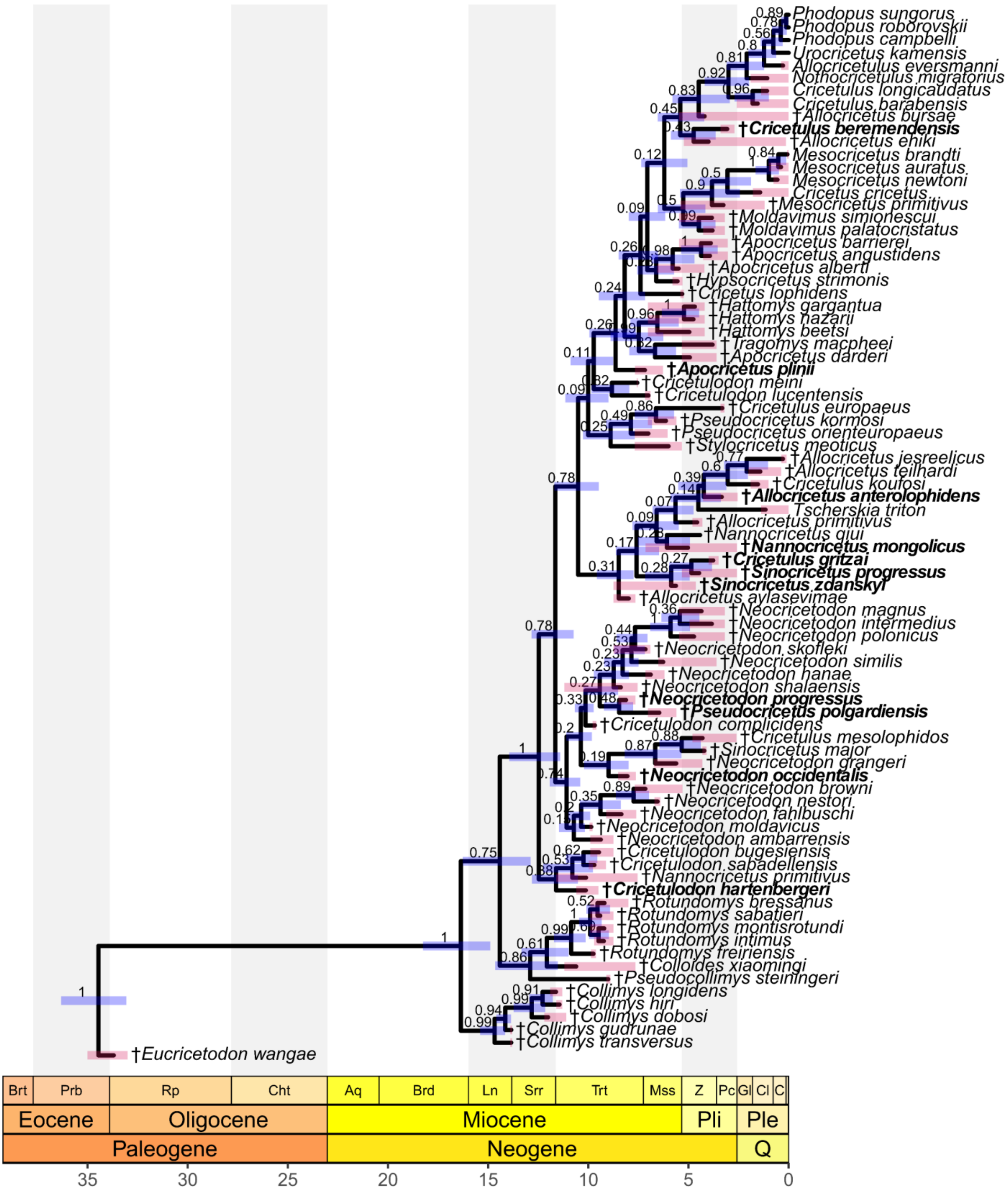

Figure S2. Maximum clade compatibility tree of the preliminary tip-calibrated relaxed-clock IGR Bayesian inference analysis based on the complete taxon set and only the morphological data. Identified rogue taxa shown in bold. Posterior probabilities of clades are indicated at respective nodes, blue node bars indicate the 95% highest posterior density for divergence times, red tip bars indicate the stratigraphic range of the respective taxon. The scale axis is in Ma, the chronostratigraphic chart follows Cohen et al. [23].

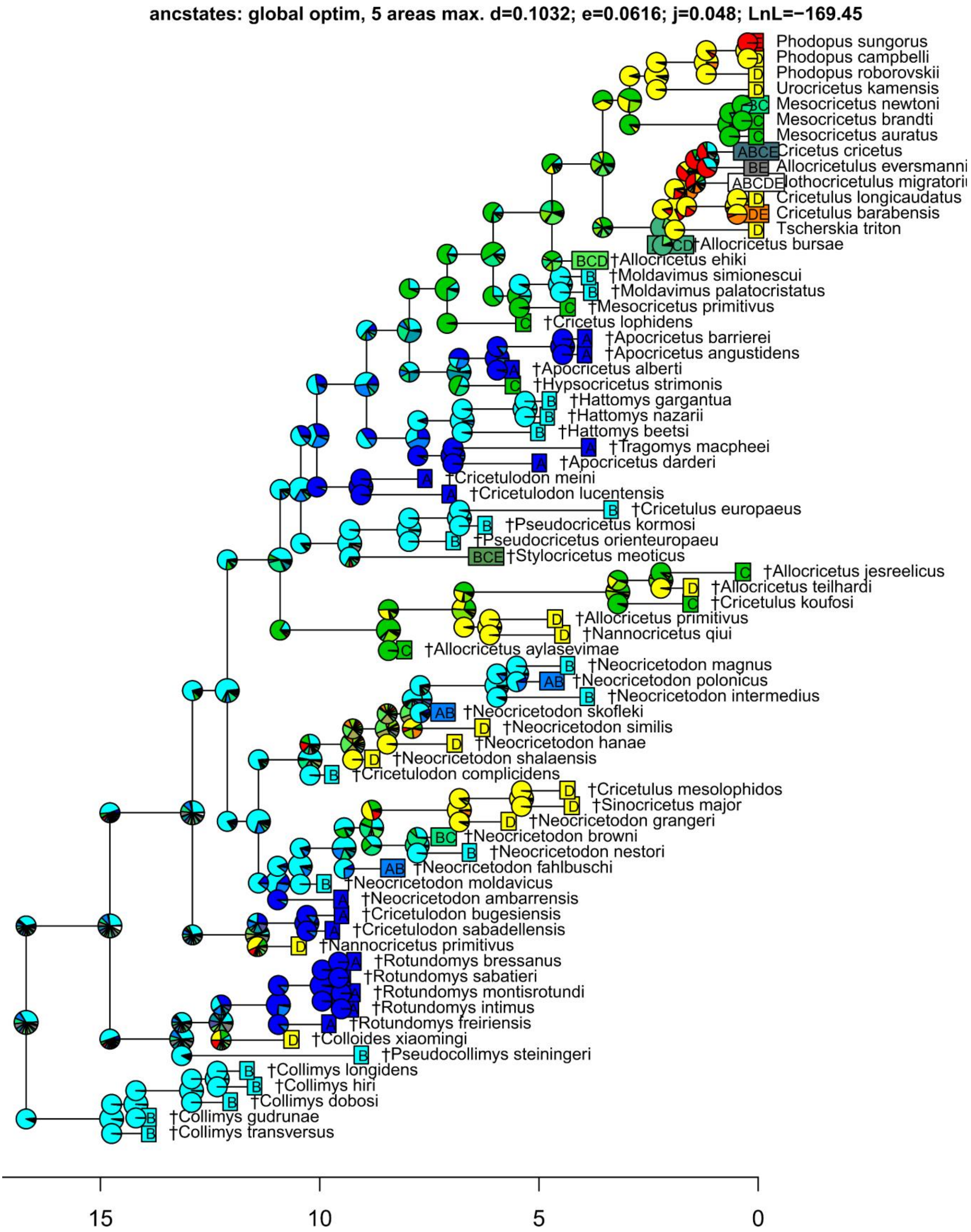

Figure S3. Reconstruction of the biogeographical history of Cricetinae based on the DEC+J model. Tip ranges and estimated ancestral ranges as pie-charts are represented by colors and letters. For visual representation of the areas and most likely estimates, see Figure 3 in the main text. **A**: South Western Europe, **B**: Central and Eastern Europe, **C**: South Eastern Europe, South Western Asia, **D**: Eastern Central Asia, **E**: Western Central Asia. Scale axis in Ma.

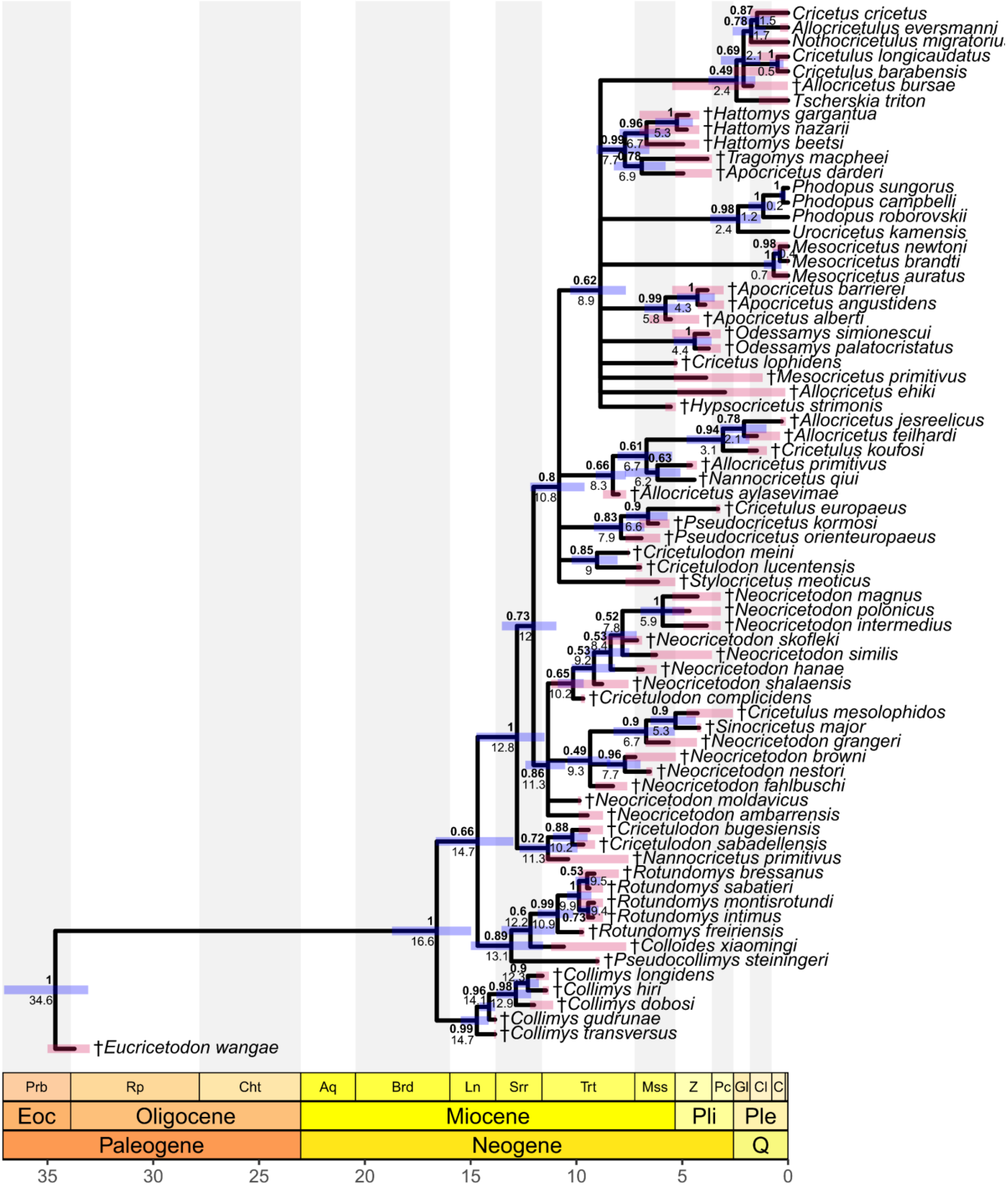

Figure S4. Maximum clade compatibility tree of the tip-calibrated relaxed-clock IGR Bayesian inference analysis based on the reduced taxon set and the combined data. Posterior probabilities (in bold) and median ages of clades are indicated at respective nodes, blue node bars indicate the 95% highest posterior density for divergence times, red tip bars indicate the stratigraphic range of the taxa. The scale axis is in Ma, the chronostratigraphic chart follows Cohen et al. [23].

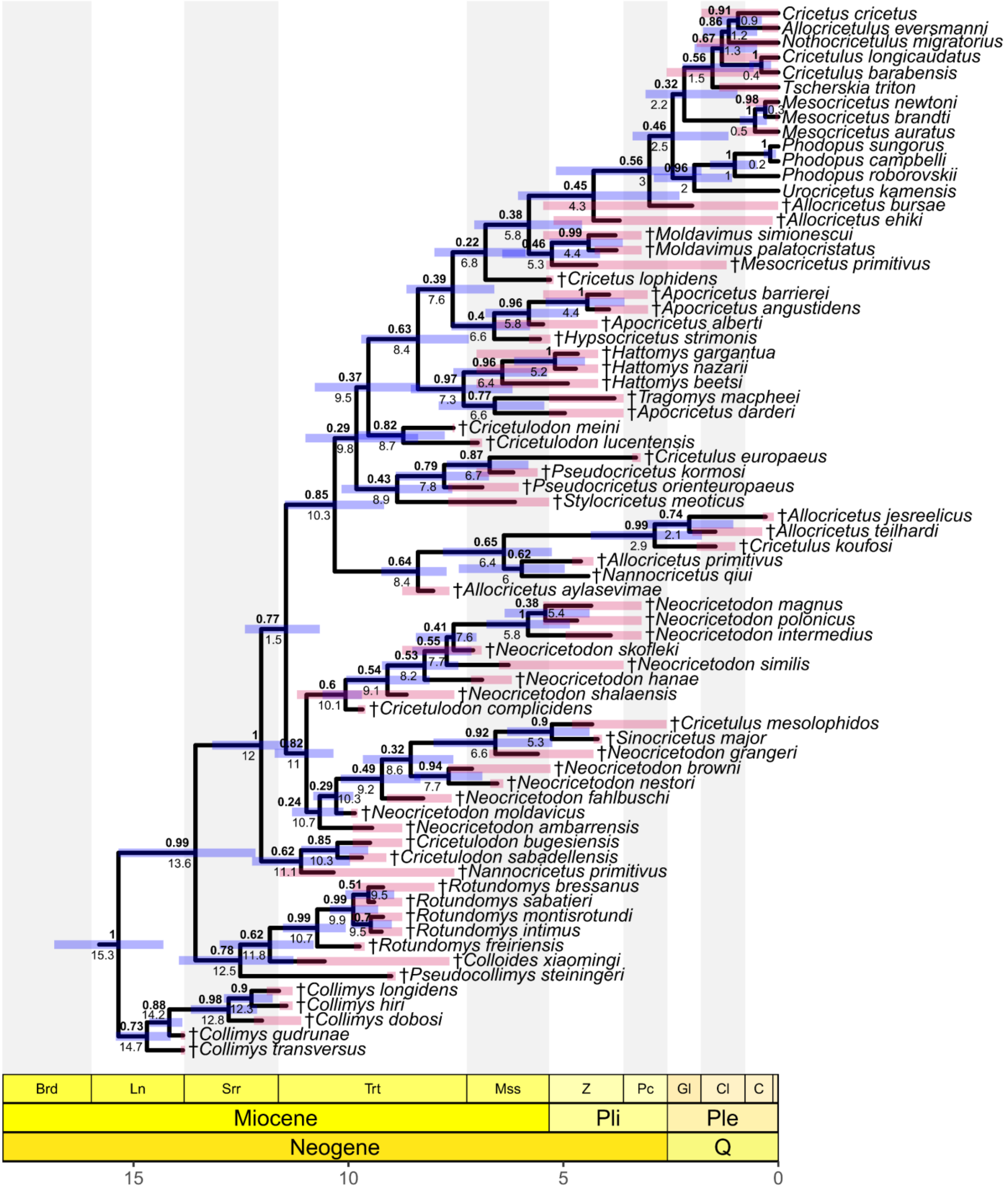

Figure S5. Maximum clade compatibility tree of the total-evidence relaxed-clock IGR Bayesian inference analysis based on the taxon set without rogues, and without an outgroup. Posterior probabilities (in bold) and median ages of clades are indicated at respective nodes, blue node bars indicate the 95% highest posterior density for divergence times, red tip bars indicate the stratigraphic range of the taxa. The scale axis is in Ma, the chronostratigraphic chart follows Cohen et al. [23].

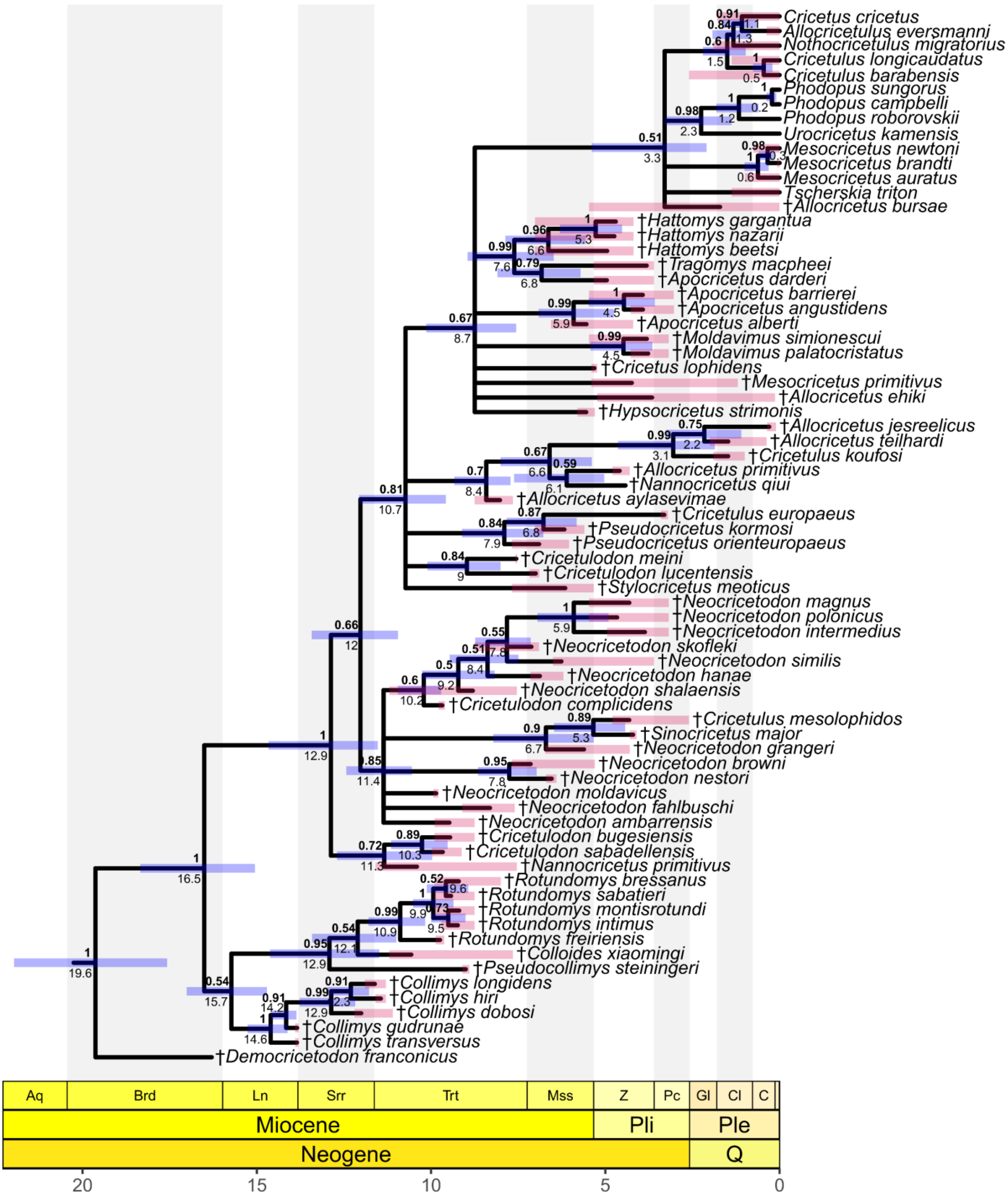

Figure S6. Maximum clade compatibility tree of the total-evidence relaxed-clock IGR Bayesian inference analysis based on the taxon set without rogues, and *Democricetodon franconicus* as outgroup. Posterior probabilities (in bold) and median ages of clades are indicated at respective nodes, blue node bars indicate the 95% highest posterior density for divergence times, red tip bars indicate the stratigraphic range of the taxa. The scale axis is in Ma, the chronostratigraphic chart follows Cohen et al. [23].

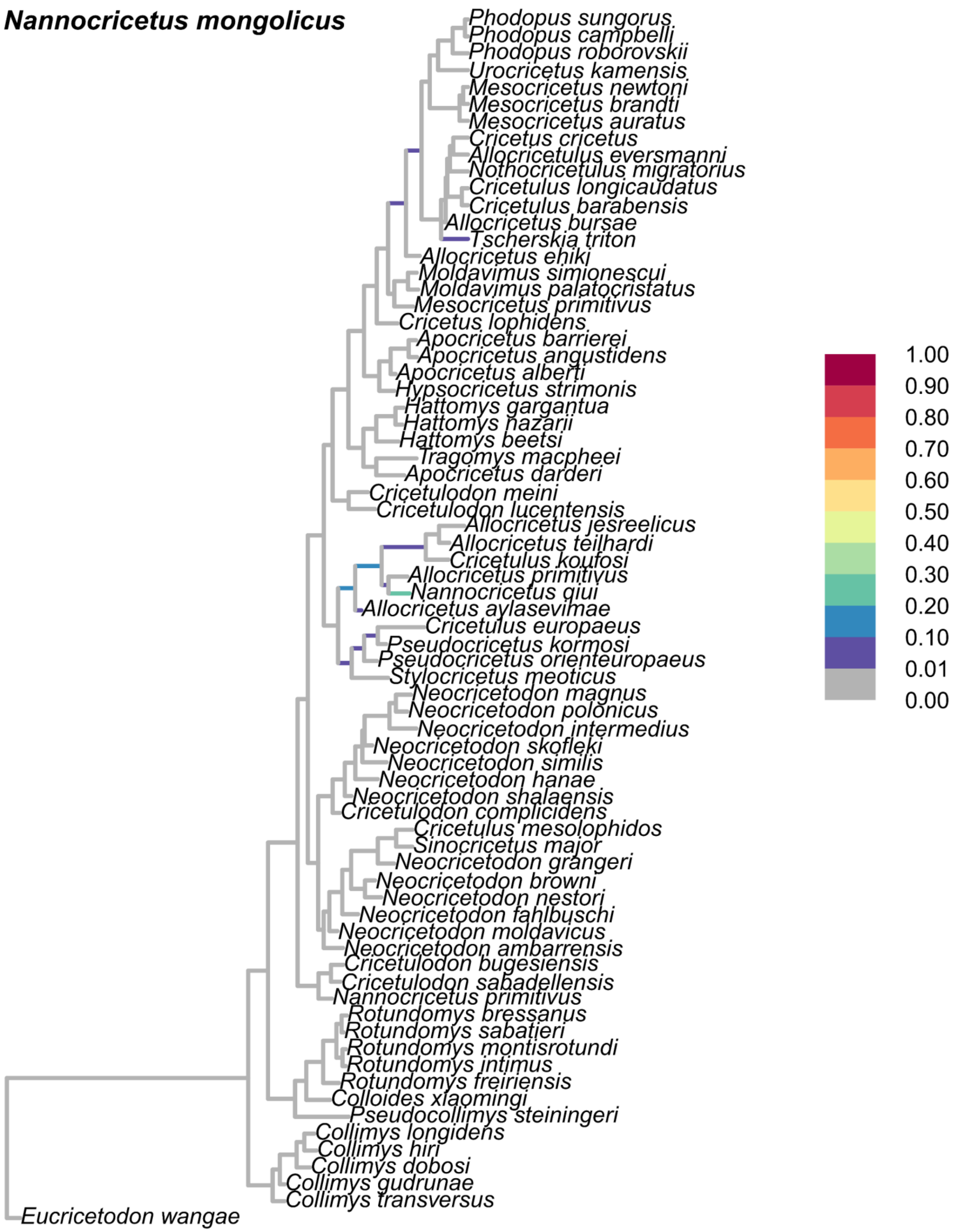

Figure S7. Maximum clade compatibility tree of the total-evidence relaxed-clock IGR Bayesian inference analysis based on the taxon set without rogues, but including *Nannocricetus mongolicus* and *Sinocricetus zdanskyi*. Colors of the branches reflect the probability of attachment of *Nannocricetus mongolicus* to the respective branch.

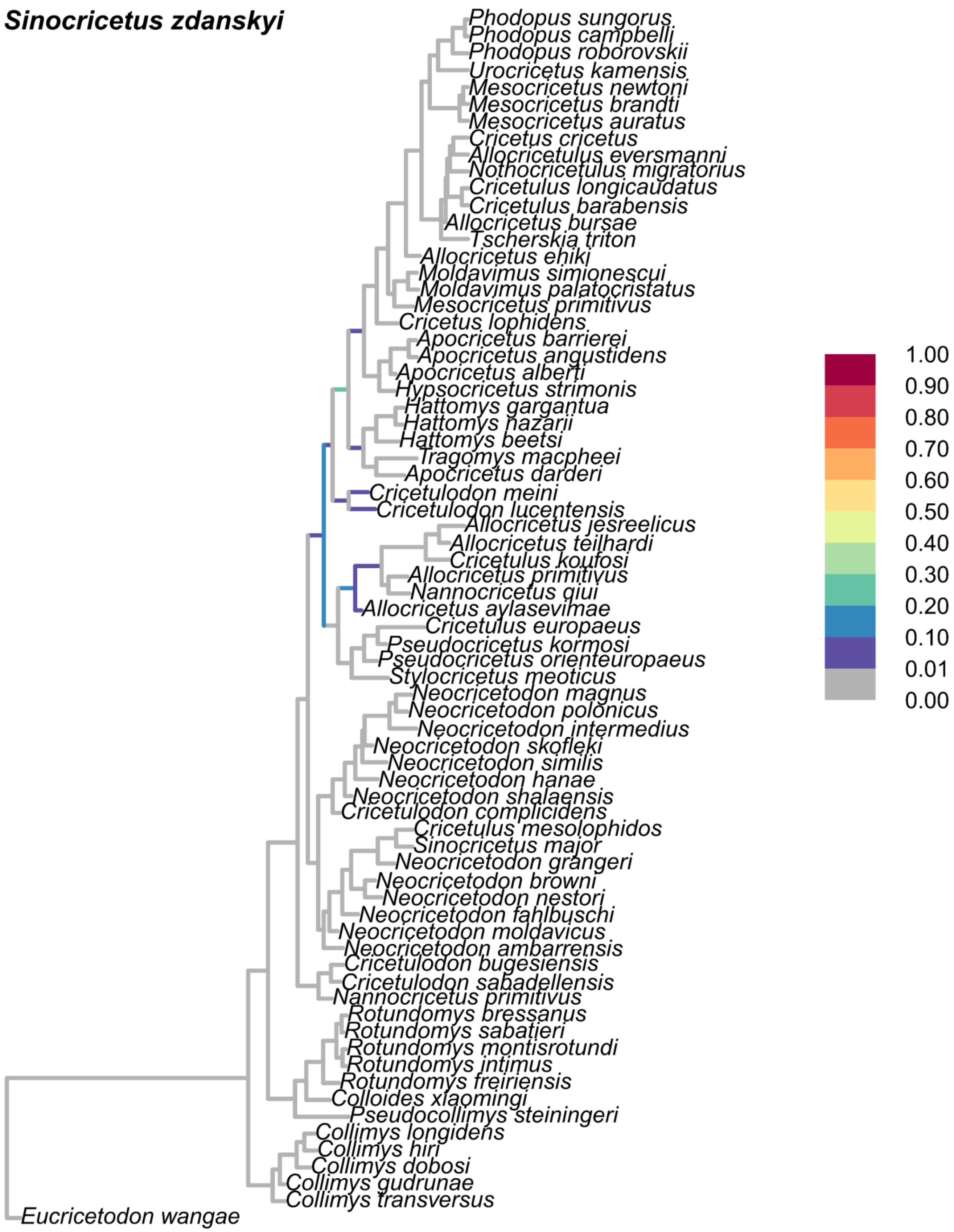

Figure S8. Maximum clade compatibility tree of the total-evidence relaxed-clock IGR Bayesian inference analysis based on the taxon set without rogues, but including *Nannocricetus mongolicus* and *Sinocricetus zdanskyi*. Colors of the branches reflect the probability of attachment of *Sinocricetus zdanskyi* to the respective branch.

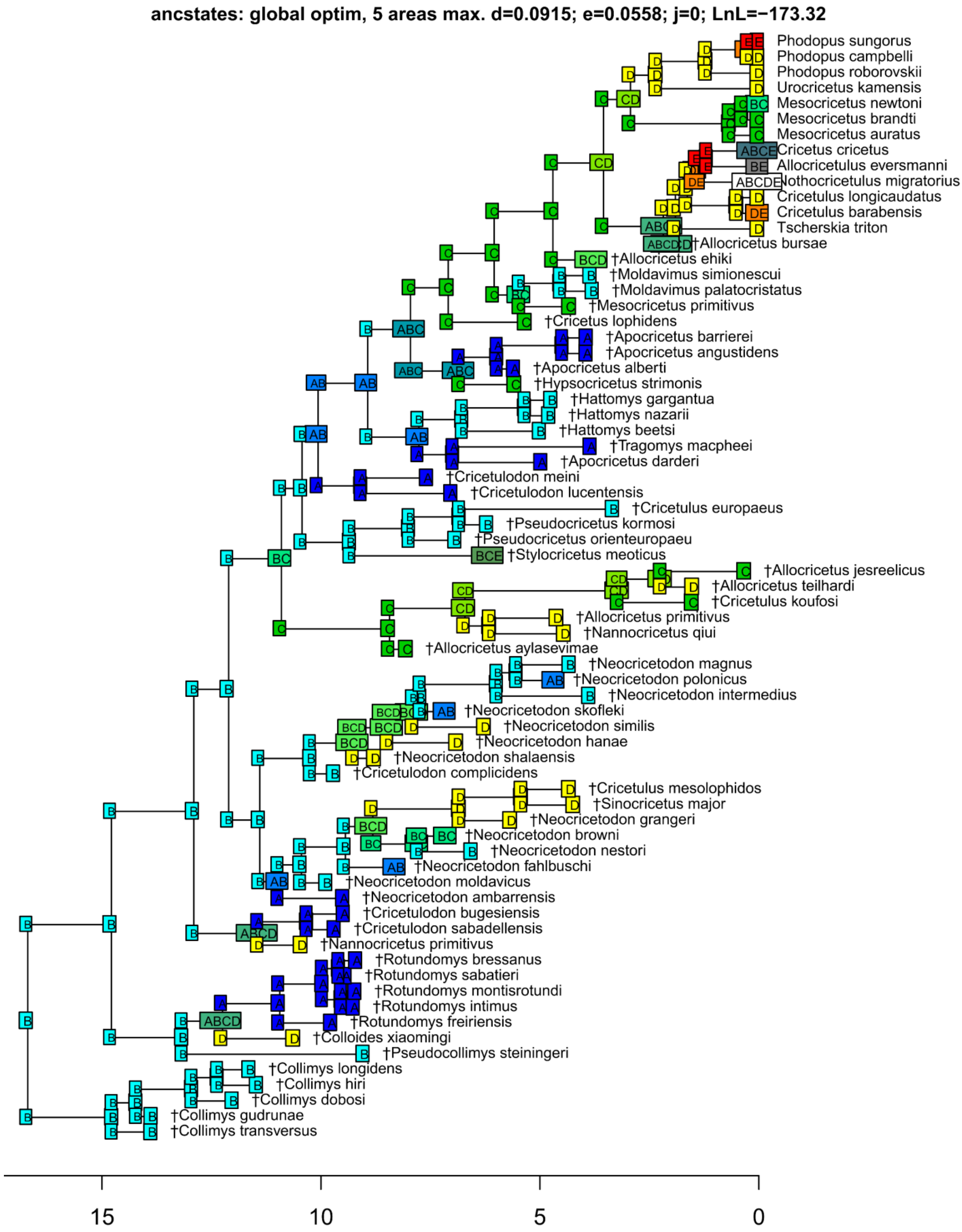

Figure S9. Reconstruction of the biogeographical history of Cricetinae based on the DEC model. Ancestral ranges are shown as most likely estimates. See Figure S3 for explanations of tip ranges and estimated ancestral ranges. Scale axis in Ma.

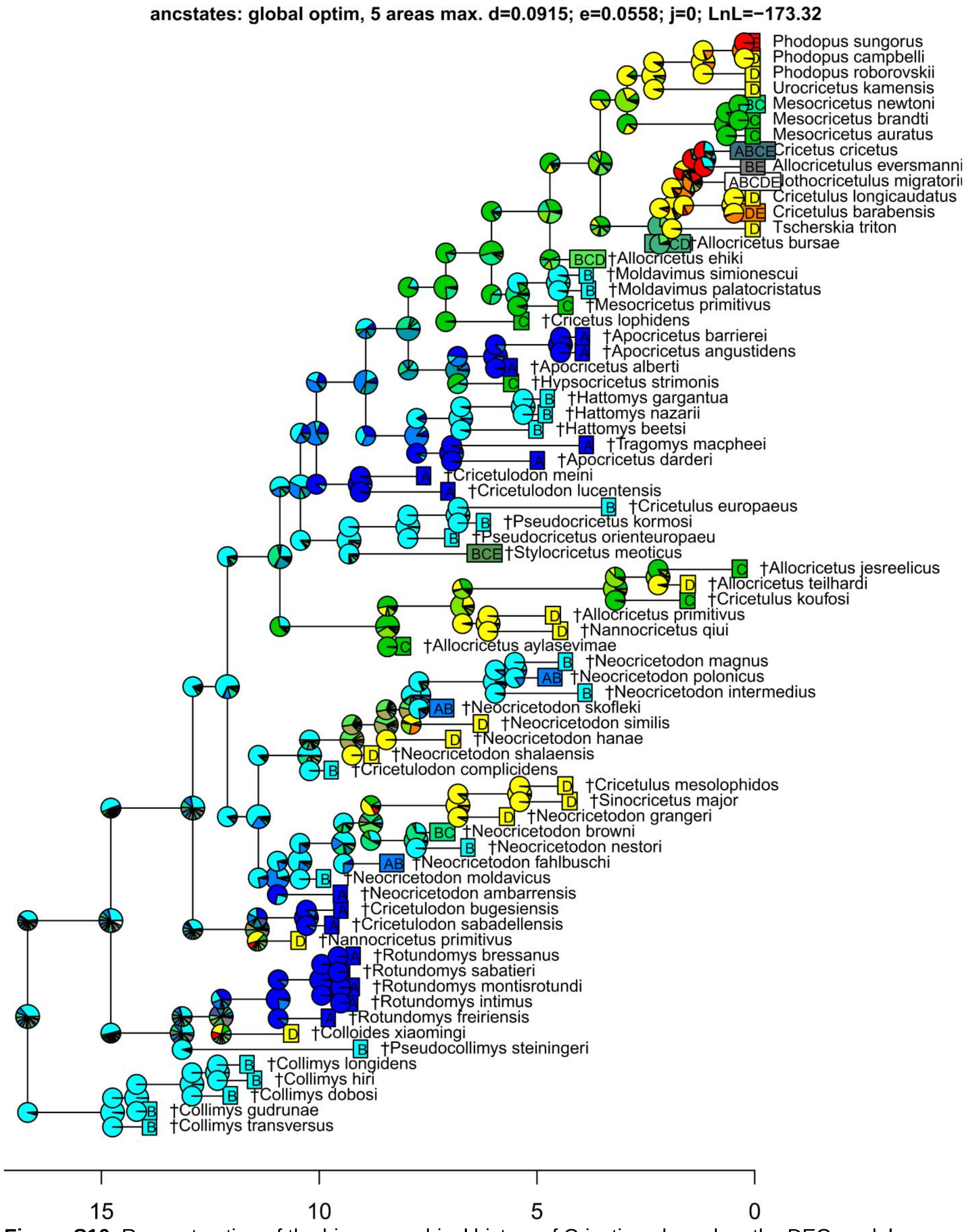

Figure S10. Reconstruction of the biogeographical history of Cricetinae based on the DEC model. Ancestral ranges are shown as pie-charts. See Figure S3 for explanations of tip ranges and estimated ancestral ranges. Scale axis in Ma.

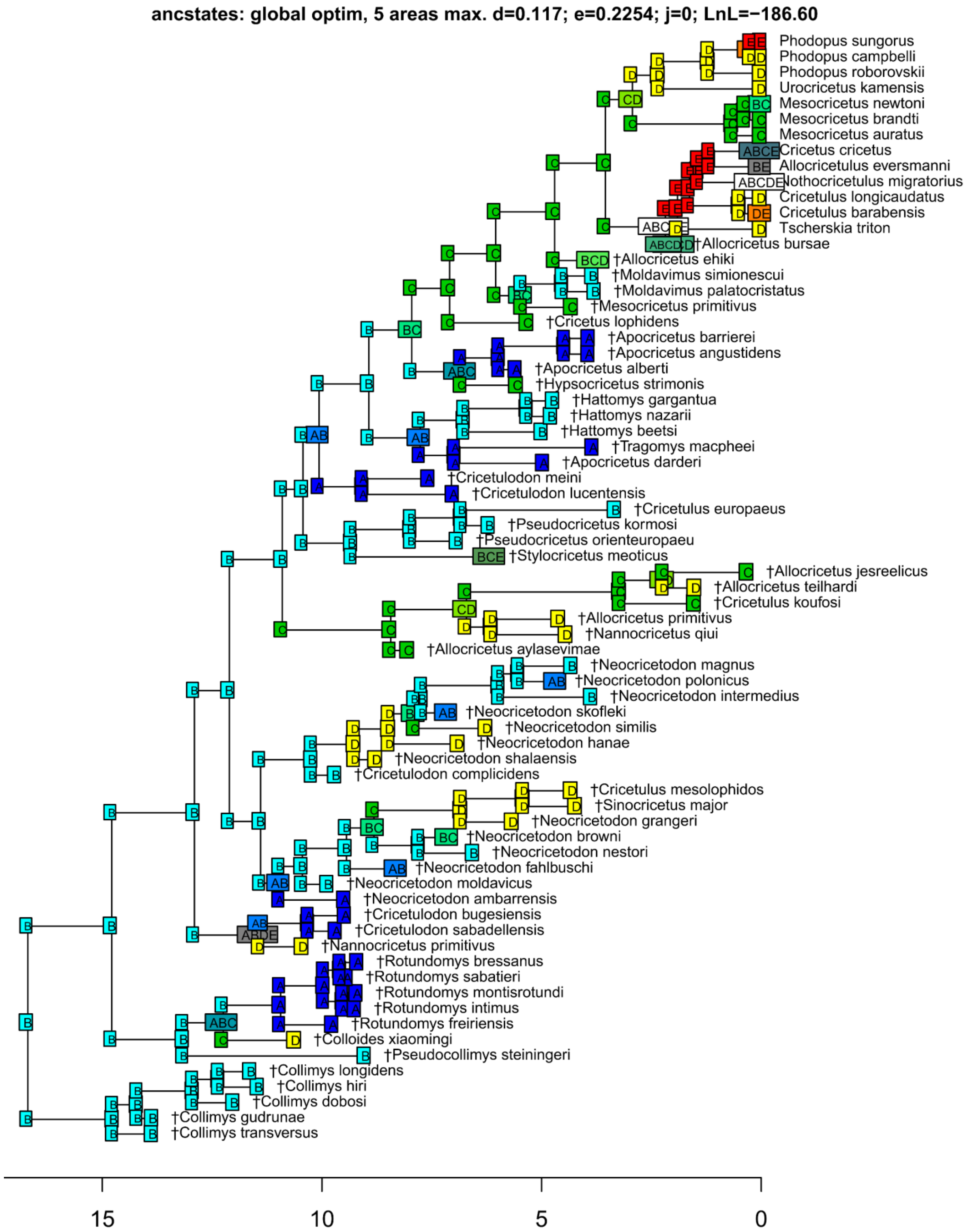

Figure S11. Reconstruction of the biogeographical history of Cricetinae based on the DIVALIKE model. Ancestral ranges are shown as most likely estimates. See Figure S3 for explanations of tip ranges and estimated ancestral ranges. Scale axis in Ma.

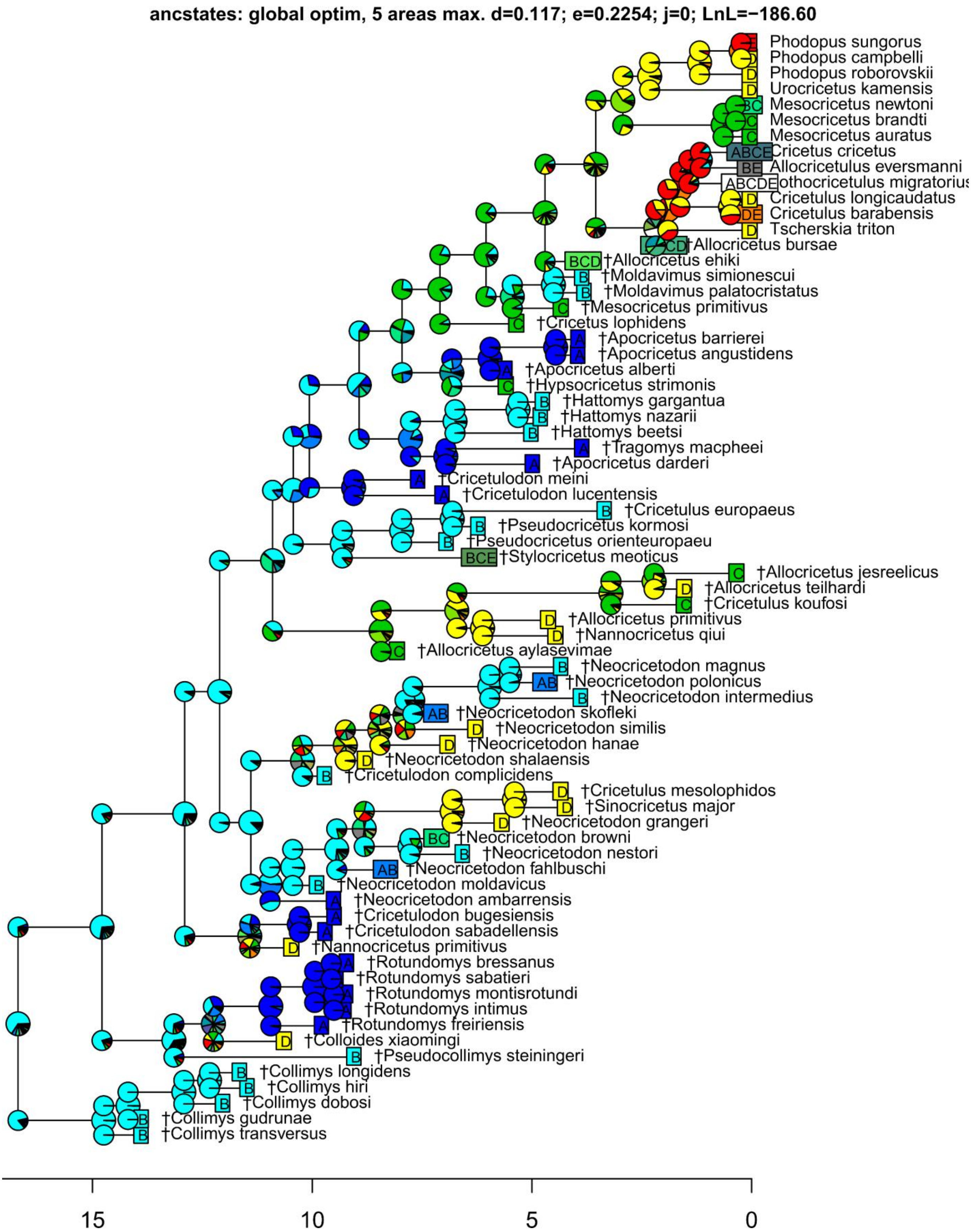

Figure S12. Reconstruction of the biogeographical history of Cricetinae based on the DIVALIKE model. Ancestral ranges are shown as pie-charts. See Figure S3 for explanations of tip ranges and estimated ancestral ranges. Scale axis in Ma.

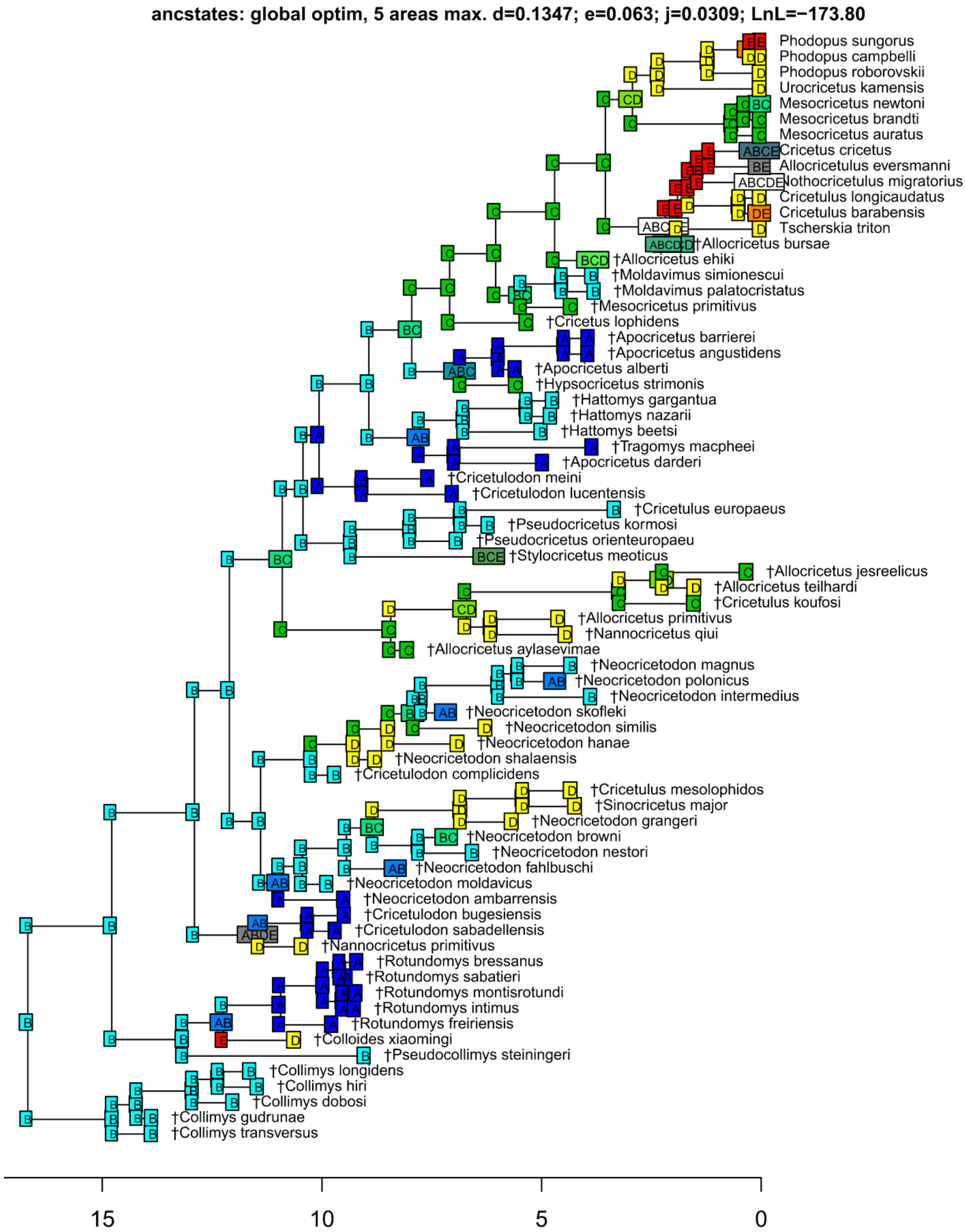

Figure S13. Reconstruction of the biogeographical history of Cricetinae based on the DIVALIKE+J model. Ancestral ranges are shown as most likely estimates. See Figure S3 for explanations of tip ranges and estimated ancestral ranges. Scale axis in Ma.

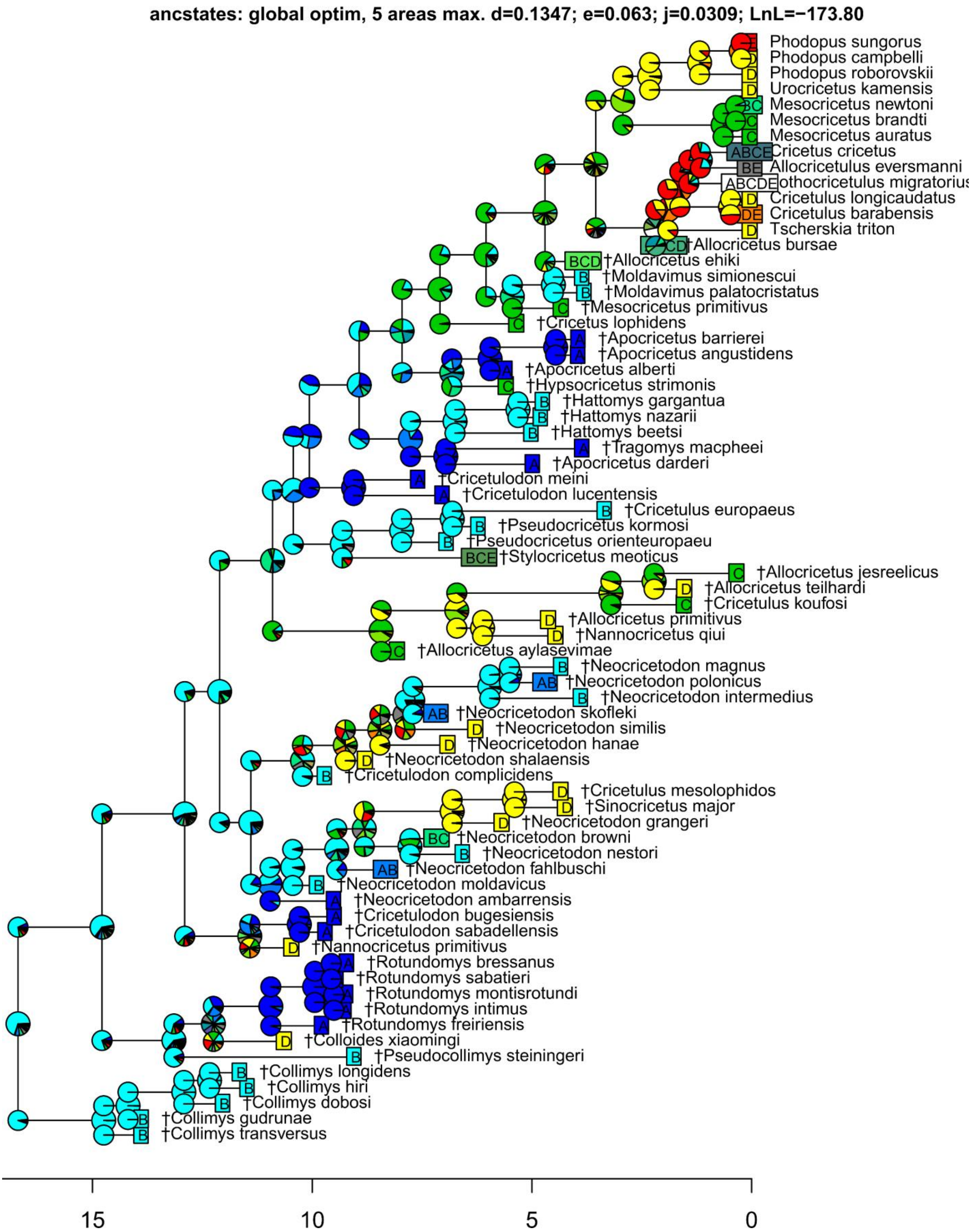

Figure S14. Reconstruction of the biogeographical history of Cricetinae based on the DIVALIKE+J model. Ancestral ranges are shown as pie-charts. See Figure S3 for explanations of tip ranges and estimated ancestral ranges. Scale axis in Ma.

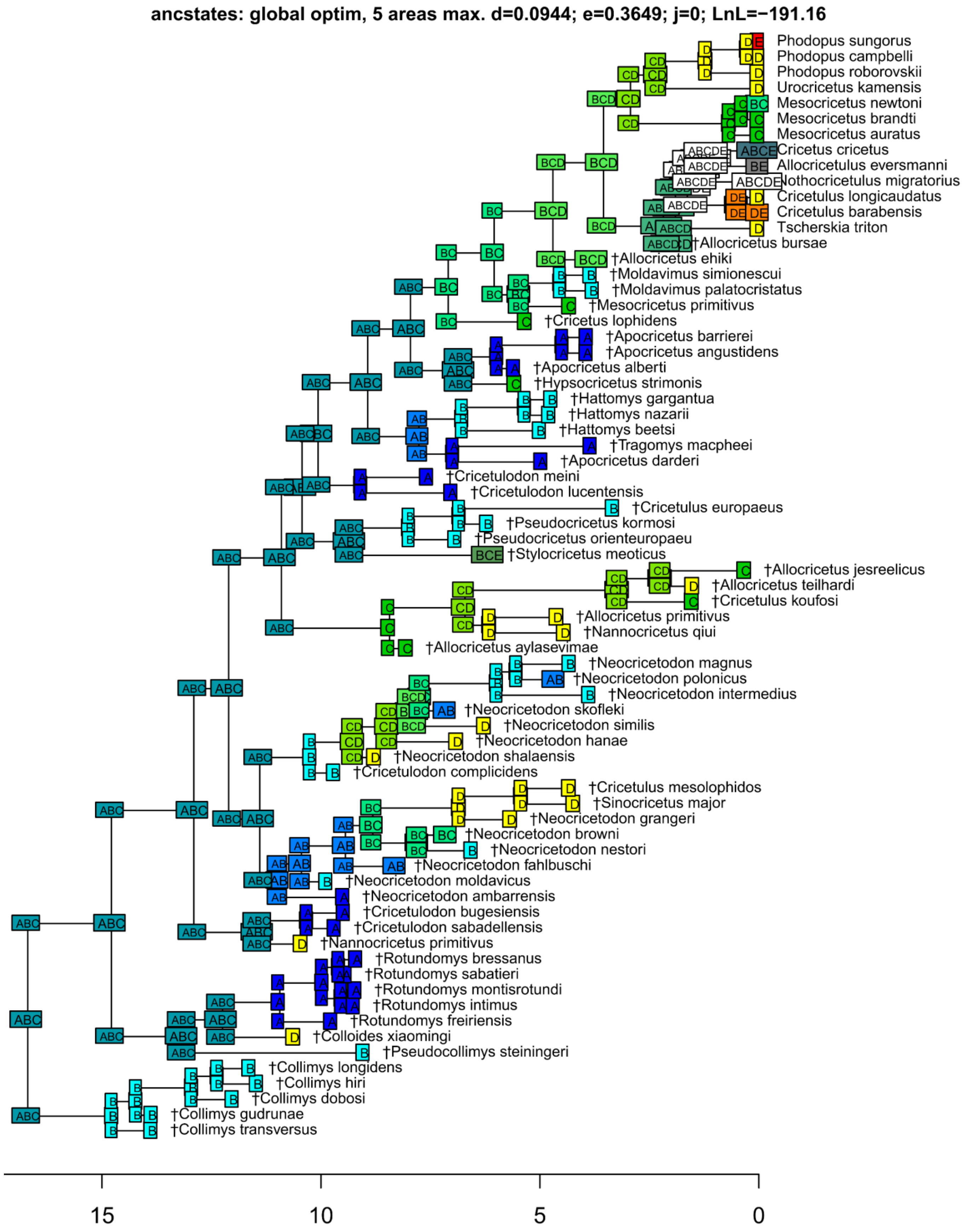

Figure S15. Reconstruction of the biogeographical history of Cricetinae based on the BAYAREALIKE model. Ancestral ranges are shown as most likely estimates. See Figure S3 for explanations of tip ranges and estimated ancestral ranges. Scale axis in Ma.

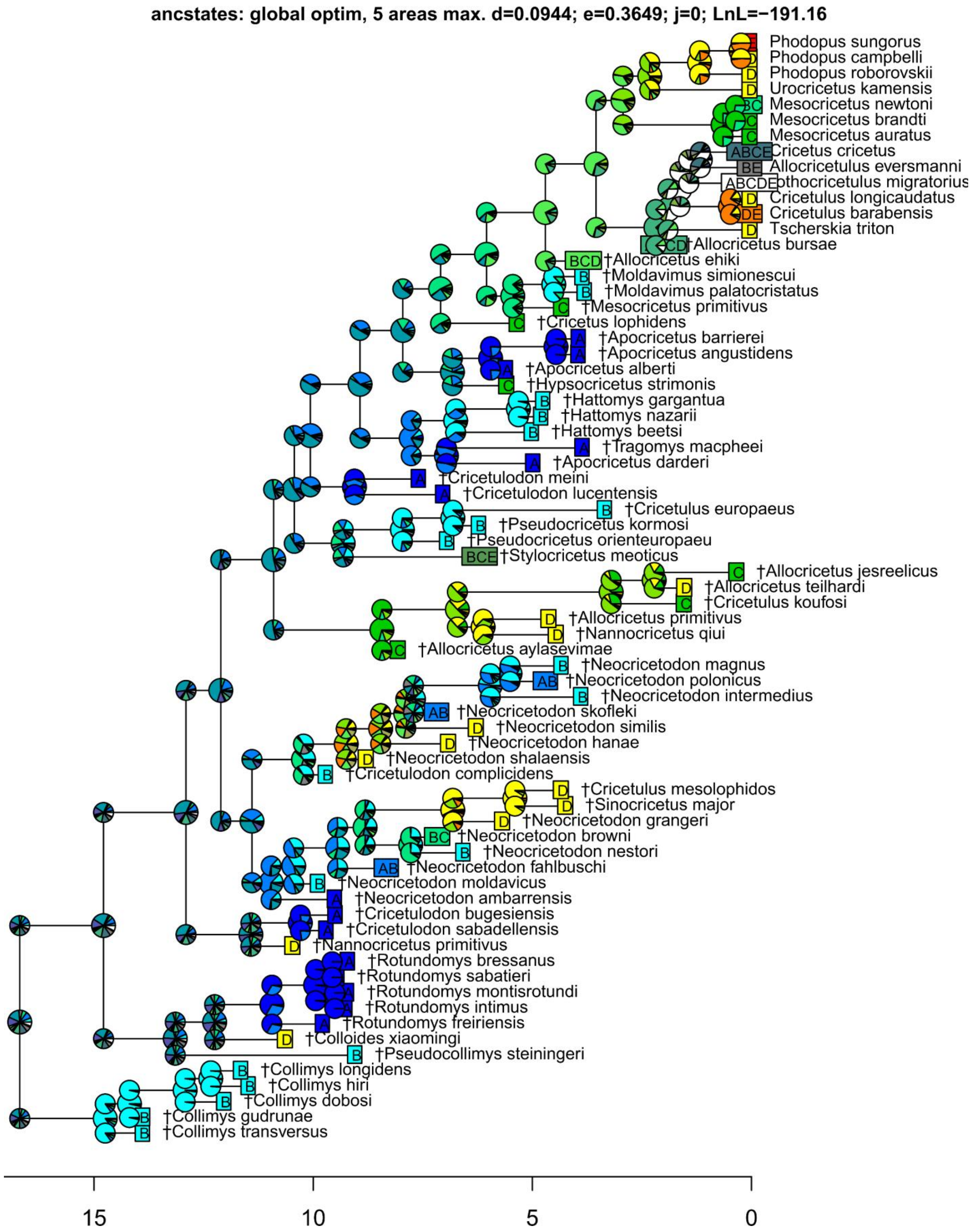

Figure S16. Reconstruction of the biogeographical history of Cricetinae based on the BAYAREALIKE model. Ancestral ranges are shown as pie-charts. See Figure S3 for explanations of tip ranges and estimated ancestral ranges. Scale axis in Ma.

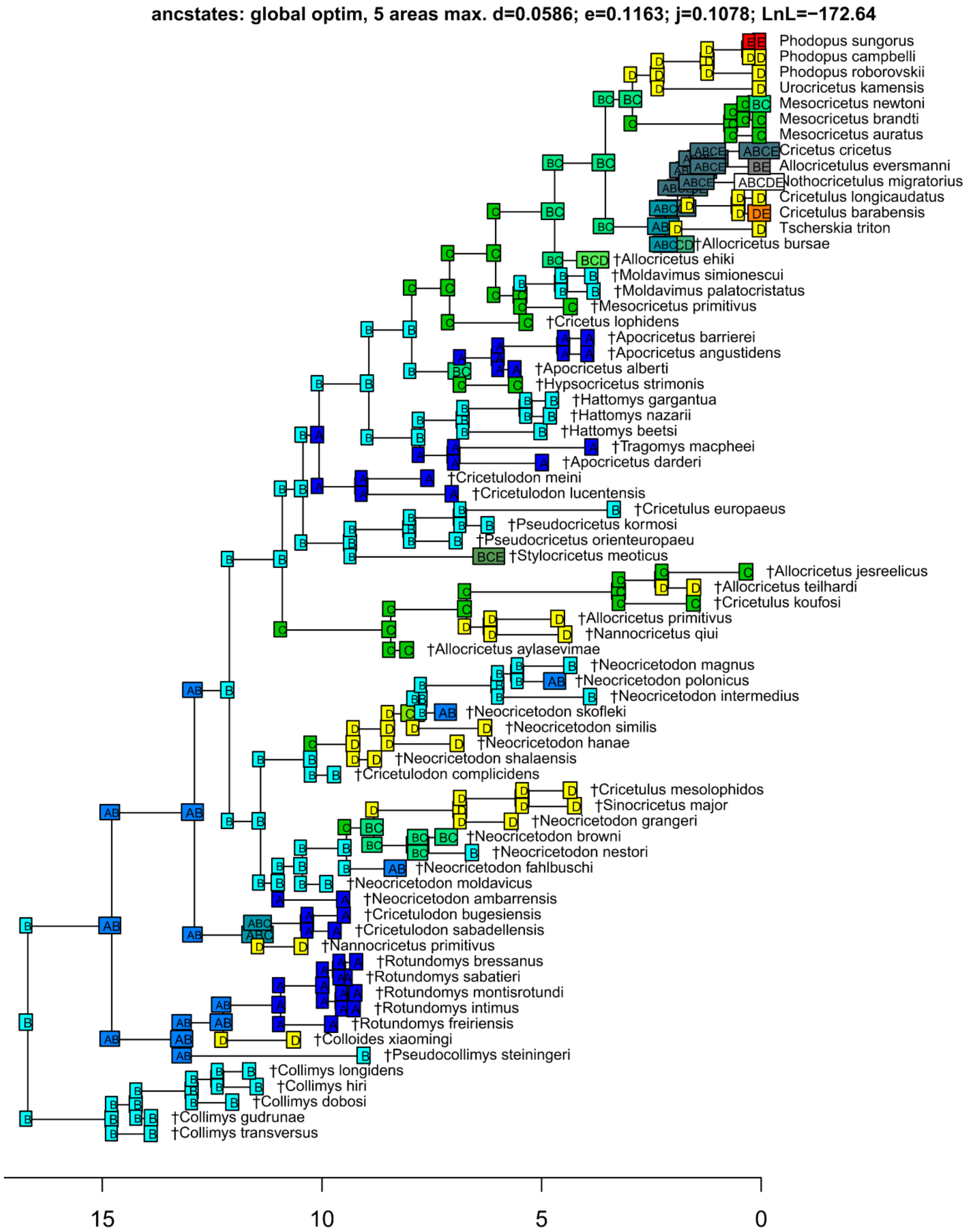

Figure S17. Reconstruction of the biogeographical history of Cricetinae based on the BAY-AREALIKE+J model. Ancestral ranges are shown as most likely estimates. See Figure S3 for explanations of tip ranges and estimated ancestral ranges. Scale axis in Ma.

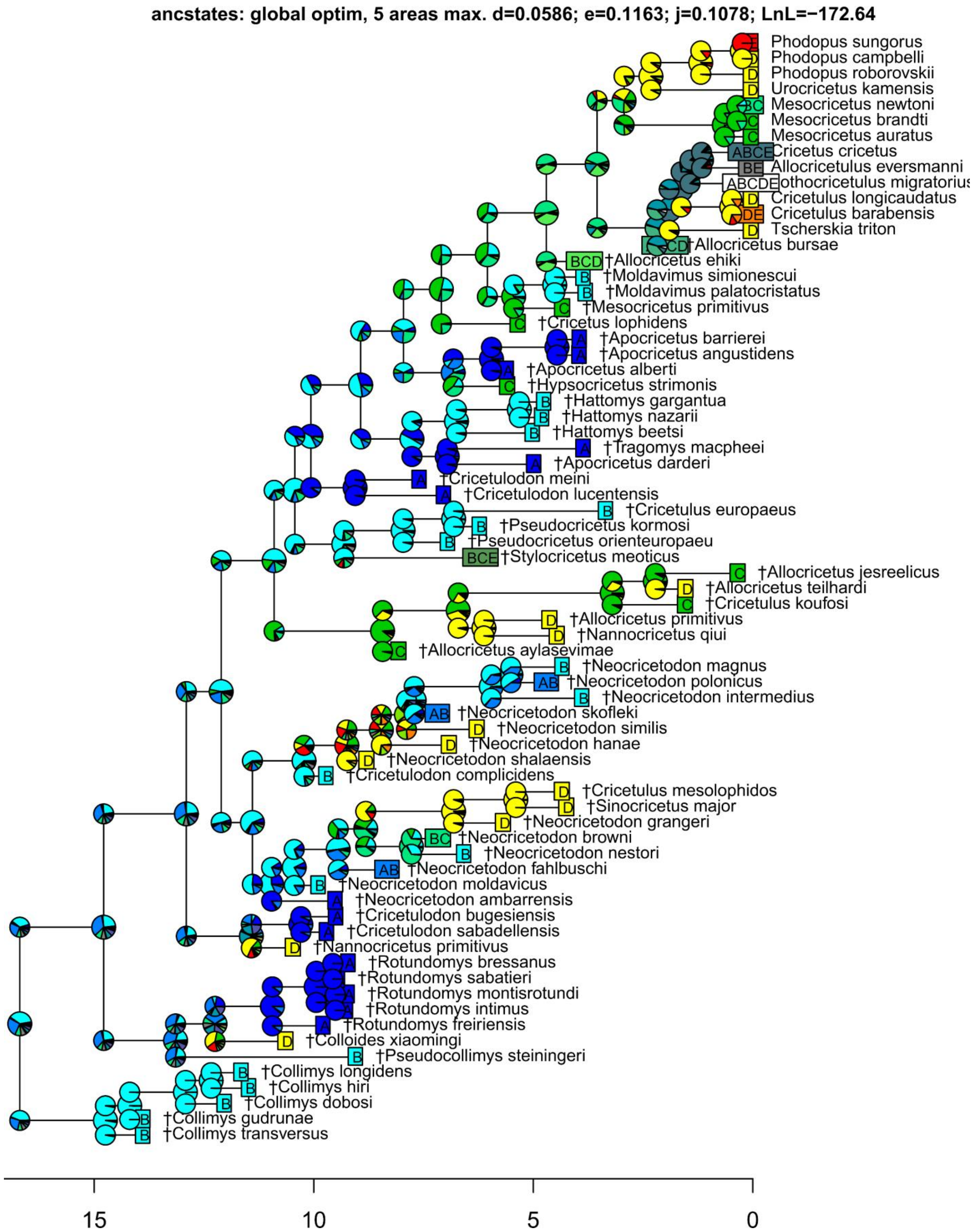

Figure S18. Reconstruction of the biogeographical history of Cricetinae based on the BAY-AREALIKE+J model. Ancestral ranges are shown as pie-charts. See Figure S3 for explanations of tip ranges and estimated ancestral ranges. Scale axis in Ma.
