# Supplementary material for: Total-evidence phylogeny reveals recent crown group radiation and biogeographical history of hamsters
Source: BMC Biol. 2026 Apr 9;24:117. doi: 10.1186/s12915-026-02581-z (PMC13173810; doi:10.1186/s12915-026-02581-z)
Supplement: Supplementary file 2 — Additional file 2: Text document (.pdf) with additional information about the studied taxa, including geographical distrubution, systematic information, stratigraphic range, oberserved material, and anatomical references. [file 12915_2026_2581_MOESM2_ESM.pdf]

Additional file 2 for

Total-evidence phylogeny reveals recent crown group radiation and biogeographical history of hamsters

Moritz Dirnberger[1], Pablo Peláez-Campomanes[2], Tiago R. Simões[3], Raquel López-Antoñanzas[1, 2]

[1]Institut des Sciences de l'Évolution de Montpellier, Université de Montpellier, CNRS, IRD, 34095 Montpellier, France

[2]Departamento de Paleobiología, Museo Nacional de Ciencias Naturales-CSIC, Madrid, Spain

[3]Department of Ecology and Evolutionary Biology, Princeton University, Briger Hall, Princeton-NJ, 08544, USA.

Additional information about the studied taxa and material

Overview

In total, Neogene and Quaternary combined, including all true hamsters, there are 28 genera including around 109 species, of which 82 (75 %) were included in the phylogenetic reconstruction of this study. All genera are represented by at least one species in this analysis, except the monospecific genera, †*Amblycricetus* Zheng, 1993, †*Bahomys* Chow & Li, 1965, *Cansumys* Allen, 1928, and †*Gromovia* Erbajeva, Alexeeva & Khenzykhenova, 2003, due to limited available material or descriptions [121–123].

Neogene taxa. There are 15 genera from the Neogene that are clearly associated with the Cricetinae. There is one additional genus, *Aepyocricetus* Li, Stidham, Ni & Li, 2017 that is possibly a member of the Cricetinae but the unique morphology of the genus impedes a confident association to the hamsters [72]. Out of the 65 species identified within these 15 genera, 55 (85%) could be added to the matrix, including at least one member of each genus (Tab. S1.1). The remaining species were either too limited in their material, with several molars not known, or no material or sufficient description was available to the authors and

therefore no meaningful number of characters could be coded. The information given here for *Apocricetus*, *Collimys*, *Cricetulodon*, *Hattomys*, *Neocricetodon*, *Pseudocricetus*, and *Rotundomys* follows in most parts Dirnberger et al. [11].

Quaternary taxa. There are 13 genera from the Quaternary that are clearly associated with the Cricetinae, including 25 extinct and 19 extant species (following [3]), although there are ongoing discussions, e.g., the status of '*Cricetulus griseus*' Milne-Edwards, 1867 [7, 10], which is here treated as a subspecies of *Cricetulus barabensis*. Out of the total 44 species, 27 (61 %) could be added to the matrix (Tab. S2.2), including, 13 (68 %) extant species, whose given type localities and distributions follow Musser and Carleton [77] and Kryštufek and Shenbrot [3], as well as references therein. The information given here for *Cricetus cricetus* and *Nothocricetulus migratorius* is compiled in most parts in Dirnberger et al. [11].

Table S2.1. Total number of accepted species and number of species included in the phylogenetic analysis of this study of all 15 extinct cricetine genera from the Neogene.

Genus	Accepted species	Species included here
Apocricetus	5	5 (100 %)
Collimys	7	5 (71 %)
Colloides	1	1 (100 %)
Cricetulodon	7	6 (86 %)
Hattomys	3	3 (100 %)
Hypsocricetus	1	1 (100 %)
Nannocricetus	3	3 (100 %)
Neocricetodon	20	15 (75 %)
Moldavimus	2	2 (100 %)
Pseudocollimys	1	1 (100 %)
Pseudocricetus	4	3 (75 %)
Rotundomys	6	5 (83 %)
Sinocricetus	3	3 (100 %)
Stylocricetus	1	1 (100 %)
Tragomys	1	1 (100 %)
**Total**	65	55 (85 %)

Table S2.2. Total number of accepted species and number of species included in the phylogenetic analysis of this study of all 13 cricetine genera from the Quaternary.

**Genus**	**Accepted species**	**Species included here**
†*Allocricetus*	9	7 (78 %)
Allocricetulus	2	1 (50 %)
†*Amblycricetus*	1	0
†*Bahomys*	1	0
Cansumys	1	0
Cricetulus	9	7 (78 %)
Cricetus	6	2 (33 %)
†*Gromovia*	1	0
Mesocricetus	6	4 (67 %)
Nothocricetulus	1	1 (100 %)
Phodopus	3	3 (100 %)
Tscherskia	2	1 (50 %)
Urocricetus	2	1 (50 %)
**Total**	44	27 (61 %)

Sampled taxa, including material and references studied for coding

Neogene taxa

Apocricetus Freudenthal, Mein & Martín Suárez, 1998

All 5 accepted species are included in the analysis:

Apocricetus alberti Freudenthal, Mein & Martín Suárez, 1998

Geographical distribution of occurrences: Spain

Type locality: Crevillente 6, Spain [61]

Holotype: Department Aardwetenschappen, University Utrecht, CR6 111, m1 (figured in [124], pl. 3, fig. 4, as *'Cricetus* cf. *kormosi'*)

Age range [Max–Min (Mean)]: 6.56–4.2 Ma (5.38 Ma)

Oldest occurrences: Arquillo 1, La Gloria 5, Las Casiones, Las Casiones superior, Masada del Valle 7, Purcal 23, Purcal 24A, Purcal 25, Purcal 25A, Valdecebro 3, Valdecebro 6, Villastar [125]

Youngest occurrence: Alcoy-N [125]

Anatomical references: [61, 124, 126–130]

Apocricetus angustidens (Depéret, 1890)

Geographical distribution of occurrences: France and Spain

Type locality: Serrat d'en Vacquer, France [131]

Holotype: Musée Guimet d'Histoire Naturelle, Lyon (Donnezan coll.), Pp 75, mandible (figured in [131], pl. 4, figs. 26, 26a, as *'Cricetus angustidens'*)

Age range: 4.27–3.03 Ma (3.65 Ma)

Oldest occurrences: Le Soler, Mont-Hélène, Nîmes, Sète, Serrat d'en Vacquer, Villeneuve de la Raho: MN 15 [61, 100]

Youngest occurrences: Puimoisson II, Lo Fournas 13: MN 16a [132, 133]

Problematic localities: Port-la-Nouvelle, Castelnou 1: mix of different ages [61]

Observed material: Faculty of Sciences, University Claude Bernard, Lyon (FSL): casts of type material. Bavarian State Collection for Palaeontology and Geology, Munich (SNSB-BSPG) 1967 XIV 19–24: m1, m2, m3, M1, M2, M3 from Nîmes

Anatomical references: [61, 134–136]

Remark: According to Freudenthal et al. [61], there is *A. angustidens* in the Spanish locality Vilafant. This locality was related to MN 14 [137], however on the basis that the species is *A.*

barrierei (see also [138]). As the species identification is not clear, the locality is not taken into account here.

Apocricetus barrierei (Mein & Michaux, 1970)

Geographical distribution of occurrences: France and Spain

Type locality: Chabrier, France [139]

Holotype: FSL 65356, M1 (figured in [139], pl. 1, fig. 1, as *'Cricetus barrierei'*)

Age range: 5.47–3.04 Ma (4.255 Ma)

Oldest occurrences: Celleneuve, Chabrier, Hautimagne, Vendargues: MN 14 [88, 140]

Youngest occurrence: Huéscar 3 [125]

Problematic localities: Castelnou 1, 3: mix of different ages [61]; Almudena 1D: age unknown [61, 141]

Observed material: FSL 65357, 65358, 65359: casts of type material: m1, m3, M3; cast of M3 and m2 from Hautimagne

Anatomical references: [61, 127, 136, 139, 141–147]

Remark: FSL 65359 is from Hautimagne according to Mein and Michaux [139] but from Chabrier (Type locality) according to the specimen label.

Apocricetus darderi Torres-Roig, Agustí, Bover & Alcover 2019

Single occurrence/type locality: Na Burgesa-1, Spain [81]

Holotype: Institut Mediterrani d'Estudis Avançats, Esporles, IMEDEA 94726, mandible (figured in [81], figs. 5(A), 7(A), 7(B))

Age: 5.333–3.6 Ma (4.467 Ma): Zanclean [81]

Anatomical reference: [81]

Apocricetus plinii (Freudenthal, Lacomba & Martin Suárez, 1991)

Geographical distribution of occurrences: France and Spain

Type locality: Crevillente 15, Spain [126]

Holotype: Naturalis Biodiversity Center, Leiden, RGM 402 10, M1 (figured in [126], pl. 4, fig. 1, as *Neocricetodon plinii*)

Age range: 7.67–6.27 Ma (6.97 Ma)

Oldest occurrence: Crevillente 5A [125]

Youngest occurrence: Otura 3 [125]

Problematic locality: Castelnou 1: mix of different ages [61]

Anatomical references: [61, 126]

Collimys Daxner-Höck, 1972

Of the 7 total species, 2 were not included in the analysis, namely *Collimys caucasicus* Tesakov, 2017 (in [44]) and *C. primus* Daxner-Höck, 1972, due to a limited amount of found specimens [44, 148]. The 5 species that are included are the following:

Collimys dobosi Hír, 2005

Geographical distribution of occurrences: Germany and Hungary

Type locality: Felsőtárkány 3/2, Hungary [49]

Holotype: Municipal Museum of Pásztó, Nógrád County Museums, No. 2003.79, M1 (figured in [49], fig. 1)

Age range: 12.2–11.1 Ma (11.6 Ma)

Oldest occurrence: Felsőtárkány 2/3 [149–151]

Youngest occurrence: Felsőtárkány 3/10 [151, 152]

Observed material: SNSB-BSPG 1979 XVIII 1–81: 81 isolated molars from Hillenlohe

Anatomical references: [47, 49, 151, 153, 154]

Collimys gudrunae Prieto & Rummel, 2009 [48]

Single occurrence/type locality: Petersbuch 31, Germany [48]

Holotype: Naturmuseum Augsburg, NMA 2007/1/2017, M1 (figured in [48], figs. 4 b1, b2)

Age: 13.9–13.8 Ma (13.85 Ma) [36, 48]

Anatomical reference: [48]

Collimys hiri Prieto & Rummel, 2009 [47]

Geographical distribution of occurrences: Germany

Type locality: Hammerschmiede 1, Germany [47]

Holotype: SNSB- BSPG 1973-XIX-182, m1 (figured in [47], fig. 2a)

Age range: 11.6–11.3 Ma (11.45 Ma)

Oldest occurrence: Hammerschmiede 1 [36, 47]

Youngest occurrence: Hammerschmiede 3 [36, 47]

Observed material: SNSB-BSPG 1973 XIX 182–185, 265, 266: type material: m1, m2, m3, M1, M2, M3; SNSB-BSPG 1980 XXVIII 1–58: 58 isolated molars from Hammerschmiede 3

Anatomical reference: [47]

Remark: The dating of the Hammerschmiede locality is based on the levels 4 and 5. Hammerschmiede 1 can be correlated to Hammerschmiede 5 [155]. As a more precise dating of Hammerschmiede 1 and 3 is not available, the range for the locality given in Hír et al. [149] and Prieto and Rummel [36] is taken.

Collimys longidens Kälin & Engesser, 2001

Single occurrence/type locality: Nebelbergweg, Switzerland [156]

Holotype: Natural History Museum Basel (NMB), Nbw 92, m1 (figured in [156], fig. 37b)

Age: 11.9–11.3 Ma (11.6 Ma)

Anatomical reference: [156]

Remark: According to Kälin and Engesser [156], the locality Nebelbergweg is related to early MN 9 but according to Hír and Kókay [154], it can also be late MN 7/8. The relative age of Nebelbergweg compared to Hammerschmiede is uncertain [47, 157]. Because of similarities of both faunas a similar or, following Prieto and Rummel [36], a slightly older age of Nebelbergweg is assumed.

Collimys transversus Heissig, 1995

Single occurrence/type locality: Steinheim am Albuch, Germany [158]

Holotype: NMB, Sth. 106 H1, M1 (figured in [158], pl. 1, fig. 7 and in [156], fig. 36e)

Age: 13.9–13.8 (13.85 Ma)

Anatomical references: [156, 158]

Remark: *Collimys transversus* is also reported from Bełchatów A, Poland (MN 9) [159]. The material is however low in number and is referred to as *Collimys* sp. by other authors [157].

Colloides Qiu & Li, 2016

The only species of the genus is included in the analysis. There is a second species found at Halajin Hushu, China, delimitated by its smaller size, but it is not named nor formally described [160]. The status as a genus is doubted by Tesakov et al. [44], who suggest a possible attribution to *Collimys*.

Colloides xiaomingi Qiu & Li, 2016

Geographical distribution of occurrences: China

Type locality: Balunhalagen, China [42]

Holotype: IVPP V19840, m1 (figured in [42], fig. 174G)

Age range: 11.2–7.65 Ma (9.425 Ma)

Oldest occurrence: Amuwusu: MN 9 [42, 160]

Youngest occurrence: Shala: MN 11 [42, 160]

Problematic locality: Balunhalagen: mix of different ages [42]

Anatomical references: [42, 160]

Cricetulodon Hartenberger, 1965

Of the 7 total species, 1 was not included in the analysis: *Cricetulodon cretensis* (de Bruijn & Meulenkamp, 1972), due to a limited number of specimens found [65, 161]. The 6 species that are included are the following:

Cricetulodon bugesiensis Freudenthal, Mein & Martín Suárez, 1998

Geographical distribution of occurrences: France

Type locality: Soblay, France [61]

Holotype: FSL 65897, m1 (figured in [61], pl. 1, fig. 1)

Age range: 9.9–8.75 Ma (9.325 Ma)

Oldest occurrence: Douvre: MN 10 [61, 162]

Youngest occurrences: Dionay, Lo Fournas 16-M: MN 10 [61, 162, 163]

Observed Material: FSL 65897: holotype m1; isolated molars from Soblay

Anatomical reference: [61]

Remark: Aguilar et al. [163] assumed MN 11 and an age of ca. 9.5 Ma for the locality Lo Fournas 16-M, which is considerably older than usual assumptions for the range of this zone, with a maximum of 8.9 or 8.75 Ma [125, 164]. We assume an association to MN 10, equal to the other French localities containing *Cricetulodon bugesiensis* [162].

Cricetulodon complicidens Topachevsky & Skorik, 1992

Single occurrence/type locality: Gritsev, Ukraine [32]

Holotype: Schmalhausen Institute of Zoology of National Academy of Sciences of Ukraine, (NASU-IZAN) No. 46, maxilla (figured in [32], fig. 4a)

Age: 9.78–9.6 Ma (9.69 Ma) [32]

Anatomical reference: [32]

Remarks: The species shows associations to *Neocricetodon* and should possibly be transferred to that genus [11].
Gritsev is Bessarabien in age, younger than Bujor (Buzhor) 1 and Calfa (Kalfa) and with reversed palaeomagnetism [165, 166]. Therefore it is probably correlated with C4Ar.2r or C4Ar.3r: lower border: 9.78 Ma [167, 168], the upper border follows the upper border of the Bessarabian: 9.6 Ma [169].

Cricetulodon hartenbergeri (Freudenthal, 1967)

Geographical distribution of occurrences: Spain and Turkey

Type locality: Pedregueras 2C, Spain [170]

Holotype: Institut de Paleontologia, Sabadell, PEC 585, m1 (figured in [170], pl. 1, fig. 14, as *’Rotundomys hartenbergeri’* and in [171], fig. 11, 4)

Age range: 10.6–9.49 Ma (10.045 Ma)

Oldest occurrence: Pedregueras 2A [172]

Youngest occurrence: Puente Minero 10 [173]

Observed Material: FSL: isolated molars from Pedregueras 2C. SNSB-BSPG1966 III 91–102: 2 m1, 2 m2, 2 m3, 2 M1, 2 M2 (broken), 2 M3 from Pedregueras 2C

Anatomical references: [61, 170, 171, 174–176]

Remark: *Cricetulodon hartenbergeri* was reported from Hostalets de Pierola Inferior (see [177]), which would be an even older location than Pedregueras 2A. The material was, however, recently assigned to another species, *Democricetodon nemoralis*, which is why this locality is not included here (see SOM S1 in [178]).

Cricetulodon lucentensis (Freudenthal, Lacomba & Martín Suárez, 1991)

Geographical distribution of occurrences: Spain

Type locality: Crevillente 17, Spain [126]

Holotype: Naturalis Biodiversity Center, Leiden, RGM 404 67, m1 (figured in [126], pl. 5, fig. 1, as *'Neocricetodon lucentensis'*)

Age range: 7.17–6.89 Ma (7.03 Ma)

Oldest occurrence: Crevillente 5 [125]

Youngest occurrence: Crevillente 17 [125]

Anatomical references: [61, 126]

Cricetulodon meini (Agustí, 1986)

Single occurrence/type locality: Casa del Acero, Spain [179]

Holotype: Institut de Paleontologia, Sabadell, FCA-237, M1 (figured in [179], pl. 2, fig. 8, as *'Kowalskia meini'*)

Age: 7.58–7.53 Ma (7.555 Ma) [125]

Anatomical references: [61, 179]

Remark: The material from Maramena, Greece described as *Allocricetus* cf. *ehiki* [83], shows similarities to *Cricetulodon meini* [13]. With an age of around 5.5 Ma [180] this would be a considerably younger occurrence but due to the uncertainties regarding the taxonomic identification, it is not considered here.

Cricetulodon sabadellensis Hartenberger, 1965

Geographical distribution of occurrences: Spain

Type locality: Can Llobateres, Spain [181]

Holotype: Institut de Paleontologia, Sabadell, CL 1392, maxilla (figured in [181], pl. 10, fig. c)

Age range: 9.98–9.12 Ma (9.55 Ma)

Oldest occurrences: Can Casablanques 1B, Can Coromines 2, Can Pallars de Llobateres [182–184]

Youngest occurrence: Torrent de Febulines M [61, 185]

Observed specimens: University Montpellier: type material

Anatomical references: [61, 181]

Remarks: Can Coromines 2 is associated to chron C5n.1r [186], Can Pallars de Llobateres belongs to the *Cricetulodon hartenbergeri – Progonomys hispanicus* interval subzone [182]. The lower boundaries are shared (9.98 Ma). Can Casablanques 1B is not precisely dated but can be assumed to fall in the same range.
According to Casanovas-Vilar et al. [182] the *Cricetulodon* species in Autopista de Rubí-Terrassa 8 and 3B, Spain are *C*. cf. *hartenbergeri* and *C. hartenbergeri*. But according to López-Antoñanzas et al. [38], it is *C. sabadellensis*, which would make Autopista de Rubí-Terrassa 8 the oldest occurrence but due to the uncertainties regarding the taxonomic identification, it is not considered here.

Hattomys Freudenthal, 1985

All 3 accepted species are included in the analysis:

Hattomys beetsi Freudenthal, 1985

Geographical distribution of occurrences: Italy

Type locality: Biancone 1, Italy [187]

Holotype: Naturalis Biodiversity Center, Leiden, RGM 263 775, mandible (figured in [187], pl. 1, fig. 1)

Age range: 7.02–4.19 Ma (5.605Ma)

Oldest occurrence: Biancone 1: MN 13 [88, 187, 188]

Youngest occurrence: Trefossi 1 or Chiro 19: MN 13/14 [187, 188]

Anatomical references: [187, 188]

Remark: For all localities of *Hattomys*, an age of MN 13/14 is used [88, 189, 190].

Hattomys gargantua Freudenthal, 1985

Geographical distribution of occurrences: Italy

Type locality: Chiro 2N, Italy [187]: MN 13/14? [88]

Holotype: Naturalis Biodiversity Center, Leiden, RGM 263 55, cranium with maxilla (figured in [187], pl. 3, fig. 3 and pl. 5, fig. 10–11)

Age range: 7.02–4.19 Ma (5.605Ma)

Observed material: SNSB-BSPG2004 IX 4: mandible with m1, m2, m3 from Monte Gargano, Fissure GP XI

Anatomical references: [187, 188]

Remark: For all localities of *Hattomys*, an age of MN 13/14 is used [88, 189, 190].

Hattomys nazarii Freudenthal, 1985

Geographical distribution of occurrences: Italy

Type locality: Nazario 2B, Italy [187]: MN 13/14? [88]

Holotype: Naturalis Biodiversity Center, Leiden, RGM 263 574, m1 (figured in [187], pl. 1, fig. 18)

Age range: 7.02–4.19 Ma (5.605 Ma)

Anatomical references: [187, 188]

Remark: For all localities of *Hattomys*, an age of MN 13/14 is used [88, 189, 190].

Hypsocricetus Daxner-Höck, 1992

The only species of the genus is included in the analysis:

Hypsocricetus strimonis Daxner-Höck, 1992

Single occurrence/type locality: Maramena, Greece [83]

Holotype: Department Aardwetenschappen, University Utrecht, MAA1 1812, maxilla (figured in [83], fig. 20, 1a, b)

Age: 5.8–5.3 Ma (5.55 Ma) [180]

Anatomical references: [83, 191]

Nannocricetus Schaub, 1934

There are 3 species described as *Nannocricetus*. A fourth one, ‘*Nannocricetus wuae*’ Zhang, Wang, Liu & Liu, 2011 is considered to belong to *Democricetodon*, due to several ‘primitive’ morphological traits [42, 72]. The material is relatively scarce, as there is e.g., no m3 and only a single partly broken M3 [192]. The remaining 3 species are included here:

Nannocricetus mongolicus Schaub, 1934

Geographical distribution of occurrences: China

Type locality: Ertemte 1, China (following [57])

Holotype: mandible (figured in [193], pl.-fig. 3, as cited in [57])

Age range: 7.14–2.58 Ma (4.86 Ma)

Oldest occurrence: Baode-JJG02 [194]

Youngest occurrences: several occurrences in the Nihewan basin ([195], see also [196])

Problematic localities: Bilutu and Balunhalagen: mix of different ages [42]

Anatomical references: [42, 56, 57, 60, 197]

Nannocricetus primitivus Zhang, Zheng & Liu, 2008

Geographical distribution of occurrences: China and Mongolia

Type locality: Lantian, Loc. 12, China [71]

Holotype: IVPP V15700, maxilla (figured in [71], fig. 1A)

Age range: 11.6–7.537 Ma (9.569 Ma)

Oldest occurrence: Yihachi [198, 199]

Youngest occurrence: Baogedawula [200]

Problematic localities: Bilutu and Balunhalagen: mix of different ages [42]

Anatomical references: [42, 56, 64, 71, 198, 201, 202]

Remark: The material of the single occurrence outside of China in Builstyn Khudag, Mongolia was first assigned to an affine form, *Nannocricetus* aff. *primitivus*, due to a ‘higher advanced’ morphology [64]. Recent studies assign the fossil however directly to *N. primitivus*, based on additional material of the species found in other Late Miocene localities [201].

Nannocricetus qiui Li, Stidham, Ni & Li, 2017

Single occurrence/type locality: Tibet Zanda ZD1001, China [72]

Holotype: IVPP V 23220, M1 (figured in [72], fig. 1A)

Age: 4.42 Ma [72, 203]

Anatomical reference: [72]

Neocricetodon Schaub, 1934

There are 31 species that have been associated with *Neocricetodon* at times [14, 42, 61, 63]. We follow Freudenthal et al. [61] in the question of synonymy of *Kowalskia* and *Neocricetodon* (see [11]) and for the European species but add *N. progressus* (see [204]). For the Chinese species, Qiu and Li [42] is followed (note that in the English translation only, *N. similis* is missing in their list ([42], pp. 633-634)). Differing from their list, we include *N. grangeri* but exclude *‘N. zhengi‘* (Qiu & Storch, 2000) and *‘N. neimengensis‘* (Wu, 1991) (see [14]). Therefore, there are 20 species included in the genus. Five of these species were not included in the analysis: *N. lavocati* (Hugueney & Mein, 1965), *N. lii* (Zheng, 1993), *N.*

schaubi Kretzoi, 1951, *N. seseae* Aguilar, Calvet & Michaux, 1995, *N. yinanensis* (Zheng, 1984), due to limited available material or descriptions [205–208]. Recently, one additional species of the genus was described as '*Kowalskia weni*' Feroz, Li, Fazal, Qiu & Ni, 2025 [62]. Due to the recency, the species could not be incorporated in this study. The 15 species that are included in the analysis are the following:

Neocricetodon ambarrensis Freudenthal, Mein & Martín Suárez, 1998

Geographical distribution of occurrences: France and Spain

Type locality: Ambérieu 2C, France [61]

Holotype: FSL 65907, m1 (figured in [61], pl. 3, fig. 1)

Age range: 9.9–8.75 Ma (9.325 Ma)

Oldest occurrence: Ambérieu 1: MN 10 [61, 162]

Youngest occurrence: Ambérieu 2A: MN 10 [61, 162]

Anatomical references: [14, 61]

Neocricetodon browni (Daxner-Höck, 1992)

Geographical distribution of occurrences: Greece and Moldova

Type locality: Maramena, Greece [83]

Holotype: Department Aardwetenschappen, University Utrecht, MAA1 1343, M1 (figured in [83], fig. 18/3, as *Kowalskia browni*)

Age range: 7.68–5.3 Ma (6.49Ma)

Oldest occurrence: Chimishlija (Cimișlia): lower border of MN 12 [209]

Youngest occurrence: Maramena [83, 180]

Anatomical references: [14, 83, 191]

Remark: The material from Maramena is mentioned as '*Kowalskia fahlbuschi*' in van der Meulen and van Kolfschoten [210] and in de Bruijn [211].

Neocricetodon fahlbuschi (Bachmayer & Wilson, 1970)

Geographical distribution of occurrences: Austria, Hungary, and Spain

Type locality: Kohfidisch, Austria [212]

Holotype: Museum of Natural History, Vienna (NHM), No. 1970/1393, maxilla (figured in [212], pl. 11, fig. 57 and in [213], pl. 2, fig. 9, as *Kowalskia fahlbuschi*)

Age range: 9.105–7.6 Ma (8.353 Ma)

Oldest occurrence: Sümeg [214]

Youngest occurrence: Ronda Oest de Sabadell A1 [182]

Observed material: SNSB-BSPG1972 XXII 1–4: 2 m2, m3, M2 from Kohfidisch

Anatomical references: [14, 45, 61, 83, 148, 212–215]

Remark: The type material is numbered KO 137 1-157 according to Freudenthal et al. [61]. Different ages of the locality Sümeg are given in several papers: MN 10 (e.g., [214]), MN 11 (e.g., [154]) or MN 12 (e.g., [216]). Bernor et al. [217] give an absolute age of 9.7–9.0 Ma. Here, Angelone and Čermák [218] are followed, who gave an overview of the discussions in the past and placed the locality between MN 10 and MN 11. Following their figure 1, the age is within chron C4An or C4r (9.105–8.125 Ma, following [167]).

Neocricetodon grangeri (Young, 1927)

Geographical distribution of occurrences: China

Type locality: Chia yu Tsun, Yushe-YS161, China (following [55])

Holotype: Uppsala University (Lagrelius coll.), Yu She 173, mandibles, cranium, postcranial fragments (figured in [63], fig. 16.1 and in [55], fig. 10.2)

Age range: 6.727–4.3 Ma (5.514 Ma) [55]

Oldest occurrences: Yushe-YS139, 142 [55, 219]

Youngest occurrences: Yushe-YS4, 97 [55, 120]

Anatomical references: [14, 55, 63]

Remark: According to Opdyke et al. [120], fig. 4.8, the locality Yushe-YS97 is in the Mazegou Formation correlated with C2An.3n: 3.596–3.33 Ma [167]. This age is considerably younger than the remaining localities of *N. grangeri*. Therefore, Tedford et al. [219], fig. 3.4 and Wu and Flynn [55], fig. 10.6 are followed, according to whom Yushe-YS97 is close in age to Yushe-YS4. That way, the age of Yushe-YS97, 4.3 Ma, as mentioned in Wu and Flynn [55], is congruent with the age of Yushe-YS4, as determined by Opdyke et al. [120], correlated with the base of C3n.1n: 4.3 Ma [167].

Neocricetodon hanae (Qiu, 1995)

Geographical distribution of occurrences: China

Type locality: Lufeng-shihuiba, China [220]

Holotype: Institute of Vertebrate Paleontology and Paleoanthropology of the Chinese Academy of Sciences, Beijing (IVPP), V10843, M1 (figured in [220], pl. 1, fig. 1, as *‘Kowalskia hanae‘*)

Age range: 7.15–6.2 Ma (6.675 Ma)

Occurrences: Leilao 9903–9906 [221, 222]: 7.15–7.10 Ma; Lufeng-shihuiba [220, 221]: ca. 6.9–6.2 Ma

Anatomical references: [14, 220]

Neocricetodon intermedius (Fejfar, 1970)

Single occurrence/type locality: Ivanovce, Slovakia [100]

Holotype: Súúg, OF 'Geologische Zentralanstalt', Prague, No. 652376, mandible (figured in [100], fig. 1a, as *'Kowalskia intermedia'*)

Age: 4.95–3.18 Ma (4.065 Ma) MN 15

Observed material: SNSB-BSPG 73272, 65125, 65128, 652382, -13, -92, -50, -88, -77, -93: casts of 9 isolated molars and a maxilla of 'collection Prague' from Ivanovce

Anatomical references: [14, 100]

Remarks: De Bruijn [211] and Koufos [223] mention '*Kowalskia intermedia*' in Lefkon, Greece. There is however no other reference mentioning this occurrence and between Lefkon (MN 10/11) and the type locality Ivanovce (MN 15) is such a large gap, that Lefkon is not included, here.
The locality Ivanovce is mentioned in several papers, a more precise age of the locality than MN 15 was however not found [100, 214, 224–227]. The boundaries of MN 15 follow Hordijk and de Bruijn [180] and van Dam et al. [125].

Neocricetodon magnus (Fahlbusch, 1969)

Geographical distribution of occurrences: Hungary, Italy and Poland

Type locality: Podlesice, Poland [98]

Holotype: Institute of Systematic and Experimental Zoology, Polish Academy of Sciences, Cracow, MF/823/1, m1 (figured in [98], pl. 17, fig. 1, as *‘Kowalskia magna‘*)

Age range: 5.47–3.18 Ma (4.325 Ma)

Oldest occurrences: Osztramos 1b, c, e: MN 14 [228]

Youngest occurrence: Osztramos 10: MN 15 [228]

Anatomical references: [98, 112, 214, 229, 230]

Remark: The occurrence of *N. magnus* in the latest Pliocene localities Zalesiaki 1B and Zamkowa Dolna Cave B is based on a single m1 and on a single m3, respectively [112]. Due to the limited material, these occurrences are not considered here.

Neocricetodon moldavicus (Lungu, 1981)

Geographical distribution of occurrences: Moldova

Type locality: Calfa (Kalfa), Moldova [231]

Holotype: Department of Geography, Tiraspol State University, Kishinev, TSU Caf-2425, mandible (figured in [231], pl. 15, figs. 1a, b, as *‘Kowalskia moldavica‘* and in [14], figs. 2.2, 4.2, 4.1)

Age range: 9.937–9.786 Ma (9.862 Ma)

Occurrences: Bujor (Buzhor) 1, Calfa [14, 231]

Observed material: FSL: TSU Caf-2425: casts of holotype: m1–3, M1–3

Anatomical references: [14, 231]

Remarks: Bujor 1 and Calfa are associated with the Bessarabian, probably correlated with C5n1n: 9.937–9.786 Ma ([167], see [168]).

Neocricetodon moldavicus is mentioned from two additional localities in Moldova, Hirova (Girovo) and Veveriţa 1 (Veveritsa 1) [232]. This material does, however, not resemble *N. moldavicus* or any other species of *Neocricetodon*, according to Sinitsa and Delinschi [14].

Neocricetodon nestori (Engesser, 1989)

Single occurrence/type locality: Podere Santa Croce 1, Baccinello V3, Italy [233]

Holotype: NMB, Bac. 242, M2 (figured in [233], fig. 16A, as *'Kowalskia nestori'*)

Age: 6.7–6.4 Ma (6.55 Ma) [233, 234]

Anatomical references: [14, 233]

Remark: The type locality is given as 'Podere Santa Croce 1' [88, 233], whereas sometimes just the faunal horizon 'Baccinello V3' is mentioned [229, 235]. The age range follows Rook [234], where only the horizon is given.

Neocricetodon occidentalis (Aguilar, 1982)

Geographical distribution of occurrences: Spain

Type locality: Crevillente 2, Spain [236]

Holotype: Department Aardwetenschappen, University Utrecht, CR2 63, M1 (figured in de [124], pl. 4, fig. 10, as *'Kowalskia fahlbuschi'*)

Age range: 8.51–7.62 Ma (8.065 Ma)

Oldest occurrence: Masada Ruea 2 [125]

Youngest occurrence: Concud Barranco de las Calaveras [125]

Anatomical references: [14, 61, 124, 126, 237]

Remark: Freudenthal et al. [61], also mentioned *N. occidentalis* in the considerably older locality Masia del Barbo 2B but expressed reservations due to a limited number of specimens.

Neocricetodon polonicus (Fahlbusch, 1969)

Geographical distribution of occurrences: France, Hungary, Poland, and Ukraine

Type locality: Podlesice, Poland [98]

Holotype: Institute of Systematic and Experimental Zoology, Polish Academy of Sciences, Cracow, MF/822/1, mandible (figured in [98], pl. 16, fig. 1a–c, as *'Kowalskia polonica'*)

Age range: 5.47–3.18 Ma (4.325 Ma)

Oldest occurrences: Hauterives: MN 14 [61]; Osztramos 1b, c, e: MN 14 [214, 228, 230]; Podlesice: MN 14 [61, 98, 238, 239]

Youngest occurrence: Osztramos 10: MN 15 [228]

Observed material: SNSB-BSPG1976 I 2–7: m1–m3, M1–M3 from Podlesice

Anatomical references: [14, 61, 98, 112, 214, 230]

Remarks: The occurrence of *N. polonicus* in the latest Pliocene to Pleistocene localities Zamkowa Dolna Cave B and Rębielice Królewskie 1A is based on one m2 and m3 and on a single m2, respectively [112]. Due to the limited material, these occurrences are not considered here.

There are also 6 additional occurrences in China of *N. neimengensis* and *N. zhengi*, that are synonyms of *N. polonicus* according to Sinitsa and Delinschi [14]. They are not considered here. If they are included, Ertemte might be the oldest occurrence (MN 12) [57].

Neocricetodon progressus (Topachevsky & Skorik, 1992)

Geographical distribution of occurrences: Romania and Ukraine

Type locality: Novo-Elizavetovka 2, Ukraine (see [204])

Holotype: NASU-IZAN No. 47, maxilla (figured in [32], fig. 19, as *‘Kowalskia progressa‘*)

Age range: 8.5–7.65 Ma (8.075 Ma)

Occurrences: Dolhești-1: MN 11 [66]; Palievo [204, 240]; Maloye, Novo-Elizavetovka 2, Otradovo: upper MN 11 [204, 240, 241]

Anatomical references: [14, 32, 204]

Neocricetodon shalaensis (Qiu & Li, 2016)

Geographical distribution of occurrences: China

Type locality: Shala, China [42]

Holotype: IVPP V19859, M1 (figured in [42], fig. 182A, as *‘Kowalskia shalaensis‘*)

Age range: 11.2–7.537 Ma (9.368 Ma)

Oldest occurrence: Halajin Hushu: MN9-10 [160]

Youngest occurrence: Baogedawula [200]

Anatomical references: [42, 160]

Neocricetodon similis (Wu, 1991)

Geographical distribution of occurrences: China

Type locality: Ertemte 2, China [57]

Holotype: IVPP V8723.50, M1 (figured in [57], pl. 2, fig. 22, as *'Kowalskia similis'*)

Age range: 6.5–3.6 Ma (5.05 Ma)

Oldest occurrence: Ertemte 2 [42, 57, 242]

Youngest occurrence: Daodi, Laowogou-2 [195]

Anatomical references: [14, 57]

Remark: Qiu and Li [42] 'arbitrarily' assign five molars from Balunhalagen, China and four molars from Bilutu, China to *N. similis* ('*Kowalskia similis*'), due to a close geographic position to Ertemte and Harr Obo. Especially Balunhalagen (up to 11 Ma) is however considerably older than Ertemte and Harr Obo and due to poor material and an arbitrary assignment [42], these localities are not considered here.

Neocricetodon skofleki (Kordos, 1987)

Geographical distribution of occurrences: Austria, France, Hungary and Slovakia

Type locality: Tardosbánya, Hungary [214]

Holotype: Paleontological Department, Hungarian Natural History Museum, Budapest, V.86.2, mandible (figured in [214], pl. 1, fig. 1, as *'Karstocricetus skofleki'*)

Age range: 8.75–6.9 Ma (8.2 Ma)

Oldest occurrence: Ambérieu 3: MN 11 [61, 162]

Youngest occurrences: Tardosbánya, Šalgovce: MN 12 [214, 243]

Observed material: FSL: casts of M1–3, m1–3 from Tardosbánya

Anatomical references: [14, 45, 61, 148, 214]

Remark: The material of Dionay and Ambérieu 2C, is only associated with *N. skofleki* with doubt in Freudenthal et al. [61], while the material of Ambérieu 1 and 2A is not mentioned at all.

Moldavimus Samson & Radulescu, 1973

'*Cricetulus simionescui*' Schaub, 1931, described from Bereşti, Romania, was assigned to the newly erected subgenus *Moldavimus* Samson & Radulescu, 1973 and later to the new genus *'Odessamys'* Topachevsky & Skorik, 1992 [32, 84, 85]. Recently, Crespo et al. [86], re-examined the material and transferred it to *Allocricetus* indet. The species *M. palatocristatus* and M. *simionescui* are both included in this study as *Moldavimus,* due to the priority of this older name:

Moldavimus palatocristatus Topachevsky & Skorik, 1992

Geographical distribution of occurrences: Moldova and Ukraine

Type locality: Odessa Catacombs, Ukraine [32]

Holotype: NASU-IZAN No. 24, maxilla (figured in [32], fig. 53a, as *'Odessamys palatocristatus'*)

Age range: 4.27–3.18 Ma (3.725 Ma), Tătăreşti: MN 15 [244]

Anatomical reference: [32]

Moldavimus simionescui (Schaub, 1931)

Geographical distribution of occurrences: Moldova, Romania and Russia

Type locality: Bereşti, Romania [84]

Lectotype: mandible without collection number, stored either in the Laboratory of Palaeontology, Faculty of Geology and Geophysics, University of Bucharest (see [85]) or in the 'Emil Racoviță' Institute of Speleology, Romanian Academy, Bucharest (see [86]) (figured in [84], fig. 1, as *'Cricetulus simionescui'*)

Age range: 5.47–3.18 Ma (4.325Ma), Bereşti: MN 14–MN 15 [86]

Anatomical references: [32, 84–86]

Pseudocollimys Daxner-Höck, 2004

The status as a genus is doubted by Tesakov et al. [44].The only species of the genus is included in the analysis.

Pseudocollimys steiningeri Daxner-Höck, 2004

Single occurrence/type locality: Schernham bei Haag, Austria [43]

Holotype: NHM 2003z0043/0001, M1 (figured in [43], pl. 1, fig. 1a-c)

Age: 9.1–8.9 Ma (9 Ma) [245]

Anatomical references: [43, 45]

Pseudocricetus Topachevsky & Skorik, 1992

Of the 4 total species, one was not included in the analysis: *Pseudocricetus antiquus* Topachevsky & Skorik, 1992, due to a limited amount of found specimens. The 3 species that are included are the following:

Pseudocricetus kormosi (Schaub, 1930)

Geographical distribution of occurrences: Ukraine and Hungary

Type locality: Polgardi 2, Hungary [59, 73]

Lectotype: Palaeovertebrate Collection of the Hungarian Geological Institute, Budapest, Ob. 4185, mandible (figured in [73], fig. 8, 1, as *'Cricetus kormosi'*)

Age range: 7.02–5.6 Ma (6.31 Ma)

Occurrences: Polgardi 2 [73]; Andreevka [51, 241]; Novoukrainka 1, Orekhova, [32, 51, 241]; Odessa Pontian Lectostratotype [32, 241]; Nerubayskoe/NATI 2 [246]

Observed Material: FSL: casts of type material: Ob. 4185 M1–3 and M1–2, M1; originals from NMB & Paleontological Department, Hungarian Natural History Museum, Budapest. SNSB-BSPG 1979 I 154: casts of type material: 3 maxilla fragments with M1, M2 and 3 mandible fragments with m1, m2, m3 of the Hungarian Natural History Museum, Budapest

Anatomical references: [32, 51, 73, 124]

Remark: Following Nesin and Storch [241] all the Ukrainian localities belong to MN 13 and the upper Maeotian or Pontian. Therefore, the lower border of MN 13 of van Dam et al. [125], and the upper border of the Pontian is taken (following [168, 247]). The Hungarian locality Polgardi 2 (MN 13, Pontian) is assumed to fall in this range as well [73].

Pseudocricetus orienteuropaeus Topachevsky & Skorik, 1992

Geographical distribution of occurrences: Ukraine

Type locality: Protopovka 3, Ukraine (see [51])

Holotype: NASU-IZAN No. 50, mandible (possibly figured in [32], fig. 34a or 36, there is no collection number given for the figured mandibles)

Age range: 7.68–6.04 Ma (6.86 Ma)

Occurrences: Belka 2; Egorovka 1, 2; Kubanka 2; Novo-Elizavetovka 3; Protopopovka 3 [51, 240, 241, 248]

Anatomical references: [32, 51]

Remark: All the localities belong to MN 12 and the Maeotian. Therefore, the lower border of MN 12 of van Dam et al. [125], and the upper border of the Maeotian is taken (following [168, 247]). As there is disagreement about the border of MN 12 to MN 13 in the Maeotian, the here used absolute ages overlap.

Pseudocricetus polgardiensis (Freudenthal & Kordos, 1989)

Geographical distribution of occurrences: Hungary

Type locality: Polgardi 4, Hungary [73]

Holotype: Palaeovertebrate Collection of the Hungarian Geological Institute, Budapest, V. 14033 (Vt. 126), mandible (figured in [73], figs. 1, 1; 5, as *'Cricetus polgardiensis'*)

Age range: 7.02–5.6 Ma (6.31 Ma)

Occurrences: Polgardi 4 [73]; Polgardi 3 [73, 249, 250]

Anatomical reference: [73]

Remark: The age is assumed to be MN 13/Pontian [73]. The age of Polgardi 2 is followed (see remark of *P. orienteuropaeus*).

Rotundomys Mein, 1965

Of the 6 total species, 1 was not included in the analysis: *Rotundomys mundi* Calvo, Elizaga, López-Martínez, Robles & Usera, 1979, due to the limited available material (missing M1 [251]). The 5 species that are included are the following:

Rotundomys bressanus Mein, 1975

Geographical distribution of occurrences: France and Spain

Type locality: Soblay, France [50]

Holotype: FSL 65443, M1

Age range: 9.9–8 Ma (8.95 Ma)

Oldest occurrence: Soblay: MN 10 [162]

Youngest occurrence: Bernardière: Early MN 11 [162]

Observed material: FSL: casts of type material

Anatomical references: [32, 38, 50, 61, 238]

Remark: A locality ‘Santa Margarida’ is mentioned with an occurrence of *R. bressanus* with no further comment in Freudenthal et al. [61]. There is a locality with that name from Portugal mentioned in Antunes and Mein [252], with an age of ca. 6 Ma, which is younger than all other localities of *R. bressanus*. In NOW, there is a locality ‘Santa Margarida’ in Spain [78],

associated with MN 5, which is too old for *R. bressanus*. For neither of the two localities, *R. bressanus* is mentioned in the respective references.

Rotundomys freiriensis Antunes & Mein, 1979

Geographical distribution of occurrences: Portugal and Spain

Type locality: Freiria do Rio Manor, Portugal [253]

Holotype: Department of Earth Sciences, NOVA School of Science and Technology, NOVA University Lisbon, M1 (figured in [253], pl. 1, fig. a, i)

Age range: 9.87–9.62 Ma (9.745 Ma)

Occurrences: Asseiceira [254]; Freiria do Rio Manor [253]; Autopista de Rubí-Terrassa 7C, 11 [182]

Observed material: FSL: casts of type material

Anatomical references: [38, 61, 253, 254]

Remark: The localities Autopista de Rubí-Terrassa 7C and 11 are associated to the *Cricetulodon sabadellensis* + *Progonomys hispanicus* concurrent range subzone (= 9.73–9.65 Ma) of Casanovas-Vilar et al. [182], which falls within the range of the J1 subzone (= 9.87–9.62 Ma) of van Dam et al. [173], that Asseiceira and Freiria do Rio Manor are correlated to [255].

Rotundomys intimus López-Antoñanzas, Peláez-Campomanes & Álvarez-Sierra, 2014

Geographical distribution of occurrences: Spain

Type locality: Cerro de los Batallones 5, Spain [38]

Holotype: National Museum of Natural Sciences, Madrid, BAT5'10-07, mandible (figured in [38], fig. 3A)

Age range: 9.71–8.75 Ma (9.23 Ma)

Oldest occurrence: Cerro de los Batallones 10 [38, 256, 257]

Youngest occurrence: Cerro de los Batallones 3 [38, 256, 257]

Anatomical reference: [38]

Remark: The Cerro de los Batallones system is placed in the local subzones J2 or J3 [256], so this range is taken from van Dam et al. [173].

Rotundomys montisrotundi (Schaub, 1944)

Geographical distribution of occurrences: France and Spain

Type locality: Montredon, France [46, 258]

Holotype: NMB, A Mo 849, m1 (figured in [46], fig. 1a and in [238], fig. 2(19a))

Age range: 9.9–8.75 Ma (9.325 Ma)

Occurrences: Ampudia 3; Camí de Can Tarumbot 2, 3; Can Casablanques; Can Casablanques 2; Chabeuil - Les Bourbons; La Tarumba 1; Lo Fournas 6, 7, 1993; Montredon; Racor: MN 10 [162, 182, 259, 260]

Anatomical references: [38, 46, 61, 236, 238, 258, 259, 261]

Rotundomys sabatieri Aguilar, Michaux & Lazzari, 2007

Geographical distribution of occurrences: France

Type locality: Lo Fournas 16-M, France [261]

Holotype: Faculty of Science, University of Montpellier, Fou 16-M no 395, m1 (figured in [261], pl. 2, fig. 2)

Age range: 9.9–8.75 Ma (9.325 Ma)

Oldest occurrence: Lo Fournas 6 [163, 261]

Youngest occurrence: Lo Fournas 16-M [163, 261]

Anatomical references: [38, 261]

Remark: Both localities are considered MN 10, which fits with the age given for Lo Fournas 16-M (± 9.5 Ma) in [163].

Sinocricetus Schaub, 1930

There are 3 species, that are undoubtedly members of *Sinocricetus*. Additionally, '*Kowalskia complicidens*' Topachevsky & Skorik, 1992 was excluded from *Neocricetodon* (or '*Kowalskia*') by Daxner-Höck et al. [63] and tentatively transferred to *Sinocricetus* by Sinitsa and Delinschi [14], as was '*Kowalskia dalinica*' Wang, 1988 (*Sinocricetus dalinicus*) by Xie et al. [53], which is however only known from a single m1 [53, 262]. Recently, an additional species, *Sinocricetus primus* Qiu, Flynn, Wang & Li, 2026, was described [263]. Due to the recency, the species could not be incorporated in this study. The remaining 3 species of *Sinocricetus*, are included here:

Sinocricetus major Li, 2010

Geographical distribution of occurrences: China

Type locality: Gaotege DB02, China [56]

Holotype: IVPP V17022, m2 (figured in [56], fig. 1G)

Age range: 4.3–4.1 Ma (4.2 Ma)

Oldest occurrence: Gaotege DB02 [56, 264]

Youngest occurrence: Gaotege DB03-1 [56, 264]

Anatomical reference: [56]

Sinocricetus progressus Qiu & Storch, 2000

Geographical distribution of occurrences: China

Type locality: Bilike, China [60]

Holotype: IVPP V11914, maxilla (figured in [60], pl. 8, fig. 1)

Age range: 5.333–2.58 Ma (3.957 Ma)

Oldest occurrence: Daodi, Laowogou-2 [195]

Youngest occurrences: several occurrences in the Nihewan basin ([195]; see also [196])

Problematic locality: Bilutu: mix of different ages [42]

Anatomical references: [42, 56, 60]

Remark: *Sinocricetus zdanskyi* from Daodi, Laowogou (see [265]) is considered *S. progressus* [195, 266].

Sinocricetus zdanskyi Schaub, 1930

Geographical distribution of occurrences: China

Type locality: Ertemte 1, China [57, 59]

Lectotype: mandible (figured in [193], fig. 30, as cited in [57])

Age range: 8.75–4.63 Ma (6.69 Ma)

Oldest occurrence: Shala: MN 11

Youngest occurrences: Lingtai Wenwanggou(93002)-3, Xiaoshigou-2 [267]

Problematic localities: Bilutu and Balunhalagen: mix of different ages [42]

Anatomical references: [42, 56–59, 268]

Remark: According to Li [56], *S. zdanskyi*, that has been mentioned in the Zones IV and V of Lingtai, Gansu Province, is actually *S. progressus*. In the descriptions of these localities, however, *S. zdanskyi* is not mentioned in Zone IV or V at all [267, 269–271].

Stylocricetus Topachevsky & Skorik, 1992

The only species of the genus is included in the analysis:

Stylocricetus meoticus Topachevsky & Skorik, 1992

Geographical distribution of occurrences: Kazakhstan, Ukraine and Russia

Type locality: Cherevichnoe 3, Ukraine [32]

Holotype: NASU-IZAN No. 51, maxilla (possibly figured in [32], fig. 51, there is no collection number given for the figured maxilla)

Age range: 7.68–5.33 Ma (4.55 Ma)

Oldest occurrences: Cherevichnoe 3, Novo-Elizavetovka 3, Protopopovka 3 [32]

Youngest occurrence: Seletey 1A: MN 13 [272]

Anatomical reference: [32]

Remark: For the age of Cherevichnoe 3, Novo-Elizavetovka 3, Protopopovka 3, see the remark of *Pseudocricetus orienteuropaeus*.

Tragomys Agustí, Bover & Alcover, 2012

The only species of the genus is included in the analysis:

Tragomys macpheei Agustí, Bover & Alcover, 2012

Single occurrence/type locality: Caló den Rafelino, Spain [82]

Holotype: Institut Mediterrani d'Estudis Avançats in Esporles, IMEDEA 90614, m1 (figured in [82], fig. 2J)

Age: 5.333–3.6 Ma (4.467 Ma): early Pliocene [81, 82, 273]

Anatomical references: [81, 82]

Quaternary taxa

Allocricetulus Argyropulo, 1933

There are 2 species but 1 of them, *Allocricetulus curtatus* (Allen, 1925), is not included here, as its status as a species is currently under debate [9]. The type species is included here:

Allocricetulus eversmanni (Brandt, 1859)

Geographical distribution of fossil occurrences: Moldova, Romania, Russia, and Ukraine

Type locality: near Orenburg, Russia [3, 77, 274]

Distribution: Southern Russia, Northern and Eastern Kazakhstan, North-Western China

Oldest fossil occurrence: Gura Dobrogei 4-2, Romania: 0.374–0.125 Ma: Saalian [117]

Observed material: French National Museum of Natural History, Paris (MNHN) 2024-1556–1558: 3 skulls with mandibles from Kokehada, Xinjiang, China; 1958-748: skull with mandible from Russia; 1961-711: skull from Russia

Anatomical references: [3, 7, 32]

Remarks: *Allocricetulus* and †*Allocricetus* have been synonymized by some authors (see [3]). There is ‘*Allocricetus eversmani*’ mentioned in Cai et al. [195] and in Tjutkova and Kaipova [114]. As these fossils are distinctively older than any other known *A. eversmanni* fossils, they are treated as †*Allocricetus* indet. and are not included here.
In Markova et al. [275], there are different combinations mentioned (‘*Allocricetus eversmanni*’, ‘*Allocricetulus eversmani*’ and *Allocricetulus eversmanni*), while Krokhmal and Rekovets [276] list for the same locations ‘*Cricetulus eversmanni*‘ (and once probably by mistake ‘*Cricetus eversmanni*’).
Uncertainties about †*Allocricetus* vs *Allocricetulus* can also be seen in Serdyuk and Zenin ([277], text vs. fig. 1, [278], tab. 2 vs. fig. 3).

†*Allocricetus* Schaub, 1930

The status of *Allocricetus* as a valid genus is doubted by several authors (see e.g., [70]), due to similarity of their molar morphologies to *Cricetulus*, that can only be separated by proportions of morphotypes [67]. Cuenca-Bescós [13] kept the two genera separated, based on their phylogenetic analysis. According to this analysis, the genus should be split up, with the type species *A. bursae* on the one side and a new genus including *A. ehiki*, *A.*

correzensis (Chaline, 1972), *A. croaticus* Paunovič & Rabeder, 1996 and *A. anterolophidens* on the other side. *Allocricetus jesreelicus* and *A. teilhardi* should be transferred to *Cricetulus*. Two additional species, *A. aylasevimae* and *A. primitivus*, were described later and are included here, as well. We included the following 7 of the 9 mentioned species, excluding *A. correzensis* and *A. croaticus*:

†*Allocricetus aylasevimae* Ünay, de Bruijn & Suata-Alpaslan 2006

Single occurrence/type locality: Çorakyerler, Turkey [279]

Holotype: Department Aardwetenschappen, University Utrecht, no. 54, M2 (figured in [279], pl. 1, fig. 19)

Age: 8.75–7.65 Ma (8.2 Ma)

Anatomical reference: [279]

†*Allocricetus anterolophidens* Topachevsky & Skorik, 1992

Geographical distribution of occurrences: Moldova and Ukraine

Type locality: Zhevakhova Gora 'lower level', Ukraine [32] (= Zhevakhova Gora 11, following [68])

Holotype: NASU-IZAN No. 31, M2 (possibly figured in [32], fig. 67d or e, there is no collection number given for the figured M2s)

Age range: 4.27–2.54 Ma (3.405 Ma)

Oldest occurrence: Luceşti: MN 15–MN 16a [244]

Youngest occurrences: Cherevichnoe 2, Zhevakhova Gora 15: MN 16 [32, 280]

Anatomical references: [13, 32]

†*Allocricetus bursae* Schaub, 1930

Geographical distribution of occurrences: Austria, Bulgaria, China, Croatia, Czech Republic, France, Georgia, Germany, Greece, Hungary, Israel, Italy, Poland, Portugal, Romania, Serbia, Slovakia, Slovenia, Spain, Switzerland, Turkey, and Ukraine

Type locality: 'Fortyogóberg bei Brassó', Romania [59] = Fortyogó or Șprenghi hill (Fortyogó-hegy) near Brașov

Holotype: Schaub [59] did not assign a holotype for *Allocricetus bursae*. There was however one maxilla figured (fig. 13), that is usually taken as holotype (according to [281]).

Age range: 5.47–0.0056 Ma (2.738 Ma)

Oldest occurrence: İğdeli: MN 14 [282, 283]

Youngest occurrence: Buena Pinta Cave 1 [284, 285]

Observed material: FSL: 3 mandibles with m1–3 from Villány

Anatomical references: [13, 32, 59, 67, 98, 112, 281, 283, 286–302]

Remark: Schaub [59] did also not assign a type locality but the figured maxilla (fig. 13), that is usually taken as holotype, and the only figured molars (pl. 1, fig. 9) are from Brașov.

†*Allocricetus ehiki* Schaub, 1930

Geographical distribution of occurrences: Bulgaria, China, Czech Republic, Greece, Hungary, Italy, Moldova, Poland, Russia, Slovakia, and Ukraine

Type locality: Villány, Kalkberg, Hungary [59] (= Villány 3 according to [68, 69], = Villány (? 5) according to [180])

Holotype: Schaub [59] did not assign a holotype for *Allocricetus ehiki*. There is however one lower toothrow figured by him from Villány (pl. 1, fig. 10).

Age range: 5.23–0.13 Ma (2.68 Ma)

Oldest occurrence: Komanos 1 low [180, 303]

Youngest occurrence: Jinpendong [304, 305]

Observed material: FSL: 3 mandibles with m1–3 from Villány, 1 mandible with m1–2

Anatomical references: [13, 32, 59, 67, 98, 99, 112, 295, 306, 307]

Remark: Schaub [59] did also not assign a type locality, but the only figured molars (pl. 1, fig. 10) are from Villány, which was also the locality with the most material of the species, known to him.

†*Allocricetus jesreelicus* Bate, 1943

Geographical distribution of occurrences: Israel

Type locality: Tabun Cave F, Israel [308]

Holotype: NHMUK M 16006, skull with dentition (figured in [308], figs. 4, 5a, b)

Age range: 0.361–0.103 Ma (0.232 Ma)

Oldest occurrence: Oumm Qatafa Cave [309]

Youngest occurrence: Hayonim E [309]

Observed material: FSL: 3 mandibles with m1–3 from Oumm Qatafa Cave

Anatomical references: [13, 308, 309]

Remark: According to Cuenca-Bescós et al. [13], the species should be transferred to *Cricetulus*.

†*Allocricetus primitivus* Wu & Flynn, 2017

Geographical distribution of occurrences: China

Type locality: Yushe-YS4, China [55]

Holotype: IVPP V9895.1, maxilla (figured in [55], fig. 10.4d)

Age range: 4.799–4.3 Ma (4.55 Ma)

Oldest occurrences: Yushe-YS39, 50 [55, 120]

Youngest occurrences: Yushe-YS4, 43 [55, 120]

Anatomical references: [55, 302]

Remark: According to Opdyke et al. [120], Yushe-YS97 would be the youngest occurrence of *A. primitivus* but see the remark of †*Neocricetodon grangeri*.

†*Allocricetus teilhardi* Zheng, 1984 [99]

Geographical distribution of occurrences: China

Type locality: Zhoukoudian 13, China [99]

Holotype: IVPP RV410012, posterior part of skull (figured in [310], fig. 38A, as cited in [99])

Age range: 2–0.374 Ma (1,187 Ma)

Oldest occurrence: Fanchang, Renzidong [54, 311]

Youngest occurrence: Zhoukoudian 1 [99, 312]

Anatomical references: [13, 99, 302]

Remark: According to Cuenca-Bescós et al. [13], the species should be transferred to *Cricetulus*.

Cricetulus Milne-Edwards, 1867

Musser and Carleton [77] list 6 extant species of that genus. Molecular studies suggest however, a non-monophyly of *Cricetulus* in that broad sense [7, 12]. Consequently, the genus is split up (see *Nothocricetulus* and *Urocricetus)* and is assumed to only include 3 extant species, of which 1 is not included here, *Cricetulus sokolovi* Orlov & Malygin, 1988, due to limited available morphological descriptions [313]. Regarding the extinct taxa, 7 species have been referred to '*Cricetinus*' Zdansky, 1928, which is synonymized with *Cricetulus* by several authors (see e.g., [76, 77]). Recently, the type species '*Cricetinus varians*' Zdansky, 1928, has been identified as a subspecies of *Tscherskia triton* (see [54]). The remaining 6 species have been tentatively assigned to different genera [54] but for now, they are referred to as *Cricetulus*, here. Of these 6 species, 1 is not included here, †*Cricetulus janossyi* (Hír, 1996), as no M1 is known to date [80]. In total, the following 7 (of 9) *Cricetulus* species are included in the phylogenetic reconstruction:

Cricetulus barabensis (Pallas, 1773)

Geographical distribution of fossil occurrences: China and Russia

Type locality: South-Western Siberia, Russia [3, 77]

Distribution: Southern Siberia, Mongolia, North-Eastern China, North-Eastern Kazakhstan, North Korea

Oldest fossil occurrence: Yushe-YS120, China: 2.595–2.14 Ma: C2r.2r [55, 120]

Observed material: SNSB-BSPG1973 I 211: skull with mandible from an unknown locality. MNHN 1867-152: skull with mandible from Shuen hoa fou, China; 1868-1384: skull with single broken hemimandible, from Siberia, Russia; 1962-2345: skull with mandible from Peking, China.

Anatomical references: [3, 7, 54, 55, 314]

Cricetulus longicaudatus (Milne-Edwards, 1871)

Geographical distribution of fossil occurrences: China

Type locality: Northern Shanxi, near Saratsi, China [3, 77, 315]

Distribution: Russia, East Kazakhstan, North-Western China, Mongolia, Tibet

Oldest fossil occurrence: Zhoukoudian 9, China: 1.376–0.774 Ma: late third of the Early Pleistocene [99, 316]

Observed material: MNHN 2024-1312–1315: 4 skulls with mandibles from Xiji, Ningxia, China; 2024-1368–1371: 4 skulls with mandibles from Ban Ban Wan, Gansu, China; 2024-2250–2253: 4 skulls with mandibles from Huang He, Gansu, China

Anatomical references: [3, 7, 54, 99, 314, 317]

†*Cricetulus beremendensis* (Hír, 1994)

Geographical distribution of occurrences: Hungary

Type locality: Beremend 15, Hungary [52]

Holotype: Paleontological Department, Hungarian Natural History Museum, Budapest, no number given, mandible (figured in [52], figs. 1, 3 as *'Cricetinus beremendensis'*)

Age range: 3.4–2.7 Ma (3.05 Ma)

Oldest occurrence: Csarnóta 4 [52, 318]

Youngest occurrence: Beremend 15 [52, 319]

Anatomical references: [52, 54, 79, 318, 320]

Remark: According to Xie et al. [54], the species should be transferred to †*Allocricetus*.

†*Cricetulus europaeus* (Kretzoi, 1959)

Single occurrence/type locality: Csarnóta 2 [74]

Holotype: Kretzoi [74] did not formally assign a holotype for this species. He did however specifically address the m1 among the three specimens available to him (M1, M2, m1), which might be the reason, Kordos [214] calls it the holotype. Hír [52] lists all three molars as holotype, instead. The M2 is however later identified as †*C. janossyi* [80], leaving the syntypes: Palaeovertebrate Collection of the Hungarian Geological Institute, Budapest, V. 12769, M1 and m1 (figured in [75], pl. 4, figs. 1 and 2, in [214], figs. 1:10 and 5:6, and in [52], figs. 6 and 11; as *Cricetinus europaeus*).

Age: 3.4–3.2 Ma (3.3 Ma) [321]

Anatomical references: [52, 54, 74, 75, 79, 80, 214, 318, 320]

Remark: According to Xie et al. [53, 54], the species should be transferred to *Tscherskia*.

†*Cricetulus gritzai* (Topachevsky & Skorik, 1992)

Single occurrence/type locality: Odessa Catacombs, Ukraine [32]

Holotype: NASU-IZAN No. 35, maxilla (figured in [32], fig. 77, as ‘*Cricetinus gritzai*’)

Age: 4–3.5 Ma (3.75 Ma) [322]

Anatomical references: [32, 54, 79]

Remarks: The measurement of the M3 given by Topachevsky and Skorik [32] is not taken for the morphological matrix. Its length is larger than in the M2, disagreeing with their diagnosis of ‘*Cricetinus*’. As there is only one M3, this might be a statistical error.

According to Xie et al. [53, 54], the species should be transferred to *Tscherskia*.

The species is also mentioned as ‘*C. grizai*’ [78], ‘*C. grizaii*’ [322] and ‘*C. gritzaii*’ [191].

There is an additional locality called ‘Angangxi_Daxingtun’, listed for this species in The

NOW database [78]. The given reference for this occurrence mentions only *C.* cf. *griseus* however (reference erroneously given in [78] as: 'Huang & Zhang (1984). Discovery of paleolithic artifacts at Angangxi, Qiqihaer, Heilongjiang. Acta Geologica Sinica 3 (3)'; see instead: [323] in the reference list).

†*Cricetulus koufosi* (Koliadimou, 1996)

Geographical distribution of occurrences: Greece

Type locality: Ravin Vulgarakis, Greece (following [79, 191])

Holotype: Geology-Paleontology Museum of the School of Geology of the Aristotle University of Thessaloniki, RVL289, mandible (figured in [324], fig. 4.38a, as cited in [191])

Age range: 1.9–1 Ma (1.45 Ma)

Oldest occurrence: Marathoussa [79, 325]

Youngest occurrence: Ravin Vulgarakis [79, 325]

Anatomical references: [54, 79, 191]

Remark: According to Xie et al. [53, 54], the species should be transferred to *Tscherskia*.

†*Cricetulus mesolophidos* (Wu & Flynn, 2017)

Geographical distribution of occurrences: China

Type locality: Yushe-YS97, China [55]

Holotype: IVPP V9885.1, m1 (figured in [55], fig. 10.3i, as '*Cricetinus mesolophidos*')

Age range: 4.799–2.6 Ma (3.7 Ma)

Oldest occurrence: Yushe-YS50 [55, 120]

Youngest occurrences: Lingtai, Wenwanggou(93001)-4; Lingtai, Xiaoshigou-4 [267]

Anatomical references: [53–55]

Remarks: According to Xie et al. [53, 54], the species should be transferred to †*Neocricetodon*.
For a comment on the age of Yushe-YS97, see the remark of †*Neocricetodon grangeri*. According to Wu and Flynn [55], the locality Yushe-YS59 should not be taken into account in the stratigraphic range of this taxon, as the specimen might be recent. The age of the locality is uncertain. According to Opdyke et al. [120], fig. 4.8, it is in the Mahui Formation but according to Wu and Flynn [55], it is in the Taoyang Member of the Gaozhuang Formation. Either way, this age would be considerably older than the remaining localities of †*C. mesolophidos*. On the other hand, following Tedford et al. [219], fig. 3.4, Yushe-YS59 is close in age to Yushe-YS58. Yushe-YS58 is correlated to C3n.2n: 4.631–4.493 Ma, which fits to the age range of †*C. mesolophidos* and is the assumed age of Yushe-YS59, here.

Cricetus Leske, 1779

The only extant species, the type species *Cricetus cricetus* is included in the analysis. Regarding extinct species, there is †*C. lophidens* from the Late Miocene or Early Pliocene. Other species from the Neogene, that have been previously assigned to *Cricetus*, were however transferred to different genera, later (†*Apocricetus* and †*Pseudocricetus* [32, 61]). Additionally, there are 4 taxa from the Pleistocene, †*C. major* (Woldřich, 1880), †*C. nanus* (Schaub, 1930), †*C. praeglacialis* (Schaub, 1930) and †*C. runtonensis* (Newton, 1909). The status of these taxa has been discussed extensively over the last century (see summaries in [90, 91]). The main questions regard the species or subspecies level of the 4 taxa [69, 90, 92] and the status of the 2 largest forms, †*C. runtonensis* and †*C. major* [93–95]. Of the 6

possible total species, *C. cricetus* and †*C. lophidens* are included in this analysis, while the latter might belong to a different genus (see [89]):

Cricetus cricetus (Linnaeus, 1758)

Geographical distribution of fossil occurrences: Armenia, Austria, Belarus, Belgium, Bulgaria, Croatia, Czech Republic, France, Georgia, Germany, Hungary, Israel, Italy, Moldova, Montenegro, Poland, Romania, Russia, Serbia, Slovakia, Slovenia, Switzerland, Turkey, and Ukraine

Type locality: Germany [326]

Extant distribution: Central and Eastern Europe, Western Siberia, Northern Kazakhstan, North-Western China

Oldest fossil occurrences: Salcia 1, 2, Moldova: 1.785–1.585 Ma: MIS 62 –41 [276, 327]

Observed material: SNSB-BSPG 1991 IV 1–95: 95 skulls with mandibles from Rhine Hesse, Germany (‘Raum Alsheim, Wintersheim, Dorn Dürkheim’). Bavarian State Collection for Zoology, Munich (SNSB-ZSM) 1988/0061, 0068, 0069, 0072, 0076, 0080, 0095, 0107, 0108, 0111, 0120–0122, 0125, 0129, 0131–0133, 0140, 0141, 0145: 21 skulls with mandibles from the above-mentioned location.

Anatomical references: [3, 90, 93, 95]

†*Cricetus lophidens* de Bruijn, Dawson & Mein, 1970

Single occurrence/type locality: Maritsa 1, Greece [87]

Holotype: Department Aardwetenschappen, University Utrecht, MA-16, M1 (figured in [87], pl. 4, fig. 3 and in [191], fig. 22a)

Age: 5.4–5.23 Ma (5.315 Ma)

Observed material: FSL: 2 M1, M2, 2 M3, m1, 3 m2, m3 from Maritsa

Anatomical references: [87, 89]

Remarks: The assignment to *Cricetus* is apparently doubted by de Bruijn et al. [89], as they put the genus in quotation marks (“*Cricetus*” *lophidens*)
The age of Maritsa 1 is determined as either Late Miocene, MN 13 (see e.g. [180, 191]) or Early Pliocene, MN 14 (see e.g. [223, 328]) (see also [210, 282, 329]). For absolute ages, Koufos and Vasileiadou [303] are followed, who suggest an age of 5.4–5.23 Ma for Turolian/Ruscinian localities.

Mesocricetus Nehring, 1898 [330]

Of the 4 extant species, only *Mesocricetus raddei* (Nehring, 1894) is not included in this analysis. Regarding the extinct species, there are 2, †*M. rathgeberi* Pieper, 1984, which is however only known from one skull fragment including its upper tooth row [331] and †*M. primitivus*, which is included here. Recently, an additional species, *Mesocricetus fengi* Qiu, Flynn, Wang & Li, 2026, was described [263]. Due to the recency, the species could not be incorporated in this study. Another possible species is †*’M. aramaeus’* Bate, 1943, which might be a synonym of *M. auratus* (see [332] vs. [333, 334]). In Erbajeva and Alexeeva [335], †*’Mesocricetus* cf. *primaevus’* is mentioned (see also [78]), there is, however, no other mentioning of this species known to the authors. For the same locality, *’Mesocricetus* sp.*’* is listed in a more recent publication [336]. A similar case is †*’Mesocricetus armatus’*, mentioned in Turnbull [337] (see [3, 338]).

Mesocricetus auratus (Waterhouse, 1839)

Geographical distribution of fossil occurrences: Israel, Turkey

Type locality: Aleppo, Syria [339]

Extant distribution: around Aleppo, Syria, South-Eastern Turkey

Oldest fossil occurrence: Dursunlu, Turkey: 0.99–0.78 Ma [110, 111]

Observed material: Natural History Museum, London, United Kingdom (NHMUK) 1992,42: CT scan of a skull with mandible. SNSB-ZSM 1951/0062, 0162; 1952/0248; 1953/0197; 1954/0046; 1955/0160, 0160a; 1971/0613; 1972/0331: 9 skulls with mandibles bred in Munich.

Anatomical references: [3, 340–344]

Mesocricetus brandti (Nehring, 1898) [345]

Geographical distribution of fossil occurrences: Armenia, Georgia, Iran, Turkey

Type locality: Transcaucasian [345], 'Marienfeld' near Tbilisi, Georgia [3, 77, 346]

Extant distribution: Turkey, Armenia, Georgia, Azerbaijan, South to North-Western Iran, and Dagestan, Russia

Oldest fossil occurrence: Kaldar Cave 5, Iran: 0.071–0.057 Ma: MIS 4 [347, 348]

Observed material: SNSB-ZSM 1970/0007: 1 skull (braincase damaged), 2 maxilla fragments, 6 mandibles from a cave South-East of Divanlar, Wilkonya, Central Anatolia, Turkey

Anatomical references: [3, 341, 348–351]

Mesocricetus newtoni (Nehring, 1898) [345]

Geographical distribution of fossil occurrences: Bulgaria, Greece, Montenegro, Romania, and Serbia

Type locality: Schumla [346] = Kolarovgrad, Bulgaria [77]

Extant distribution: South-Eastern Romania, Northern Bulgaria

Oldest fossil occurrence: Kozarnika B1 (11a), Bulgaria: 0.75–0.62 Ma [352, 353]

Anatomical references: [3, 294, 342, 354–359]

†*Mesocricetus primitivus* de Bruijn, Dawson & Mein, 1970

Geographical distribution of occurrences: Greece, Israel, and Turkey

Type locality: Maritsa 1, Greece [87]

Holotype: Department Aardwetenschappen, University Utrecht, MA-113, m2 (figured in [87], pl. 4, fig. 10 and in [191], fig. 20e)

Age range: 5.4–1.2 Ma (4.11 Ma)

Oldest occurrences: Maritsa 1, Silata [191, 303]

Youngest occurrence: Ubeidiya [333, 360]

Observed material: FSL: 2 M1 from Ubeidiya II-23

Anatomical references: [70, 87, 89, 109, 191, 361–363]

Nothocricetulus Lebedev, Bannikova, Neumann, Ushakova, Ivanova & Surov, 2018

Based on molecular data, Lebedev et al. [7] established a new genus, *Nothocricetulus*, for the former *'Cricetulus migratorius'* (see also [12]). This only species of the genus is included in the analysis:

Nothocricetulus migratorius (Pallas, 1773)

Geographical distribution of fossil occurrences: Armenia, Azerbaijan, Belgium, Bosnia and Herzegovina, Bulgaria, France, Georgia, Germany, Greece, Hungary, Iran, Iraq, Israel, Italy, Kyrgyzstan, Moldova, Montenegro, Netherlands, Poland, Romania, Russia, Serbia, Slovenia, Turkey, and Ukraine

Type locality: Ural River, South-West of Orenburg, Russia [3]

Extant distribution: Greece, Romania, Bulgaria, Southern European Russia, Kazakhstan, Southern Mongolia, Northern China, Turkey, Israel, Jordan, Lebanon, Iraq, Iran, Afghanistan, Northern India

Oldest fossil occurrence: Tourkovounia 2, Greece: 1.9–1.5 Ma [325, 364]

Observed material: SNSB-BSPG 1977 VI: 21 mandibles, 3 maxilla fragments from the surroundings of Kabul. SNSB-ZSM 1911/2253: skull with mandible from Pakistan (originally determined as '*Cricetulus phaeus*').

Anatomical references: [3, 7, 32, 54, 67, 341, 358, 365, 366]

Phodopus Miller, 1910

All 3 species of this genus are included in this analysis. Recently, Kryštufek and Shenbrot [3] split the genus in two, with *Phodopus campbelli* and *P. sungorus* as *'Cricetiscus'* Thomas, 1917, resulting in a monospecific *Phodopus* including only *P. roborovskii* (following [12]). A potential fossil species, †*'P. minutus'* Tjutkova, 1992, is described from the Upper Pliocene of Kazakhstan but based solely on a single m1 [367]. It is therefore neglected in this study. Other fossil specimens could not be identified at species level with certainty (see e.g. [55, 195]).

Phodopus campbelli (Thomas, 1905)

Type locality: Shaborte, Mongolia [77, 368] = Zhenglanqi, Xilin Gol, Nei Mongol, China [3]

Extant distribution: Mongolia, North-Eastern China, Transbaikalia in Russia

Observed material: SNSB-BSPG 1985 I: skull with mandible from an unknown locality. MNHN 1986-597: skull with mandible from Russia.

Anatomical references: [3, 369]

Phodopus roborovskii (Satunin, 1902)

Type locality: upper part of Scharogol-Dzhin, Nan Shan, Qinghai, China [3, 77, 370]

Extant distribution: Tuva in Russia, Eastern Kazakhstan, Southern and Western Mongolia, China, Eastern India

Observed material: SNSB-BSPG 1970 I 7–8: skull with mandible from the Flaming Cliffs site (‘Bain-Dzak’), Mongolia. MNHN 1960-3699, 1960-3700: 2 skulls with mandibles from Northern China; 1986-1103: skull with mandible from Touva, Tere-Khol Lake, Russia; 1997-1295, 1997-1298: 2 skulls with mandibles from Nemegt, omnogobi, Mongolia.

Anatomical references: [3, 55, 317]

Phodopus sungorus (Pallas, 1773)

Type locality: near Grachevskiy, Kazakhstan [3, 77]

Extant distribution: Northern and Eastern Kazakhstan and South-Western Siberia, Russia

Observed material: SNSB-BSPG 1985 I: skull with mandibles from an unknown locality. SNSB-ZSM 1972/0207,0477; 1974/0002: 3 skulls with mandibles bred in Munich. MNHN 1848-346: skull with one hemi-mandible from Siberia, Russia; 1958-301, 1961-883: 2 skulls

with mandibles from Russia; 1986-1102: skull with mandible from Dimitrievka, Siberia, Russia.

Anatomical references: [3, 32, 55, 69]

Tscherskia Ognev, 1914

The type species of the genus, *Tscherskia triton*, is included in the analysis. Recently, a second extant taxon was considered as a valid species, *T. ningshaanensis* (Song, 1985), or *T. collina* (Allen, 1925) (see [3, 8]). Regarding fossil specimens, '†*Cricetinus varians*' is treated as a subspecies of *T. triton* [54]. Additionally, several species of the genus *Cricetulus* (or *'Cricetinus'* respectively) were recently tentatively transferred to *Tscherskia* (see [53, 54]). They are here listed under *Cricetulus*. There is one extinct species described from the Holocene of Iran, †*'T. rusa'* Storch, 1974. The status of the species and its genus assignment are however questionable [54] and they are therefore neglected, here.

Tscherskia triton (de Winton, 1899)

Geographical distribution of fossil occurrences: China

Type locality: Northern Shandong, China [3, 77, 371]

Extant distribution: Upper Ussuri, Russia; North-Eastern China, Korean Peninsula

Oldest fossil occurrence: Zhoukoudian 9, China: 1.376–0.774 Ma: late third of the Early Pleistocene [99, 316]

Observed material: MNHN 2024-2553: skull with mandible (one m3 isolated) from Xiji, Ningxia, China; 2024-1818–1830: 13 skulls with mandibles (m3s missing in 2024-1819, all third molars missing in 2024-1830) from Ban Ban Wan, Gansu, China.

Anatomical references: [3, 54]

Urocricetus Satunin, 1902

The changes in the intergeneric taxonomy over the last decades are summarized in Kryštufek and Shenbrot [3]. Currently two species are recognized: the type species *Urocricetus kamensis*, which is included here, and *U. lama* (Bonhote, 1905) (see also [9]). To date, there is no fossil record known.

Urocricetus kamensis Satunin, 1902

Type locality: Mokchu River, Tibet, China [3, 77, 370]

Extant distribution: Tibet and surrounding areas, China

Observed Material: MNHN 2024-1289–1306: 18 skulls with mandibles (2024-1305 broken) from Siqhu, Sichuan, China.

Remark: The specimen used to describe the species and genus (subgenus in [370]) had completely worn teeth, which is why no molar descriptions are given [370].
