# Supplementary material for: Total-evidence phylogeny reveals recent crown group radiation and biogeographical history of hamsters
Source: BMC Biol. 2026 Apr 9;24:117. doi: 10.1186/s12915-026-02581-z (PMC13173810; doi:10.1186/s12915-026-02581-z)
Supplement: Supplementary file 4 — Additional file 4: Text document (.pdf) containing the GenBank accession numbers of sequences used for the phylogenetic analyses, including explanations and references for chosen sequences. [file 12915_2026_2581_MOESM4_ESM.pdf]

Additional file 4 for

Total-evidence phylogeny reveals recent crown group radiation and biogeographical history of hamsters

Moritz Dirnberger[1], Pablo Peláez-Campomanes[2], Tiago R. Simões[3], Raquel López-Antoñanzas[1, 2]

[1]Institut des Sciences de l'Évolution de Montpellier, Université de Montpellier, CNRS, IRD, 34095 Montpellier, France

[2]Departamento de Paleobiología, Museo Nacional de Ciencias Naturales-CSIC, Madrid, Spain

[3]Department of Ecology and Evolutionary Biology, Princeton University, Briger Hall, Princeton-NJ, 08544, USA.

GenBank accession numbers for the total-evidence analysis

For the molecular part of the total-evidence matrix, sequences were downloaded from GenBank [396], with accession numbers given in Table S3.

Table S3. GenBank accession numbers of the sequences used for the phylogenetic reconstruction of this study.

Species	Genes					
	cytb [1]	*vwF* [1]	*IRBP* [2]	*GHR* [2]	*RAG1* [2]	*BRCA1* [2]
Allocricetulus eversmanni	AJ973378	AM000037	MG685575	MG685554	MG685595	MG685515
Cricetulus barabensis	AJ973384	AM000043	**MG685585**	**MG685564**	**MG685605***	**MG685525**
Cricetulus longicaudatus	AJ973386	AM000045	MG685581	MG685560	MG685601*	MG685521
Cricetus cricetus	AJ973392	AM000051	MG685574	MG685553	MG685594	MG685514
Mesocricetus auratus	AJ973379	AM000038	AY163591[3]	AF540632[4]	AY294955[5]	AY295013[5]

Mesocricetus brandti	AJ973380	AM000039	MG685572	MG685551	MG685592	MG685512
Mesocricetus newtoni	AJ973381	AM000040	-	-	-	-
Nothocricetulus migratorius	AJ973387	AM000046	MG685577	MG685556	MG685597	MG685517
Phodopus campbelli	AJ973389	AM000048	-	-	-	-
Phodopus roborovskii	AJ973391	AM000050	MG685571	MG685550	MG685591	MG685510
Phodopus sungorus	AJ973390	AM000049	KC953439[6]	AF540640[4]	AY294954[5]	AY295012[5]
Tscherskia triton	AJ973388	AM000047	MG685573	MG685552	MG685593	MG685513
Urocricetus kamensis	MG685546[7]	MG685616[7]	MG685569	MG685548	MG685590	MG685528

The sequences in bold of *IRBP, GHR, RAG1, BRCA1* for *Cricetulus barabensis* are taken from subspecies *C. b. pseudogriseus*, following Jiang et al. [8].

*The accession numbers given in Lebedev et al. [7] for *RAG1* of *C. b. pseudogriseus* and *C. longicaudatus* refer to other species and genes in GenBank. Therefore, MG685605 and MG685601 are taken, which are also created by Lebedev et al. [7] (but not mentioned in their paper).

[1] *cytb*, *vwF* from Neumann et al. [12], except for *U. kamensis*

[2] *IRBP*, *GHR*, *RAG1*, *BRCA1* from Lebedev et al. [7], except for *M. auratus* and *P. sungorus* (here, see [399])

[3] Weksler [397]

[4] Adkins et al. [398]

[5] Steppan et al. [101]

[6] Schenk et al. [399]

[7] Lebedev et al. [7]
